# Differential benefit of adjuvant everolimus according to endocrine therapy backbone in the randomized UNIRAD trial

**DOI:** 10.1016/j.esmoop.2025.105050

**Published:** 2025-04-15

**Authors:** M. Saint-Ghislain, S. Chabaud, F. Dalenc, D. Allouache, D. Cameron, M. Martinez, J. Grenier, P. Barthelemy, M. Brunt, L. Kaluzinski, A. Mailliez, E. Legouffe, A.-C. Hardy-Bessard, S. Giacchetti, M.-A. Mouret-Reynier, J.-L. Canon, J. Bliss, J. Lemonnier, F. Andre, T. Bachelot, P. Cottu

**Affiliations:** 1 Institut Curie, Paris, France; 2 Centre Oscar Lambret, Lille, France; 3 Centre Léon Bérard, Lyon, France; 4 Institut Universitaire de Cancérologie de Toulouse – Institut Claudius Régaud, Toulouse, France; 5 Centre François Baclesse, Caen, France; 6 Institute of Genetics and Cancer, University of Edinburgh, Edinburgh, UK; 7 Clinique Pasteur, Toulouse, France; 8 Institut Sainte Catherine, Avignon, France; 9 Institut de Cancérologie Strasbourg Europe, Strasbourg, France; 10 Royal Stoke Hospital, Stoke-on-Trent, UK; 11 Centre Hospitalier du Cotentin, Cherbourg en Cotentin, France; 12 Centre Oncogard, Nîmes, France; 13 Centre CARIO-HPCA, Plérin, France; 14 Hôpital Saint Louis, Paris, France; 15 Centre Jean Perrin, Clermont-Ferrand, France; 16 Grand Hôpital de Charleroi, Charleroi, Belgium; 17 The Institute of Cancer Research, London, UK; 18 UNICANCER, Paris, France; 19 Gustave Roussy, Villejuif, France; 20 Université Paris-Cité, Paris, France

**Keywords:** luminal breast cancer, everolimus, premenopausal, adjuvant therapy

## Abstract

**Background:**

The randomized, double-blind UNIRAD trial evaluating the addition of 2 years of everolimus to endocrine therapy in patients with high-risk, early luminal breast cancer failed to demonstrate a benefit. We report the subgroup analyses.

**Patients and methods:**

We randomly assigned 1278 patients in a 1 : 1 ratio to receive 2 years of placebo or everolimus, added to endocrine therapy for up to 4 years after initiation. Randomization was stratified by endocrine therapy agent, prior adjuvant versus neoadjuvant therapy, progesterone receptor expression, and lymph node involvement. Subgroup analyses by each stratification factor were pre-specified. *Post hoc* analyses were carried out according to menopausal status and age. Treatment adherence was also analyzed.

**Results:**

We observed a limited trend toward more favorable prognostic features in tamoxifen-treated patients, with more frequent estrogen receptor-positive/progesterone receptor-positive tumors (88.5% versus 84.1%, *P* = 0.026) and less frequent pN2-positive status (39.8% versus 46.0%, *P* = 0.032). In premenopausal women, we observed a numerical benefit of everolimus: 3-year disease-free survival was 86% in the placebo group and 90% in the everolimus group (hazard ratio 0.76, 95% confidence interval 0.43-1.34). In premenopausal patients treated with tamoxifen (*n* = 153; 12.3%), we observed an even stronger trend in favor of everolimus as 3-year DFS was 84% in the placebo group and 91% in the everolimus group (hazard ratio 0.54, 95% confidence interval 0.28-1.02). Early discontinuation of either everolimus or placebo was less frequent in the tamoxifen group than in the aromatase inhibitor group: 48.0% versus 56.9% (*P* = 0.028).

**Conclusions:**

The present *post hoc* analyses generate hypotheses regarding the interaction between menopausal status, tamoxifen, and everolimus in patients with high-risk, ER-positive, human epidermal growth factor receptor type 2-negative early breast cancer. They suggest that tamoxifen alone is an underpowered endocrine treatment in high-risk premenopausal patients.

## Highlights

• In the UNIRAD trial, a benefit of everolimus when added to tamoxifen in high-risk early luminal breast cancer was suggested.
• This trend was most pronounced in premenopausal patients (3-year DFS: 84% versus 91%, hazard ratio 0.54).
• Early discontinuation was less common in patients treated with tamoxifen.
• Tamoxifen alone may be a suboptimal adjuvant therapy in premenopausal women with high-risk early luminal breast cancer.

## Graphical abstract

Graphical abstract

**Figure:**
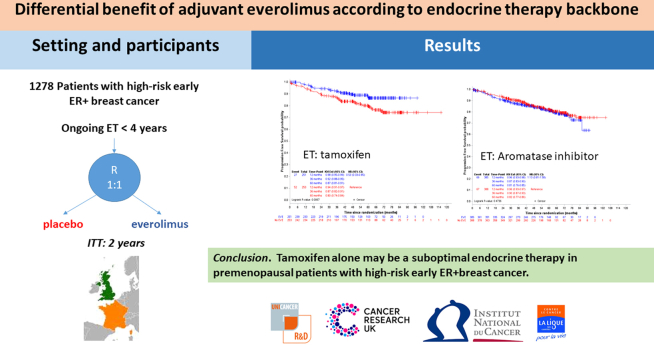

## Introduction

Hormone receptor (HR)-positive, human epidermal growth factor receptor type 2 (HER2)-negative breast cancer accounts for ∼70% of early invasive breast cancer.^1^ Endocrine therapy (ET) is the cornerstone of medical adjuvant therapy and is associated with long-term benefit in both invasive disease-free survival (DFS) and overall survival.^2^ In postmenopausal patients, aromatase inhibitors (AI) for at least 5 years are the standard of care.^3^ In premenopausal patients, at least 5 years of tamoxifen has been the standard adjuvant ET for decades.^4^ More recently, it has been shown that the combination of any ET with ovarian function suppression (OFS) for 5 years significantly improves invasive DFS over tamoxifen alone, especially in very young patients (<35 years old) and in those with high-risk features who usually also require adjuvant chemotherapy.^5^^,^^6^ Unfortunately, ∼20% of all patients with HR-positive, HER2-negative early breast cancer will relapse in the first 10 years with potentially incurable disease. The long-term risk of recurrence is associated with well-documented prognostic features such as tumor size, lymph node involvement, vascular emboli, and tumor proliferation.^7^

Activation of the phosphatidylinositol-3-kinase/Akt/mammalian target of rapamycin (mTOR) signaling pathway has been described as a mechanism of acquired resistance to ET.^8^ In patients with metastatic disease the combination of everolimus and exemestane improved the median progression-free survival from 4.1 months to 10.6 months [hazard ratio: 0.45; 95% confidence interval (CI) 0.38-0.54; *P* = 0.0001] in the randomized phase III BOLERO2 trial.^9^ In the randomized phase II TAMRAD trial, the combination of tamoxifen plus everolimus improved the 6-month clinical benefit rate from 42% in the tamoxifen arm to 61% in the combination arm.^10^

Within this context and before the cyclin-dependent kinase 4 and 6 (CDK4/6) inhibitor era, we initiated UNIRAD, a double-blind, multicenter, international randomized trial comparing the combination of adjuvant everolimus plus standard adjuvant ET versus placebo plus ET in patients with high-risk HR-positive/HER2-negative early breast cancer. The primary results have been reported, suggesting no overall benefit from the addition of 2 years of everolimus to ET.^11^ The pre-specified subgroup analysis of the UNIRAD trial focused on the ET backbone as it appeared to be the only stratification factor with a strong trend towards an association with DFS, suggesting that patients treated with tamoxifen may derive a benefit from the addition of everolimus (hazard ratio 0.62, 95% CI 0.37-1.06, *P* = 0.044).^11^ To further elucidate this intriguing finding, in this report we examine the interaction of ET and allocation to everolimus with age and menopausal status.

## Patients and methods

### Patients

Detailed methods have been reported previously.^11^ Briefly, patients were enrolled if they were women aged ≥18 years with estrogen receptor (ER)-positive, HER2-negtive early breast cancer at high risk of recurrence, as defined by four or more positive lymph nodes; one or more positive lymph node if surgery was carried out after neoadjuvant chemotherapy or ET administered for ≥3 months; or one to three positive lymph nodes at primary surgery and an EPclin® score ≥3.3.

Key non-inclusion criteria were: prior cancer ≤5 years before randomization, significantly impaired lung function, known hypersensitivity to mTOR inhibitors, and any uncontrolled medical condition.

The trial was conducted in accordance with good clinical practice, the Declaration of Helsinki, and all local regulations. Written informed consent was obtained from all patients. The trial was registered on clinicaltrials.gov (NCT01805271). It was sponsored and conducted by UNICANCER Research and Development.

### Study design and conduct of treatment

Patients were randomly assigned 1 : 1 to receive 2 years of placebo or 2 years of everolimus, which was added to ongoing ET for up to 4 years after initiation. Randomization was stratified by ET agent [tamoxifen +/− luteinizing hormone-releasing hormone (LHRH) agonists versus AI], prior adjuvant versus neoadjuvant chemotherapy or ET, progesterone receptor (PR) expression, prior duration of ET (≤3 years versus >3 years), and lymph node involvement (four or more positive lymph nodes and one or more positive lymph node after neoadjuvant treatment versus one to three positive nodes and high EPclin® score). Everolimus dose adjustments have been previously reported.^11^

### Statistical analysis

The primary endpoint of the core analysis was DFS from randomization. New second cancers of non-breast origin were not included. A preplanned subgroup analysis of stratification factors was carried out. Secondary endpoints included overall survival, event-free survival, distant metastasis-free survival, second malignancies, and toxicity. The primary analysis was conducted according to the intention-to-treat principle. For the purpose of this report, *post hoc* analyses were carried out according to menopausal status and age. The Kaplan–Meier method was used to estimate DFS. The hazard ratio and associated 95% CI were calculated using a Cox proportional hazards model. A pre-specified DFS analysis was carried out in stratified subgroups, for which the hazard ratio and 95% CI were calculated by using the Cox model. Data are reported according to the CONSORT 2010 statement.^12^

## Results

### Overall population results

**Figure 1:**
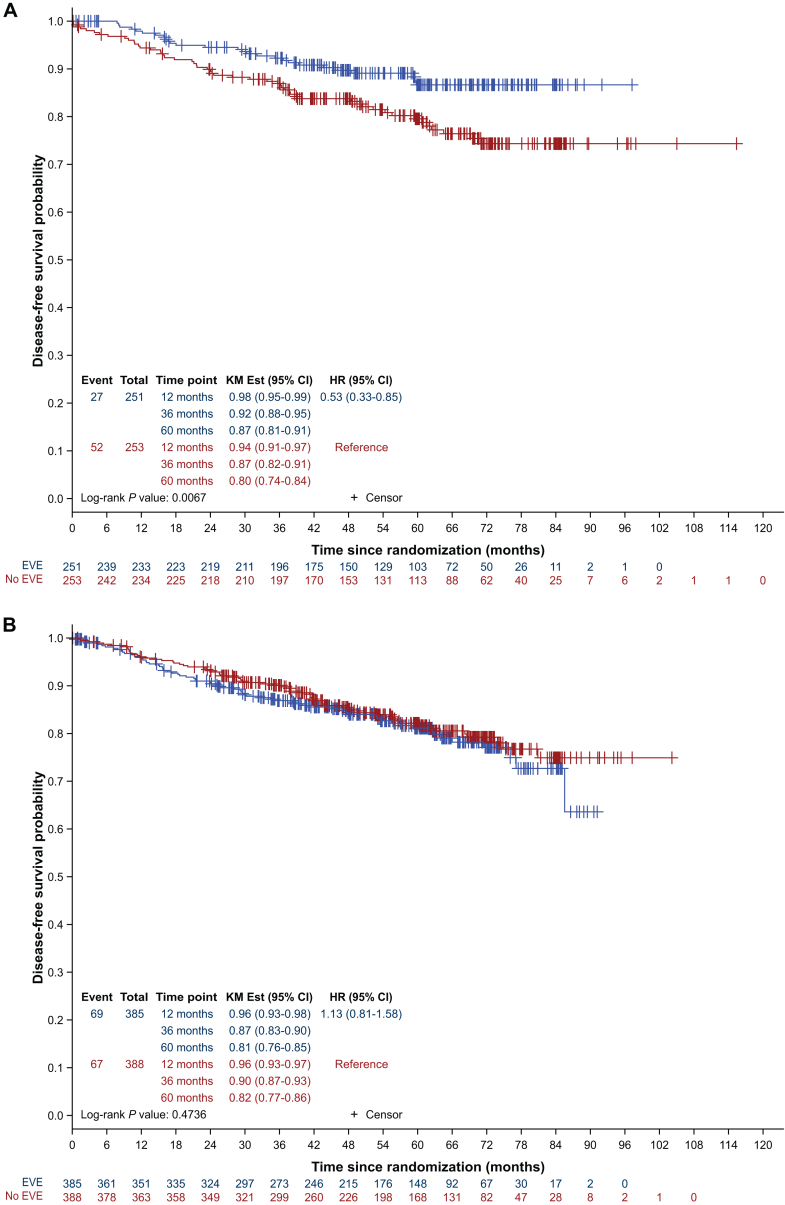

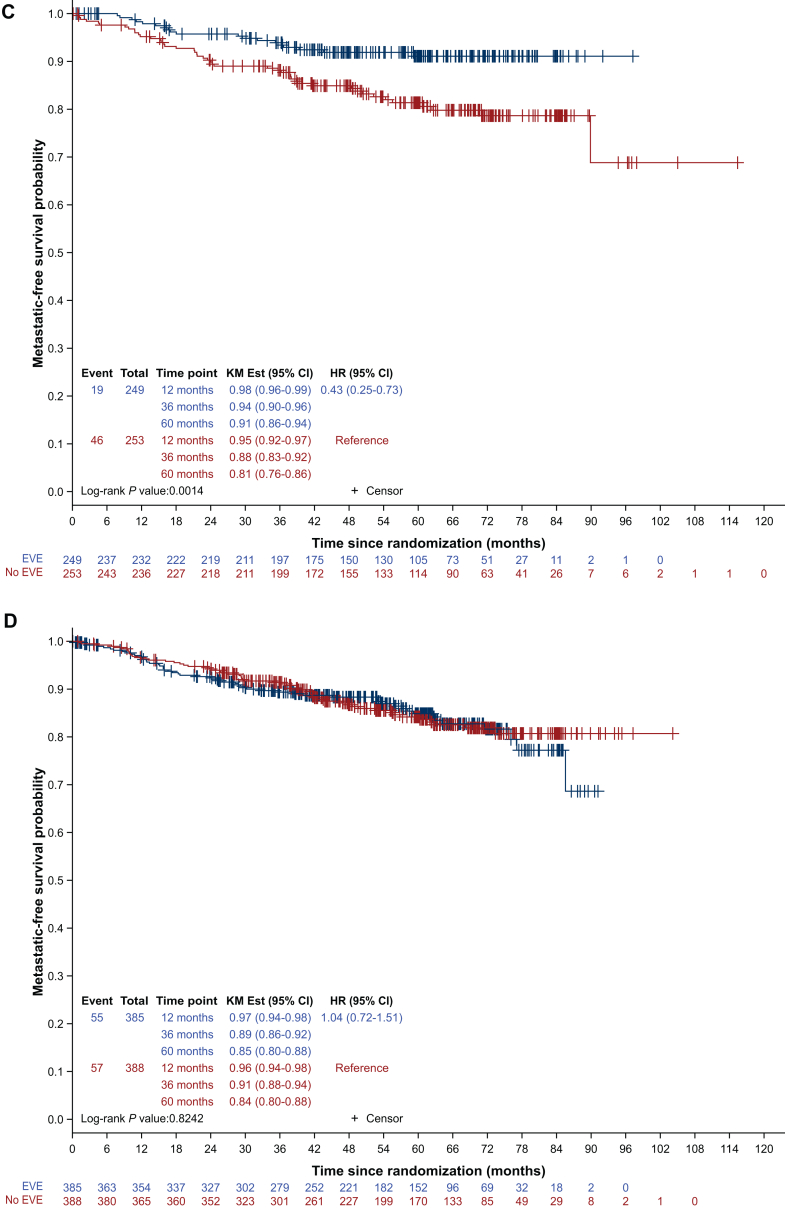
**Subgroup analysis of disease-free survival and distant metastasis-free survival according to endocrine therapy backbone.** Blue curves indicate the everolimus arm; red curves indicate the placebo arm. (A) DFS in tamoxifen subgroup. (B) DFS in the aromatase inhibitor subgroup. (C) DMFS in the tamoxifen subgroup. (D) DMFS in the aromatase inhibitor subgroup. CI, confidence interval; DFS, disease-free survival; DMFS, distant metastasis-free survival; EVE, everolimus; HR, hazard ratio; KM Est, Kaplan Meier estimate.

Between June 2013 and March 2020, 1278 patients were randomized, including 641 patients in the placebo arm, and 637 patients in the everolimus arm. The overall population has been reported previously.^11^ Briefly, the median age was 54 years [interquartile range (IQR) 48-63 years] and 404 and 838 women were premenopausal (31.6%) and postmenopausal (65.8%), respectively (unknown 2.5%). The median duration of ET treatment at randomization was 15 months (IQR 4.9-29.9 months). Endocrine therapies included tamoxifen (43.6%), letrozole (31.7%), anastrozole (18.9%), and exemestane (5.4%). Only seven premenopausal patients (0.5%) received an LHRH agonist, probably due to continued menstruation after adjuvant chemotherapy. In the pre-specified subgroup analysis, ET backbone emerged as the only stratification factor with a strong trend towards an association with DFS (*P* value for interaction = 0.044), suggesting a benefit only in patients receiving tamoxifen.^11^ Other stratification factors such as time to chemotherapy, PR expression, duration of ET at the start of everolimus or nodal involvement were not significantly associated with DFS. These results have been updated, with a median follow-up of 60.3 months (IQR 42.2-71.8 months), compared with 35.7 months in the original report. In the subgroup of patients receiving tamoxifen, DFS at 60 months was 87% (95% CI 81-91 months) in the everolimus arm and 80% (95% CI 74% to 84%) in the placebo arm (hazard ratio 0.53, 95% CI 0.33-0.85, *P* = 0.0067) (Figure 1A). Conversely, in the AI subgroup, 60-month DFS was 81% (95% CI 76% to 85%) in the everolimus arm and 82% (95% CI 77% to 86%) in the placebo arm (hazard ratio 1.13, 95% CI 0.81-1.58, *P* = 0.4736) (Figure 1B). Similar results were observed for distant metastasis-free survival (Figure 1C and D).

To investigate this puzzling interaction further, we developed three approaches. First, we looked for potentially different characteristics in these two subsets of patients (i.e. tamoxifen or AI ET). Second, we examined the interaction of menopausal status and age with DFS and treatments. Finally, we analyzed the relationship between dosing and compliance with everolimus therapy and either patient characteristics or ET treatment.

### Subpopulations

We re-analyzed the study population according to ET backbone (Table 1). Overall, no major differences in high-risk characteristics were observed between the two groups. We observed, however, a limited trend towards more favorable prognostic features in patients treated with tamoxifen, who were more likely to have tumors expressing ER and PR (ER-positive/PR-positive, 88.5% versus 84.1%, exploratory *P* value = 0.026) and less likely to have pN2-positive lymph node involvement (49.6% versus 57.3%, exploratory *P* value = 0.022) than AI-treated patients. As expected, age and menopausal status differed, with patients receiving an AI being significantly older and more likely to be postmenopausal.

**Table 1:** Population characteristics according to endocrine therapy

	Tamoxifen *n* = 505	Aromatase inhibitor *n* = 773	*P* value
Age (years)			<0.001
Median (min-max)	47 (28-73)	60 (31-89)	
Menopause, *n* (%)			<0.0001
Yes	154 (31.7)	688 (90.8)	
No	332 (68.3)	70 (9.2)	
Missing	19	15	
Tumor characteristics, *n* (%)			
ER+PR+	447 (88.5)	650 (84.1)	0.026
ER+/PR−	58 (11.5)	123 (15.9)	
≥4 N+	250 (49.7)	443 (57.5)	0.019
1-3 N+ and EPclin® score ≥3.3	183 (36.4)	229 (29.9)	
1-3 N+ after neo-adjuvant treatment	70 (13.9)	98 (12.7)	
Missing	2	3	
Chemotherapy, *n* (%)			0.834
Neoadjuvant	132 (26.1)	198 (25.6)	
Adjuvant	373 (73.9)	575 (74.4)	
Duration of endocrine therapy (years)			0.132
0-1	227 (45.0%)	304 (39.4%)	
2-3	204 (40.5%)	347 (44.9%)	
>3	73 (14.5%)	121 (15.7%)	
Missing	1	1	
Treatment arm, *n* (%)			0.973
Placebo	253 (50.1)	388 (50.2)	
Everolimus	252 (49.9)	385 (49.8)	

### Disease-free survival according to endocrine treatment, menopausal status, and age

In this *post hoc* unplanned subgroup analysis, we examined the interaction between ET backbone, menopausal status, and age. In premenopausal women, we observed a non-statistically significant numerical benefit of everolimus: 3-year DFS was 86% (95% CI 79% to 91%) in the placebo group and 90% (95% CI 83% to 94%) in the everolimus group (hazard ratio 0.76, 95% CI 0.43-1.34, *P* = 0.3432), while no difference was observed in postmenopausal patients: 3-year DFS was 90% (95% CI 86% to 93%) in the placebo group and 88% (95% CI 84% to 91%) in the everolimus group (hazard ratio 1.04, 95% CI 0.70-1.55, *P* = 0.8451) (Supplementary Figure S1A and B, available at https://doi.org/10.1016/j.esmoop.2025.105050, respectively). Interestingly, in premenopausal patients treated with tamoxifen (*n* = 332; 26.7%), we observed an even greater trend in favor of everolimus, as 3-year DFS was 84% (95% CI 76% to 89%) for the placebo group and 91% (95% CI 84% to 95%) for the everolimus group (hazard ratio 0.54, 95% CI 0.28-1.02, *P* = 0.0521, Supplementary Figure S1C, available at https://doi.org/10.1016/j.esmoop.2025.105050). No benefit of everolimus was observed in postmenopausal patients treated with tamoxifen (Supplementary Figure S1D, available at https://doi.org/10.1016/j.esmoop.2025.105050). Conversely, we observed an intriguing potential detrimental effect of everolimus in the subgroup of 70 premenopausal patients treated with an AI, as the 3-year DFS was 95% (95% CI 68% to 99%) for the control group and 85% (95% CI 65% to 94%) for the everolimus group (hazard ratio 4.78, 95% CI 0.96-23.82, *P* = 0.0355), while again no difference was observed in postmenopausal women treated with an AI (Supplementary Figure S1E and F, available at https://doi.org/10.1016/j.esmoop.2025.105050, respectively). This small subset of patients had a median age of 47.5 years (range 31-59 years; mean 46 years) and 20 of them had also received tamoxifen as part of their ET. Finally, age groups were not associated with any trend, whether considering patients aged <45 years (hazard ratio 0.90, 95% CI 0.45-1.80, *P* = 0.7662) or ≥45 years (hazard ratio 0.97, 95% CI 0.67-1.40, *P* = 0.8810) (Supplementary Figure S2A and B, available at https://doi.org/10.1016/j.esmoop.2025.105050, respectively).

### Dose of treatment and adverse events

Finally, to explain these differences, we examined whether dosing and compliance with everolimus therapy might be associated with either patient characteristics or ET backbone (Table 2). In the everolimus and tamoxifen group, 84 patients (33.3%) started everolimus at 10 mg and 165 (65.5%) at 5 mg, compared with 35.3% and 61.3%, respectively, in the AI group. Adverse events are detailed in Supplementary Tables S1 and S2, available at https://doi.org/10.1016/j.esmoop.2025.105050 by system organ class.

**Table 2:** Everolimus dosing and adverse events according to endocrine therapy

	Tamoxifen *n* = 252	Aromatase inhibitor *n* = 385	*P* value
Initial everolimus dosing (mg), *n* (%)			0.25
0	2 (0.8)	11 (2.9)	
2.5	0 (0.0)	1 (0.3)	
5	163 (64.9)	237 (61.6)	
10	86 (34.3)	136 (35.3)	
Missing	1	0	
Early permanent discontinuation, *n* (%)			0.023
No	88 (34.9)	102 (26.5)	
Yes	164 (65.1)	283 (73.5)	
At least one dose reduction, *n* (%)			0.90
No	163 (64.7)	251 (65.2)	
Yes	89 (35.5)	134 (34.8)	
At least one AE^a^, *n* (%)			0.29
No	7 (2.8)	17 (4.4)	
Yes	245 (97.2)	368 (95.6)	
At least one grade 3-5 AE^a^, *n* (%)			0.67
No	176 (69.8)	275 (71.4)	
Yes	76 (30.2)	110 (28.6)	
When treatment was initiated at 5 mg^a^, *n* (%)			0.82
No	122 (75.2)	175 (73.8)	
Yes	41 (24.8)	62 (26.2)	
When treatment was initiated at 10 mg^a^, *n* (%)			0.42
No	51 (59.3)	88 (64.7)	
Yes	35 (40.7)	48 (35.3)	
At least one AE leading to treatment withdrawal^a^, *n* (%)			0.11
No	187 (74.2)	263 (68.3)	
Yes	65 (25.8)	122 (31.7)	
At least one treatment related AE^a^, *n* (%)			0.001
No	17 (6.7)	58 (15.1)	
Yes	235 (93.3)	327 (84.9)	
Serious adverse events^a^, *n* (%)			0.82
No	224 (88.9)	340 (88.3)	
Yes	28 (11.1)	45 (11.7)	
Maximum grade of adverse events^a^, *n* (%)			0.15
0	7 (2.8)	14 (4.4)	
1	27 (10.7)	43 (11.2)	
2	142 (56.3)	215 (55.8)	
3	68 (27.0)	104 (27.0)	
4	8 (3.2)	3 (0.8)	
5	0 (0.0)	3 (0.8)	

AE, adverse event.

a On safety population (everolimus dose >0).

Briefly, expected grade 3/4 adverse events were more common in the everolimus arm, regardless of ET backbone, and included mucositis, pneumonitis, and increases in total cholesterol, triglycerides or glycemia (Supplementary Table S3, available at https://doi.org/10.1016/j.esmoop.2025.105050). In the tamoxifen plus everolimus arm, 245 patients (97.6%) experienced at least one adverse event, including 78 patients (31.1%) with grade 3/4 adverse events. We observed grade 3 mucositis, elevated liver functional tests, and hypertriglyceridemia in 7.5%, 2.8% and 3.6% of patients, respectively. Four patients (1.6%) experienced grade 3 pneumonitis. Grade 4 events were exceptional. Grade 3/4 adverse events were observed in 41 patients (24.8%) when the everolimus dose was started at 5 mg and in 36 patients (42.9%) when the dose was started at 10 mg. In the AI + everolimus arm, 368 patients (98.4%) experienced at least one AE and 109 (29.1%) experienced a grade 3/4 AE. We observed grade 3 mucositis, elevated liver functional tests, and hypertriglyceridemia in 7.0%, 1.6%, and 1.3% of patients, respectively. One patient (0.3%) experienced grade 3 pneumonitis. Grade 4 events were exceptional. Similarly, this was observed in 61 patients (25.8%) when everolimus was started at 5 mg and in 48 patients (35.3%) at 10 mg.

The rate of dose reduction was similar in tamoxifen and AI patients, occurring in 34.9% of the tamoxifen everolimus group and 33.8% of the AI everolimus group. Overall, these results do not suggest a relevant difference in global toxicity profile according to the ET partner.

### Duration of treatment/discontinuation

**Figure 2:**
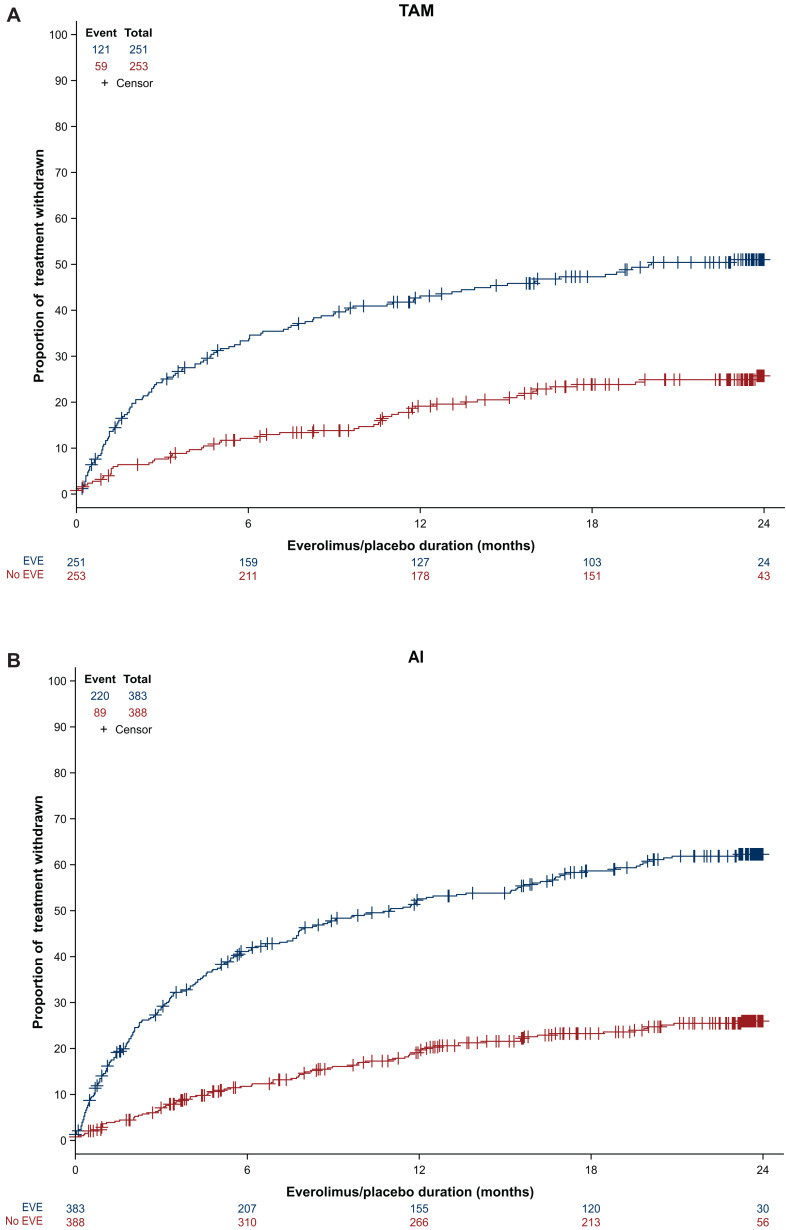
**Incidence of everolimus and placebo discontinuation according to endocrine therapy backbone.** Blue curves indicate the everolimus arm; red curves indicate the placebo arm. (A) Patients treated with tamoxifen. (B) Patients treated with an aromatase inhibitor.

Despite these data and most interestingly, early discontinuation of either everolimus or placebo was significantly less frequent in the tamoxifen arm than in the AI arm: 48.0% versus 56.9% (*P* = 0.028). Accordingly, the median duration of everolimus treatment was significantly longer in the tamoxifen group than in the AI group: 12.8 months (IQR 2.7-23.6 months) versus 7.7 months (IQR 1.9-22.6 months), *P* = 0.007. More specifically, as shown in Figure 2, the 2-year compliance rate with everolimus was ∼50% in the tamoxifen-treated patients compared with 35%-40% in the AI-treated patients. Compliance with placebo was similar in both endocrine regimens, with a cumulative 2-year compliance rate of ∼80%. Adherence to therapy is detailed in Table 3.

**Table 3:** Adherence to therapy in the UNIRAD trial, according to endocrine therapy

	Tamoxifen			Aromatase inhibitor		
	No EVE *n* = 253, *n* (%)	EVE *n* = 252, *n* (%)	All *N* = 505, *n* (%)	No EVE *n* = 388, *n* (%)	EVE *n* = 385, *n* (%)	All *N* = 773, *n* (%)
<30%	63 (24.9)	134 (53.4)	197 (39.1)	110 (28.4)	243 (63.1)	353 (45.7)
[30-50%]	41 (16.2)	53 (21.1)	94 (18.7)	71 (18.3)	61 (15.8)	132 (17.1)
[50-80%]	31 (12.3)	29 (11.6)	60 (11.9)	52 (13.4)	48 (12.5)	100 (12.9)
[80-90%]	15 (5.9)	8 (3.2)	23 (4.6)	27 (7.0)	11 (2.9)	38 (4.9)
[90-95%]	21 (8.3)	10 (4.0)	31 (6.2)	31 (8.0)	6 (1.6)	37 (4.8)
≥95%	82 (32.4)	17 (6.8)	99 (19.6)	97 (25.0)	16 (4.2)	113 (14.6)

Each row shows adherence as a percentage combining actual therapy duration and dose adjustment. For example, patients in the ‘<30%’ row have taken <30% of the theoretical cumulative dose. EVE, everolimus.

## Discussion

The addition of everolimus to ET is beneficial in patients with advanced HR-positive HER2-negative breast cancer receiving either with tamoxifen,^10^ fulvestrant^13^ or exemestane.^14^ On this basis, and with the aim of improving the outcomes for patients with high-risk early HR-positive HER2-negative breast cancer, we designed the UNIRAD trial to evaluate the addition of 2 years of everolimus to conventional adjuvant ET. Unfortunately, the trial did not meet its primary endpoint, likely due to insufficient drug exposure and/or limited activity in this specific clinical setting.^11^ Pre-specified subgroup analyses, however, suggested a better outcome with the addition of everolimus to tamoxifen. This subsequent *post hoc* analysis showed a trend in favor of everolimus in the subgroup of premenopausal patients treated with tamoxifen. In general, the most striking difference between patients treated with tamoxifen or an AI was the rate of discontinuation of everolimus (48.0% versus 56.9%, respectively) and the duration of exposure to everolimus.

Improving long-term outcomes with adjuvant therapies in patients with high-risk HR-positive, HER2-negative early breast cancer, whether premenopausal or not, remains an important unmet medical need. Recently, controversial results have challenged the role of adjuvant CDK4/6 inhibitors, as a means to limit long-term endocrine resistance by targeting the cell cycle machinery.^15^ Based on the results of the Monarch-E trial,^16^ abemaciclib was approved by the Food and Drug Administration and the European Medicines Agency for use in patients with very high-risk early HR-positive, HER2-negative breast cancer.^17^ Two adjuvant trials failed to demonstrate a benefit of palbociclib,^18^ while results from the NATALEE study evaluating ribociclib in patients with intermediate and high-risk early breast cancer showed a statistically significant improvement of invasive DFS.^19^ Adverse events associated with adjuvant targeted therapies are recognized as an important clinical issue. One of the most striking differences between the AI and the tamoxifen groups in the UNIRAD trial was the significantly higher rate of everolimus discontinuation in patients treated with an AI. Dose reductions or discontinuation have been observed in up to 20% of the patients in the metastatic setting, most commonly due to fatigue, rash, diarrhea or stomatitis.^10^^,^^13^^,^^14^ Of note, the trial was conducted before the generalization of adequate supportive care such as suggested by the SWISH trial.^20^ It is also recognized that compliance with ET is a major issue in the adjuvant setting, with adverse consequences for those patients who do not continue treatment.^21^ Notably, these issues of dose reduction/discontinuation and of compliance have also been encountered in recent adjuvant trials evaluating the potential benefit of CDK4/6 inhibitors.^18^^,^^22^ For instance, dose reductions were observed in 55% of patients receiving palbociclib in the PALLAS trial,^18^ in 41.2% of patients receiving abemaciclib in the Monarch-E trial,^15^ and in 21.9% of patients receiving ribociclib in the NATALEE trial.^23^ The dose discontinuation rates, however, were strikingly different (42.0% in PALLAS, 16.6% in Monarch-E, and 33.8% in NATALEE). This may help explain the lack of benefit with palbociclib and the impressive results obtained with abemaciclib or ribociclib, beyond the important differences in the populations of these two studies (Supplementary Table S4, available at https://doi.org/10.1016/j.esmoop.2025.105050). Taken together with our present results, these data may partially explain why no benefit was observed with everolimus, at least in patients treated with an AI with even a trend towards a detrimental effect of everolimus in premenopausal patients. Indeed, adherence to planned therapy was worse in the AI subgroup. Conversely, our data suggest that adjuvant everolimus may be beneficial in premenopausal patients treated with tamoxifen. In the SWOG1207 trial, the addition of 1 year of everolimus to ET was randomized with no benefit.^24^ Strikingly and in line with our results, the unplanned subgroup analyses of this trial also identified a potential specific benefit of adjuvant everolimus in premenopausal patients (hazard ratio 0.64, 95% CI 0.44-0.94), and in patients receiving tamoxifen (hazard ratio 0.78, 95% CI 0.53-1.15). Better compliance to treatment may be a first explanation (Figure 2), although no real difference in adverse events was observed between tamoxifen and AI-treated patients.

Another important hypothesis is that these patients with high-risk early breast cancer received suboptimal ET, and that everolimus compensated to some extent for this undertreatment. The vast majority of patients receiving tamoxifen in the UNIRAD study were premenopausal (65.7%), and it is noteworthy that OFS was proposed in only seven of them. At the time the study was designed and conducted, the final results of the SOFT trial, which definitively established OFS as the standard of care for premenopausal patients with high-risk early breast cancer, were not available.^25^ Tamoxifen alone was considered the standard of care for premenopausal women. Since amenorrhea is not a side-effect of everolimus,^26^ these results suggest that in this particular setting, everolimus *per se* may exert a significant clinical activity when added to tamoxifen. Other possible explanations may relate to specific biological interactions between tamoxifen and everolimus. We have found that taking tamoxifen in the evening may be more beneficial than taking it in the morning.^27^ It may also be hypothesized that everolimus limits activation of the eukaryotic translation initiation factor 4E (eIF4F) complex, which has been involved in resistance to tamoxifen.^28^^,^^29^ Indeed, several translational reports suggest that patients with high levels of p-4EBP1 (which triggers eIF4F activation) benefit more from everolimus therapy.^30^, ^31^, ^32^

This report has important limitations. UNIRAD is an underpowered study. The results are of borderline significance, and the present *post hoc* exploratory analyses are intended only to generate hypotheses that may help to understand the potential role of everolimus in patients with high-risk HR-positive HER2-negative early breast cancer. We did observe a slight imbalance in the risk factors in favor of the tamoxifen group, which could also have biased the results. Adherence to adjuvant ET is of paramount importance and may have been compromised by the combination to everolimus, especially in patients treated with an AI. We also have not been able to investigate potential underlying biological differences between premenopausal and postmenopausal patients. Only women participated in the UNIRAD study.

## Conclusions

Although current evidence does not support a substantial role for everolimus in the early breast cancer setting, the present data raise interesting hypotheses for the specific subset of premenopausal patients treated with adjuvant tamoxifen alone. The apparent adverse effect with AI remains a conundrum. In line with current recommendations for adjuvant ET, our results also suggest that tamoxifen alone, without additional OFS, may be suboptimal for high-risk premenopausal patients.
