# Supplementary material for: Differential benefit of adjuvant everolimus according to endocrine therapy backbone in the randomized UNIRAD trial
Source: ESMO Open. 2025 Apr 15;10(5):105050. doi: 10.1016/j.esmoop.2025.105050 (PMC12020834; doi:10.1016/j.esmoop.2025.105050)
Supplement: Supplementary Tables [file mmc1.docx]

| **Supplementary Table 1. Adverse events in the tamoxifen subgroup (cont’d)** | | | | | | | | |
| --- | --- | --- | --- | --- | --- | --- | --- | --- |
|  |  | Maximum grade | Placebo | | Everolimus | | Safety analysis set | |
|  |  |  | N=250 | | N=251 | | N=501 | |
| Not coded | Total | Total | 1 | (0.4%) | 2 | (0.8%) | 3 | (0.6%) |
|  |  | G1 | 1 | (0.4%) | 1 | (0.4%) | 2 | (0.4%) |
|  |  | G2 | 0 | (0.0%) | 1 | (0.4%) | 1 | (0.2%) |
|  | Not coded | Total | 1 | (0.4%) | 2 | (0.8%) | 3 | (0.6%) |
|  |  | G1 | 1 | (0.4%) | 1 | (0.4%) | 2 | (0.4%) |
|  |  | G2 | 0 | (0.0%) | 1 | (0.4%) | 1 | (0.2%) |
| BLOOD AND LYMPHATIC SYSTEM DISORDERS | Total | Total | 21 | (8.4%) | 47 | (18.7%) | 68 | (13.6%) |
|  |  | G1 | 20 | (8.0%) | 44 | (17.5%) | 64 | (12.8%) |
|  |  | G2 | 0 | (0.0%) | 3 | (1.2%) | 3 | (0.6%) |
|  |  | G3/4/5 | 1 | (0.4%) | 0 | (0.0%) | 1 | (0.2%) |
|  | ANEMIA | Total | 19 | (7.6%) | 45 | (17.9%) | 64 | (12.8%) |
|  |  | G1 | 19 | (7.6%) | 42 | (16.7%) | 61 | (12.2%) |
|  |  | G2 | 0 | (0.0%) | 3 | (1.2%) | 3 | (0.6%) |
|  | FEBRILE NEUTROPENIA | Total | 1 | (0.4%) | 0 | (0.0%) | 1 | (0.2%) |
|  |  | G3/4/5 | 1 | (0.4%) | 0 | (0.0%) | 1 | (0.2%) |
|  | LYMPH NODE PAIN | Total | 1 | (0.4%) | 0 | (0.0%) | 1 | (0.2%) |
|  |  | G1 | 1 | (0.4%) | 0 | (0.0%) | 1 | (0.2%) |
|  | LYMPHOCELE | Total | 0 | (0.0%) | 1 | (0.4%) | 1 | (0.2%) |
|  |  | G1 | 0 | (0.0%) | 1 | (0.4%) | 1 | (0.2%) |
|  | THROMBOTIC THROMBOCYTOPENIC PURPURA | Total | 1 | (0.4%) | 1 | (0.4%) | 2 | (0.4%) |
|  |  | G1 | 1 | (0.4%) | 1 | (0.4%) | 2 | (0.4%) |
| CARDIAC DISORDERS | Total | Total | 8 | (3.2%) | 7 | (2.8%) | 15 | (3.0%) |
|  |  | G1 | 6 | (2.4%) | 4 | (1.6%) | 10 | (2.0%) |
|  |  | G2 | 1 | (0.4%) | 2 | (0.8%) | 3 | (0.6%) |
|  |  | G3/4/5 | 1 | (0.4%) | 1 | (0.4%) | 2 | (0.4%) |
|  | ATRIAL FIBRILLATION | Total | 1 | (0.4%) | 0 | (0.0%) | 1 | (0.2%) |
|  |  | G1 | 1 | (0.4%) | 0 | (0.0%) | 1 | (0.2%) |
|  | CHEST PAIN - CARDIAC | Total | 2 | (0.8%) | 0 | (0.0%) | 2 | (0.4%) |
|  |  | G1 | 1 | (0.4%) | 0 | (0.0%) | 1 | (0.2%) |
|  |  | G2 | 1 | (0.4%) | 0 | (0.0%) | 1 | (0.2%) |
|  | EXTRASYSTOLE VENTRICULAR | Total | 0 | (0.0%) | 1 | (0.4%) | 1 | (0.2%) |
|  |  | G2 | 0 | (0.0%) | 1 | (0.4%) | 1 | (0.2%) |
|  | HEART FAILURE | Total | 0 | (0.0%) | 1 | (0.4%) | 1 | (0.2%) |
|  |  | G2 | 0 | (0.0%) | 1 | (0.4%) | 1 | (0.2%) |
|  | PALPITATIONS | Total | 4 | (1.6%) | 1 | (0.4%) | 5 | (1.0%) |
|  |  | G1 | 4 | (1.6%) | 1 | (0.4%) | 5 | (1.0%) |
|  | PERICARDITIS | Total | 1 | (0.4%) | 0 | (0.0%) | 1 | (0.2%) |
|  |  | G3/4/5 | 1 | (0.4%) | 0 | (0.0%) | 1 | (0.2%) |
|  | SINUS BRADYCARDIA | Total | 0 | (0.0%) | 1 | (0.4%) | 1 | (0.2%) |
|  |  | G1 | 0 | (0.0%) | 1 | (0.4%) | 1 | (0.2%) |
|  | SINUS TACHYCARDIA | Total | 0 | (0.0%) | 1 | (0.4%) | 1 | (0.2%) |
|  |  | G1 | 0 | (0.0%) | 1 | (0.4%) | 1 | (0.2%) |
|  | TACHYCARDIA | Total | 0 | (0.0%) | 2 | (0.8%) | 2 | (0.4%) |
|  |  | G1 | 0 | (0.0%) | 2 | (0.8%) | 2 | (0.4%) |
|  | VALVULAR HEART DISEASE NOS | Total | 0 | (0.0%) | 1 | (0.4%) | 1 | (0.2%) |
|  |  | G3/4/5 | 0 | (0.0%) | 1 | (0.4%) | 1 | (0.2%) |
| EAR AND LABYRINTH DISORDERS | Total | Total | 14 | (5.6%) | 7 | (2.8%) | 21 | (4.2%) |
|  |  | . | 0 | (0.0%) | 1 | (0.4%) | 1 | (0.2%) |
|  |  | G1 | 10 | (4.0%) | 4 | (1.6%) | 14 | (2.8%) |
|  |  | G2 | 4 | (1.6%) | 2 | (0.8%) | 6 | (1.2%) |
|  | CRYSTAL IN THE EAR | Total | 1 | (0.4%) | 0 | (0.0%) | 1 | (0.2%) |
|  |  | G1 | 1 | (0.4%) | 0 | (0.0%) | 1 | (0.2%) |
|  | EAR PAIN | Total | 0 | (0.0%) | 1 | (0.4%) | 1 | (0.2%) |
|  |  | G1 | 0 | (0.0%) | 1 | (0.4%) | 1 | (0.2%) |
|  | HEARING IMPAIRED | Total | 1 | (0.4%) | 0 | (0.0%) | 1 | (0.2%) |
|  |  | G1 | 1 | (0.4%) | 0 | (0.0%) | 1 | (0.2%) |
|  | MIDDLE EAR INFLAMMATION | Total | 0 | (0.0%) | 1 | (0.4%) | 1 | (0.2%) |
|  |  | G2 | 0 | (0.0%) | 1 | (0.4%) | 1 | (0.2%) |
|  | TINNITUS | Total | 1 | (0.4%) | 2 | (0.8%) | 3 | (0.6%) |
|  |  | G1 | 0 | (0.0%) | 1 | (0.4%) | 1 | (0.2%) |
|  |  | G2 | 1 | (0.4%) | 1 | (0.4%) | 2 | (0.4%) |
|  | VERTIGO | Total | 12 | (4.8%) | 3 | (1.2%) | 15 | (3.0%) |
|  |  | . | 0 | (0.0%) | 1 | (0.4%) | 1 | (0.2%) |
|  |  | G1 | 9 | (3.6%) | 2 | (0.8%) | 11 | (2.2%) |
|  |  | G2 | 3 | (1.2%) | 0 | (0.0%) | 3 | (0.6%) |
| ENDOCRINE DISORDERS | Total | Total | 4 | (1.6%) | 4 | (1.6%) | 8 | (1.6%) |
|  |  | G1 | 2 | (0.8%) | 2 | (0.8%) | 4 | (0.8%) |
|  |  | G2 | 2 | (0.8%) | 2 | (0.8%) | 4 | (0.8%) |
|  | HYPERTHYROIDISM | Total | 0 | (0.0%) | 2 | (0.8%) | 2 | (0.4%) |
|  |  | G1 | 0 | (0.0%) | 1 | (0.4%) | 1 | (0.2%) |
|  |  | G2 | 0 | (0.0%) | 1 | (0.4%) | 1 | (0.2%) |
|  | HYPOTHYROIDISM | Total | 4 | (1.6%) | 2 | (0.8%) | 6 | (1.2%) |
|  |  | G1 | 2 | (0.8%) | 1 | (0.4%) | 3 | (0.6%) |
|  |  | G2 | 2 | (0.8%) | 1 | (0.4%) | 3 | (0.6%) |
| EYE DISORDERS | Total | Total | 10 | (4.0%) | 22 | (8.8%) | 32 | (6.4%) |
|  |  | . | 1 | (0.4%) | 0 | (0.0%) | 1 | (0.2%) |
|  |  | G1 | 7 | (2.8%) | 20 | (8.0%) | 27 | (5.4%) |
|  |  | G2 | 2 | (0.8%) | 4 | (1.6%) | 6 | (1.2%) |
|  |  | G3/4/5 | 0 | (0.0%) | 1 | (0.4%) | 1 | (0.2%) |
|  | ACUITY REDUCED | Total | 0 | (0.0%) | 3 | (1.2%) | 3 | (0.6%) |
|  |  | G1 | 0 | (0.0%) | 3 | (1.2%) | 3 | (0.6%) |
|  | BINOCULAR EYE MOVEMENT DISORDER | Total | 1 | (0.4%) | 0 | (0.0%) | 1 | (0.2%) |
|  |  | G1 | 1 | (0.4%) | 0 | (0.0%) | 1 | (0.2%) |
|  | BLURRED VISION | Total | 1 | (0.4%) | 3 | (1.2%) | 4 | (0.8%) |
|  |  | G1 | 1 | (0.4%) | 2 | (0.8%) | 3 | (0.6%) |
|  |  | G2 | 0 | (0.0%) | 1 | (0.4%) | 1 | (0.2%) |
|  | CONJUNCTIVITIS | Total | 4 | (1.6%) | 10 | (4.0%) | 14 | (2.8%) |
|  |  | G1 | 2 | (0.8%) | 8 | (3.2%) | 10 | (2.0%) |
|  |  | G2 | 2 | (0.8%) | 2 | (0.8%) | 4 | (0.8%) |
|  | CORNEAL ULCER | Total | 0 | (0.0%) | 1 | (0.4%) | 1 | (0.2%) |
|  |  | G3/4/5 | 0 | (0.0%) | 1 | (0.4%) | 1 | (0.2%) |
|  | DRY EYE | Total | 2 | (0.8%) | 5 | (2.0%) | 7 | (1.4%) |
|  |  | G1 | 2 | (0.8%) | 5 | (2.0%) | 7 | (1.4%) |
|  | EYE PAIN | Total | 0 | (0.0%) | 2 | (0.8%) | 2 | (0.4%) |
|  |  | G1 | 0 | (0.0%) | 1 | (0.4%) | 1 | (0.2%) |
|  |  | G2 | 0 | (0.0%) | 1 | (0.4%) | 1 | (0.2%) |
|  | EYELID FUNCTION DISORDER | Total | 0 | (0.0%) | 1 | (0.4%) | 1 | (0.2%) |
|  |  | G1 | 0 | (0.0%) | 1 | (0.4%) | 1 | (0.2%) |
|  | FLOATERS | Total | 1 | (0.4%) | 0 | (0.0%) | 1 | (0.2%) |
|  |  | G1 | 1 | (0.4%) | 0 | (0.0%) | 1 | (0.2%) |
|  | SCLERAL DISORDER | Total | 1 | (0.4%) | 0 | (0.0%) | 1 | (0.2%) |
|  |  | . | 1 | (0.4%) | 0 | (0.0%) | 1 | (0.2%) |
|  | WATERING EYES | Total | 0 | (0.0%) | 2 | (0.8%) | 2 | (0.4%) |
|  |  | G1 | 0 | (0.0%) | 2 | (0.8%) | 2 | (0.4%) |
| GASTROINTESTINAL DISORDERS | Total | Total | 171 | (68.4%) | 215 | (85.7%) | 386 | (77.0%) |
|  |  | . | 1 | (0.4%) | 2 | (0.8%) | 3 | (0.6%) |
|  |  | G1 | 158 | (63.2%) | 165 | (65.7%) | 323 | (64.5%) |
|  |  | G2 | 30 | (12.0%) | 85 | (33.9%) | 115 | (23.0%) |
|  |  | G3/4/5 | 6 | (2.4%) | 24 | (9.6%) | 30 | (6.0%) |
|  | ABDOMINAL DISTENSION | Total | 1 | (0.4%) | 0 | (0.0%) | 1 | (0.2%) |
|  |  | G1 | 1 | (0.4%) | 0 | (0.0%) | 1 | (0.2%) |
|  | ABDOMINAL PAIN | Total | 15 | (6.0%) | 20 | (8.0%) | 35 | (7.0%) |
|  |  | . | 0 | (0.0%) | 1 | (0.4%) | 1 | (0.2%) |
|  |  | G1 | 14 | (5.6%) | 11 | (4.4%) | 25 | (5.0%) |
|  |  | G2 | 1 | (0.4%) | 8 | (3.2%) | 9 | (1.8%) |
|  | ANAL PAIN | Total | 0 | (0.0%) | 2 | (0.8%) | 2 | (0.4%) |
|  |  | G1 | 0 | (0.0%) | 2 | (0.8%) | 2 | (0.4%) |
|  | ASCITES | Total | 1 | (0.4%) | 0 | (0.0%) | 1 | (0.2%) |
|  |  | G1 | 1 | (0.4%) | 0 | (0.0%) | 1 | (0.2%) |
|  | BLOATING | Total | 4 | (1.6%) | 3 | (1.2%) | 7 | (1.4%) |
|  |  | G1 | 3 | (1.2%) | 3 | (1.2%) | 6 | (1.2%) |
|  |  | G2 | 1 | (0.4%) | 0 | (0.0%) | 1 | (0.2%) |
|  | CHEILITIS | Total | 0 | (0.0%) | 2 | (0.8%) | 2 | (0.4%) |
|  |  | G1 | 0 | (0.0%) | 1 | (0.4%) | 1 | (0.2%) |
|  |  | G2 | 0 | (0.0%) | 1 | (0.4%) | 1 | (0.2%) |
|  | COLITIS | Total | 1 | (0.4%) | 0 | (0.0%) | 1 | (0.2%) |
|  |  | G2 | 1 | (0.4%) | 0 | (0.0%) | 1 | (0.2%) |
|  | CONSTIPATION | Total | 26 | (10.4%) | 19 | (7.6%) | 45 | (9.0%) |
|  |  | G1 | 21 | (8.4%) | 16 | (6.4%) | 37 | (7.4%) |
|  |  | G2 | 5 | (2.0%) | 2 | (0.8%) | 7 | (1.4%) |
|  |  | G3/4/5 | 0 | (0.0%) | 1 | (0.4%) | 1 | (0.2%) |
|  | DENTAL CARIES | Total | 2 | (0.8%) | 1 | (0.4%) | 3 | (0.6%) |
|  |  | G1 | 1 | (0.4%) | 0 | (0.0%) | 1 | (0.2%) |
|  |  | G2 | 0 | (0.0%) | 1 | (0.4%) | 1 | (0.2%) |
|  |  | G3/4/5 | 1 | (0.4%) | 0 | (0.0%) | 1 | (0.2%) |
|  | DIARRHEA | Total | 50 | (20.0%) | 71 | (28.3%) | 121 | (24.2%) |
|  |  | G1 | 44 | (17.6%) | 54 | (21.5%) | 98 | (19.6%) |
|  |  | G2 | 4 | (1.6%) | 14 | (5.6%) | 18 | (3.6%) |
|  |  | G3/4/5 | 2 | (0.8%) | 3 | (1.2%) | 5 | (1.0%) |
|  | DIGESTICE DISORDER | Total | 1 | (0.4%) | 0 | (0.0%) | 1 | (0.2%) |
|  |  | . | 1 | (0.4%) | 0 | (0.0%) | 1 | (0.2%) |
|  | DIGESTIVE DISORDER | Total | 1 | (0.4%) | 0 | (0.0%) | 1 | (0.2%) |
|  |  | G1 | 1 | (0.4%) | 0 | (0.0%) | 1 | (0.2%) |
|  | DRY MOUTH | Total | 10 | (4.0%) | 17 | (6.8%) | 27 | (5.4%) |
|  |  | G1 | 9 | (3.6%) | 16 | (6.4%) | 25 | (5.0%) |
|  |  | G2 | 1 | (0.4%) | 1 | (0.4%) | 2 | (0.4%) |
|  | DYSPEPSIA | Total | 4 | (1.6%) | 4 | (1.6%) | 8 | (1.6%) |
|  |  | G1 | 3 | (1.2%) | 3 | (1.2%) | 6 | (1.2%) |
|  |  | G2 | 1 | (0.4%) | 1 | (0.4%) | 2 | (0.4%) |
|  | DYSPHAGIA | Total | 2 | (0.8%) | 5 | (2.0%) | 7 | (1.4%) |
|  |  | G1 | 1 | (0.4%) | 2 | (0.8%) | 3 | (0.6%) |
|  |  | G2 | 1 | (0.4%) | 2 | (0.8%) | 3 | (0.6%) |
|  |  | G3/4/5 | 0 | (0.0%) | 1 | (0.4%) | 1 | (0.2%) |
|  | ESOPHAGEAL OBSTRUCTION | Total | 1 | (0.4%) | 0 | (0.0%) | 1 | (0.2%) |
|  |  | G1 | 1 | (0.4%) | 0 | (0.0%) | 1 | (0.2%) |
|  | GASTRITIS | Total | 3 | (1.2%) | 2 | (0.8%) | 5 | (1.0%) |
|  |  | G1 | 2 | (0.8%) | 0 | (0.0%) | 2 | (0.4%) |
|  |  | G2 | 1 | (0.4%) | 2 | (0.8%) | 3 | (0.6%) |
|  | GASTROESOPHAGEAL REFLUX DISEASE | Total | 10 | (4.0%) | 10 | (4.0%) | 20 | (4.0%) |
|  |  | G1 | 6 | (2.4%) | 7 | (2.8%) | 13 | (2.6%) |
|  |  | G2 | 4 | (1.6%) | 3 | (1.2%) | 7 | (1.4%) |
|  | GASTROINTESTINAL PAIN | Total | 5 | (2.0%) | 3 | (1.2%) | 8 | (1.6%) |
|  |  | G1 | 4 | (1.6%) | 2 | (0.8%) | 6 | (1.2%) |
|  |  | G2 | 1 | (0.4%) | 0 | (0.0%) | 1 | (0.2%) |
|  |  | G3/4/5 | 0 | (0.0%) | 1 | (0.4%) | 1 | (0.2%) |
|  | GINGIVAL PAIN | Total | 3 | (1.2%) | 3 | (1.2%) | 6 | (1.2%) |
|  |  | G1 | 2 | (0.8%) | 3 | (1.2%) | 5 | (1.0%) |
|  |  | G2 | 1 | (0.4%) | 0 | (0.0%) | 1 | (0.2%) |
|  | GLOSSITIS | Total | 0 | (0.0%) | 1 | (0.4%) | 1 | (0.2%) |
|  |  | G1 | 0 | (0.0%) | 1 | (0.4%) | 1 | (0.2%) |
|  | HEMORRHOIDS | Total | 3 | (1.2%) | 8 | (3.2%) | 11 | (2.2%) |
|  |  | G1 | 2 | (0.8%) | 6 | (2.4%) | 8 | (1.6%) |
|  |  | G2 | 1 | (0.4%) | 2 | (0.8%) | 3 | (0.6%) |
|  | INDIGESTION | Total | 1 | (0.4%) | 0 | (0.0%) | 1 | (0.2%) |
|  |  | G2 | 1 | (0.4%) | 0 | (0.0%) | 1 | (0.2%) |
|  | LIP PAIN | Total | 1 | (0.4%) | 1 | (0.4%) | 2 | (0.4%) |
|  |  | G1 | 1 | (0.4%) | 1 | (0.4%) | 2 | (0.4%) |
|  | MUCOSITIS ORAL | Total | 103 | (41.2%) | 176 | (70.1%) | 279 | (55.7%) |
|  |  | G1 | 91 | (36.4%) | 99 | (39.4%) | 190 | (37.9%) |
|  |  | G2 | 11 | (4.4%) | 58 | (23.1%) | 69 | (13.8%) |
|  |  | G3/4/5 | 1 | (0.4%) | 19 | (7.6%) | 20 | (4.0%) |
|  | NAUSEA | Total | 52 | (20.8%) | 46 | (18.3%) | 98 | (19.6%) |
|  |  | . | 0 | (0.0%) | 1 | (0.4%) | 1 | (0.2%) |
|  |  | G1 | 47 | (18.8%) | 39 | (15.5%) | 86 | (17.2%) |
|  |  | G2 | 3 | (1.2%) | 5 | (2.0%) | 8 | (1.6%) |
|  |  | G3/4/5 | 2 | (0.8%) | 1 | (0.4%) | 3 | (0.6%) |
|  | ORAL PAIN | Total | 2 | (0.8%) | 1 | (0.4%) | 3 | (0.6%) |
|  |  | G1 | 1 | (0.4%) | 1 | (0.4%) | 2 | (0.4%) |
|  |  | G2 | 1 | (0.4%) | 0 | (0.0%) | 1 | (0.2%) |
|  | PANCREATITIS | Total | 0 | (0.0%) | 1 | (0.4%) | 1 | (0.2%) |
|  |  | G2 | 0 | (0.0%) | 1 | (0.4%) | 1 | (0.2%) |
|  | RECTAL HEMORRHAGE | Total | 1 | (0.4%) | 0 | (0.0%) | 1 | (0.2%) |
|  |  | G1 | 1 | (0.4%) | 0 | (0.0%) | 1 | (0.2%) |
|  | STOMACH PAIN | Total | 5 | (2.0%) | 2 | (0.8%) | 7 | (1.4%) |
|  |  | G1 | 4 | (1.6%) | 1 | (0.4%) | 5 | (1.0%) |
|  |  | G2 | 1 | (0.4%) | 1 | (0.4%) | 2 | (0.4%) |
|  | TOOTH DEVELOPMENT DISORDER | Total | 0 | (0.0%) | 1 | (0.4%) | 1 | (0.2%) |
|  |  | G1 | 0 | (0.0%) | 1 | (0.4%) | 1 | (0.2%) |
|  | TOOTHACHE | Total | 3 | (1.2%) | 0 | (0.0%) | 3 | (0.6%) |
|  |  | G1 | 2 | (0.8%) | 0 | (0.0%) | 2 | (0.4%) |
|  |  | G2 | 1 | (0.4%) | 0 | (0.0%) | 1 | (0.2%) |
|  | VOMITING | Total | 19 | (7.6%) | 21 | (8.4%) | 40 | (8.0%) |
|  |  | . | 0 | (0.0%) | 1 | (0.4%) | 1 | (0.2%) |
|  |  | G1 | 16 | (6.4%) | 18 | (7.2%) | 34 | (6.8%) |
|  |  | G2 | 3 | (1.2%) | 2 | (0.8%) | 5 | (1.0%) |
| GENERAL DISORDERS AND ADMINISTRATION SITE CONDITIONS | Total | Total | 147 | (58.8%) | 177 | (70.5%) | 324 | (64.7%) |
|  |  | . | 1 | (0.4%) | 1 | (0.4%) | 2 | (0.4%) |
|  |  | G1 | 113 | (45.2%) | 132 | (52.6%) | 245 | (48.9%) |
|  |  | G2 | 51 | (20.4%) | 63 | (25.1%) | 114 | (22.8%) |
|  |  | G3/4/5 | 7 | (2.8%) | 6 | (2.4%) | 13 | (2.6%) |
|  | CHILLS | Total | 1 | (0.4%) | 4 | (1.6%) | 5 | (1.0%) |
|  |  | G1 | 1 | (0.4%) | 4 | (1.6%) | 5 | (1.0%) |
|  | CYSTS, FAT NECROSIS & INFLAMMATION RELATED TO PREVIOUS SURGERY | Total | 0 | (0.0%) | 1 | (0.4%) | 1 | (0.2%) |
|  |  | G2 | 0 | (0.0%) | 1 | (0.4%) | 1 | (0.2%) |
|  | EDEMA FACE | Total | 0 | (0.0%) | 7 | (2.8%) | 7 | (1.4%) |
|  |  | G1 | 0 | (0.0%) | 7 | (2.8%) | 7 | (1.4%) |
|  | EDEMA LIMBS | Total | 8 | (3.2%) | 28 | (11.2%) | 36 | (7.2%) |
|  |  | G1 | 7 | (2.8%) | 19 | (7.6%) | 26 | (5.2%) |
|  |  | G2 | 1 | (0.4%) | 7 | (2.8%) | 8 | (1.6%) |
|  |  | G3/4/5 | 0 | (0.0%) | 2 | (0.8%) | 2 | (0.4%) |
|  | EDEMA TRUNK | Total | 1 | (0.4%) | 0 | (0.0%) | 1 | (0.2%) |
|  |  | G1 | 1 | (0.4%) | 0 | (0.0%) | 1 | (0.2%) |
|  | FATIGUE | Total | 115 | (46.0%) | 138 | (55.0%) | 253 | (50.5%) |
|  |  | G1 | 74 | (29.6%) | 90 | (35.9%) | 164 | (32.7%) |
|  |  | G2 | 37 | (14.8%) | 45 | (17.9%) | 82 | (16.4%) |
|  |  | G3/4/5 | 4 | (1.6%) | 3 | (1.2%) | 7 | (1.4%) |
|  | FEVER | Total | 14 | (5.6%) | 24 | (9.6%) | 38 | (7.6%) |
|  |  | G1 | 10 | (4.0%) | 19 | (7.6%) | 29 | (5.8%) |
|  |  | G2 | 4 | (1.6%) | 5 | (2.0%) | 9 | (1.8%) |
|  | FLU LIKE SYMPTOMS | Total | 17 | (6.8%) | 16 | (6.4%) | 33 | (6.6%) |
|  |  | . | 0 | (0.0%) | 1 | (0.4%) | 1 | (0.2%) |
|  |  | G1 | 8 | (3.2%) | 10 | (4.0%) | 18 | (3.6%) |
|  |  | G2 | 8 | (3.2%) | 5 | (2.0%) | 13 | (2.6%) |
|  |  | G3/4/5 | 1 | (0.4%) | 0 | (0.0%) | 1 | (0.2%) |
|  | IMPAIRED HEALING | Total | 2 | (0.8%) | 0 | (0.0%) | 2 | (0.4%) |
|  |  | . | 1 | (0.4%) | 0 | (0.0%) | 1 | (0.2%) |
|  |  | G1 | 1 | (0.4%) | 0 | (0.0%) | 1 | (0.2%) |
|  | IRRITABILITY | Total | 1 | (0.4%) | 2 | (0.8%) | 3 | (0.6%) |
|  |  | G1 | 1 | (0.4%) | 2 | (0.8%) | 3 | (0.6%) |
|  | LOCALIZED EDEMA | Total | 5 | (2.0%) | 7 | (2.8%) | 12 | (2.4%) |
|  |  | G1 | 3 | (1.2%) | 6 | (2.4%) | 9 | (1.8%) |
|  |  | G2 | 2 | (0.8%) | 1 | (0.4%) | 3 | (0.6%) |
|  | MALAISE | Total | 0 | (0.0%) | 2 | (0.8%) | 2 | (0.4%) |
|  |  | G1 | 0 | (0.0%) | 2 | (0.8%) | 2 | (0.4%) |
|  | NON-CARDIAC CHEST PAIN | Total | 2 | (0.8%) | 0 | (0.0%) | 2 | (0.4%) |
|  |  | G1 | 2 | (0.8%) | 0 | (0.0%) | 2 | (0.4%) |
|  | PAIN | Total | 42 | (16.8%) | 28 | (11.2%) | 70 | (14.0%) |
|  |  | G1 | 32 | (12.8%) | 18 | (7.2%) | 50 | (10.0%) |
|  |  | G2 | 8 | (3.2%) | 9 | (3.6%) | 17 | (3.4%) |
|  |  | G3/4/5 | 2 | (0.8%) | 1 | (0.4%) | 3 | (0.6%) |
| HEPATOBILIARY DISORDERS | Total | Total | 2 | (0.8%) | 4 | (1.6%) | 6 | (1.2%) |
|  |  | . | 0 | (0.0%) | 1 | (0.4%) | 1 | (0.2%) |
|  |  | G1 | 2 | (0.8%) | 2 | (0.8%) | 4 | (0.8%) |
|  |  | G3/4/5 | 0 | (0.0%) | 2 | (0.8%) | 2 | (0.4%) |
|  | BLOOD ALKALINE PHOSPHATASE DECREASED | Total | 1 | (0.4%) | 1 | (0.4%) | 2 | (0.4%) |
|  |  | G1 | 1 | (0.4%) | 1 | (0.4%) | 2 | (0.4%) |
|  | CHOLECYSTITIS | Total | 1 | (0.4%) | 0 | (0.0%) | 1 | (0.2%) |
|  |  | G1 | 1 | (0.4%) | 0 | (0.0%) | 1 | (0.2%) |
|  | ECHINOCOCCOSIS | Total | 0 | (0.0%) | 1 | (0.4%) | 1 | (0.2%) |
|  |  | G3/4/5 | 0 | (0.0%) | 1 | (0.4%) | 1 | (0.2%) |
|  | GALLBLADDER MICROLITHIASIS | Total | 0 | (0.0%) | 1 | (0.4%) | 1 | (0.2%) |
|  |  | . | 0 | (0.0%) | 1 | (0.4%) | 1 | (0.2%) |
|  | HEPATIC FAILURE | Total | 0 | (0.0%) | 2 | (0.8%) | 2 | (0.4%) |
|  |  | G1 | 0 | (0.0%) | 1 | (0.4%) | 1 | (0.2%) |
|  |  | G3/4/5 | 0 | (0.0%) | 1 | (0.4%) | 1 | (0.2%) |
| IMMUNE SYSTEM DISORDERS | Total | Total | 2 | (0.8%) | 2 | (0.8%) | 4 | (0.8%) |
|  |  | G1 | 2 | (0.8%) | 2 | (0.8%) | 4 | (0.8%) |
|  | ALLERGIC REACTION | Total | 2 | (0.8%) | 2 | (0.8%) | 4 | (0.8%) |
|  |  | G1 | 2 | (0.8%) | 2 | (0.8%) | 4 | (0.8%) |
| INFECTIONS AND INFESTATIONS | Total | Total | 91 | (36.4%) | 120 | (47.8%) | 211 | (42.1%) |
|  |  | . | 2 | (0.8%) | 2 | (0.8%) | 4 | (0.8%) |
|  |  | G1 | 47 | (18.8%) | 67 | (26.7%) | 114 | (22.8%) |
|  |  | G2 | 44 | (17.6%) | 67 | (26.7%) | 111 | (22.2%) |
|  |  | G3/4/5 | 8 | (3.2%) | 7 | (2.8%) | 15 | (3.0%) |
|  | ACTINOMYCOSIS | Total | 1 | (0.4%) | 0 | (0.0%) | 1 | (0.2%) |
|  |  | G2 | 1 | (0.4%) | 0 | (0.0%) | 1 | (0.2%) |
|  | BILIARY TRACT INFECTION | Total | 2 | (0.8%) | 0 | (0.0%) | 2 | (0.4%) |
|  |  | G3/4/5 | 2 | (0.8%) | 0 | (0.0%) | 2 | (0.4%) |
|  | BLADDER INFECTION | Total | 1 | (0.4%) | 0 | (0.0%) | 1 | (0.2%) |
|  |  | G2 | 1 | (0.4%) | 0 | (0.0%) | 1 | (0.2%) |
|  | BREAST INFECTION | Total | 1 | (0.4%) | 3 | (1.2%) | 4 | (0.8%) |
|  |  | G2 | 0 | (0.0%) | 2 | (0.8%) | 2 | (0.4%) |
|  |  | G3/4/5 | 1 | (0.4%) | 1 | (0.4%) | 2 | (0.4%) |
|  | BRONCHIAL INFECTION | Total | 13 | (5.2%) | 21 | (8.4%) | 34 | (6.8%) |
|  |  | G1 | 6 | (2.4%) | 6 | (2.4%) | 12 | (2.4%) |
|  |  | G2 | 7 | (2.8%) | 15 | (6.0%) | 22 | (4.4%) |
|  | CANDIDA INFECTION | Total | 0 | (0.0%) | 1 | (0.4%) | 1 | (0.2%) |
|  |  | G1 | 0 | (0.0%) | 1 | (0.4%) | 1 | (0.2%) |
|  | COVID19 | Total | 0 | (0.0%) | 1 | (0.4%) | 1 | (0.2%) |
|  |  | G1 | 0 | (0.0%) | 1 | (0.4%) | 1 | (0.2%) |
|  | ENTEROCOLITIS INFECTIOUS | Total | 3 | (1.2%) | 7 | (2.8%) | 10 | (2.0%) |
|  |  | G1 | 1 | (0.4%) | 3 | (1.2%) | 4 | (0.8%) |
|  |  | G2 | 2 | (0.8%) | 4 | (1.6%) | 6 | (1.2%) |
|  | EYE INFECTION | Total | 0 | (0.0%) | 2 | (0.8%) | 2 | (0.4%) |
|  |  | G1 | 0 | (0.0%) | 2 | (0.8%) | 2 | (0.4%) |
|  | GALLBLADDER INFECTION | Total | 1 | (0.4%) | 1 | (0.4%) | 2 | (0.4%) |
|  |  | G3/4/5 | 1 | (0.4%) | 1 | (0.4%) | 2 | (0.4%) |
|  | GUM INFECTION | Total | 0 | (0.0%) | 1 | (0.4%) | 1 | (0.2%) |
|  |  | G2 | 0 | (0.0%) | 1 | (0.4%) | 1 | (0.2%) |
|  | HALITOSIS | Total | 0 | (0.0%) | 1 | (0.4%) | 1 | (0.2%) |
|  |  | G1 | 0 | (0.0%) | 1 | (0.4%) | 1 | (0.2%) |
|  | HERPES | Total | 0 | (0.0%) | 1 | (0.4%) | 1 | (0.2%) |
|  |  | G2 | 0 | (0.0%) | 1 | (0.4%) | 1 | (0.2%) |
|  | INFLAMMATORY BOWEL DISEASE | Total | 1 | (0.4%) | 0 | (0.0%) | 1 | (0.2%) |
|  |  | G3/4/5 | 1 | (0.4%) | 0 | (0.0%) | 1 | (0.2%) |
|  | JOINT INFECTION | Total | 0 | (0.0%) | 3 | (1.2%) | 3 | (0.6%) |
|  |  | G1 | 0 | (0.0%) | 2 | (0.8%) | 2 | (0.4%) |
|  |  | G3/4/5 | 0 | (0.0%) | 1 | (0.4%) | 1 | (0.2%) |
|  | KIDNEY INFECTION | Total | 2 | (0.8%) | 2 | (0.8%) | 4 | (0.8%) |
|  |  | G1 | 0 | (0.0%) | 1 | (0.4%) | 1 | (0.2%) |
|  |  | G2 | 1 | (0.4%) | 1 | (0.4%) | 2 | (0.4%) |
|  |  | G3/4/5 | 1 | (0.4%) | 0 | (0.0%) | 1 | (0.2%) |
|  | LARYNGITIS | Total | 2 | (0.8%) | 2 | (0.8%) | 4 | (0.8%) |
|  |  | G1 | 2 | (0.8%) | 0 | (0.0%) | 2 | (0.4%) |
|  |  | G2 | 0 | (0.0%) | 2 | (0.8%) | 2 | (0.4%) |
|  | LIP INFECTION | Total | 7 | (2.8%) | 8 | (3.2%) | 15 | (3.0%) |
|  |  | G1 | 3 | (1.2%) | 5 | (2.0%) | 8 | (1.6%) |
|  |  | G2 | 4 | (1.6%) | 2 | (0.8%) | 6 | (1.2%) |
|  |  | G3/4/5 | 0 | (0.0%) | 1 | (0.4%) | 1 | (0.2%) |
|  | LOCALIZED | Total | 1 | (0.4%) | 1 | (0.4%) | 2 | (0.4%) |
|  |  | G1 | 1 | (0.4%) | 0 | (0.0%) | 1 | (0.2%) |
|  |  | G3/4/5 | 0 | (0.0%) | 1 | (0.4%) | 1 | (0.2%) |
|  | LUNG INFECTION | Total | 3 | (1.2%) | 4 | (1.6%) | 7 | (1.4%) |
|  |  | G1 | 1 | (0.4%) | 0 | (0.0%) | 1 | (0.2%) |
|  |  | G2 | 2 | (0.8%) | 3 | (1.2%) | 5 | (1.0%) |
|  |  | G3/4/5 | 0 | (0.0%) | 1 | (0.4%) | 1 | (0.2%) |
|  | LYMPH GLAND INFECTION | Total | 2 | (0.8%) | 2 | (0.8%) | 4 | (0.8%) |
|  |  | G1 | 2 | (0.8%) | 1 | (0.4%) | 3 | (0.6%) |
|  |  | G2 | 0 | (0.0%) | 1 | (0.4%) | 1 | (0.2%) |
|  | MUCOSAL INFECTION | Total | 1 | (0.4%) | 8 | (3.2%) | 9 | (1.8%) |
|  |  | . | 0 | (0.0%) | 1 | (0.4%) | 1 | (0.2%) |
|  |  | G1 | 1 | (0.4%) | 6 | (2.4%) | 7 | (1.4%) |
|  |  | G2 | 0 | (0.0%) | 1 | (0.4%) | 1 | (0.2%) |
|  | MYCOSIS | Total | 3 | (1.2%) | 1 | (0.4%) | 4 | (0.8%) |
|  |  | G1 | 2 | (0.8%) | 1 | (0.4%) | 3 | (0.6%) |
|  |  | G2 | 1 | (0.4%) | 0 | (0.0%) | 1 | (0.2%) |
|  | NAIL INFECTION | Total | 1 | (0.4%) | 2 | (0.8%) | 3 | (0.6%) |
|  |  | G1 | 1 | (0.4%) | 1 | (0.4%) | 2 | (0.4%) |
|  |  | G2 | 0 | (0.0%) | 1 | (0.4%) | 1 | (0.2%) |
|  | OTITIS EXTERNA | Total | 1 | (0.4%) | 4 | (1.6%) | 5 | (1.0%) |
|  |  | G1 | 0 | (0.0%) | 1 | (0.4%) | 1 | (0.2%) |
|  |  | G2 | 1 | (0.4%) | 3 | (1.2%) | 4 | (0.8%) |
|  | OTITIS MEDIA | Total | 1 | (0.4%) | 0 | (0.0%) | 1 | (0.2%) |
|  |  | G2 | 1 | (0.4%) | 0 | (0.0%) | 1 | (0.2%) |
|  | PAPULOPUSTULAR RASH | Total | 0 | (0.0%) | 1 | (0.4%) | 1 | (0.2%) |
|  |  | G1 | 0 | (0.0%) | 1 | (0.4%) | 1 | (0.2%) |
|  | PARONYCHIA | Total | 0 | (0.0%) | 1 | (0.4%) | 1 | (0.2%) |
|  |  | G1 | 0 | (0.0%) | 1 | (0.4%) | 1 | (0.2%) |
|  | PERITONEAL INFECTION | Total | 0 | (0.0%) | 1 | (0.4%) | 1 | (0.2%) |
|  |  | G1 | 0 | (0.0%) | 1 | (0.4%) | 1 | (0.2%) |
|  | PHARYNGITIS | Total | 11 | (4.4%) | 7 | (2.8%) | 18 | (3.6%) |
|  |  | G1 | 9 | (3.6%) | 1 | (0.4%) | 10 | (2.0%) |
|  |  | G2 | 2 | (0.8%) | 6 | (2.4%) | 8 | (1.6%) |
|  | PLEURAL INFECTION | Total | 1 | (0.4%) | 0 | (0.0%) | 1 | (0.2%) |
|  |  | . | 1 | (0.4%) | 0 | (0.0%) | 1 | (0.2%) |
|  | RASH PUSTULAR | Total | 0 | (0.0%) | 4 | (1.6%) | 4 | (0.8%) |
|  |  | G1 | 0 | (0.0%) | 1 | (0.4%) | 1 | (0.2%) |
|  |  | G2 | 0 | (0.0%) | 3 | (1.2%) | 3 | (0.6%) |
|  | RHINITIS | Total | 2 | (0.8%) | 2 | (0.8%) | 4 | (0.8%) |
|  |  | G1 | 1 | (0.4%) | 1 | (0.4%) | 2 | (0.4%) |
|  |  | G2 | 1 | (0.4%) | 1 | (0.4%) | 2 | (0.4%) |
|  | RHINITIS INFECTIVE | Total | 7 | (2.8%) | 22 | (8.8%) | 29 | (5.8%) |
|  |  | . | 1 | (0.4%) | 0 | (0.0%) | 1 | (0.2%) |
|  |  | G1 | 5 | (2.0%) | 14 | (5.6%) | 19 | (3.8%) |
|  |  | G2 | 1 | (0.4%) | 8 | (3.2%) | 9 | (1.8%) |
|  | RHINOPHARYNGITIS | Total | 3 | (1.2%) | 2 | (0.8%) | 5 | (1.0%) |
|  |  | G1 | 2 | (0.8%) | 1 | (0.4%) | 3 | (0.6%) |
|  |  | G2 | 1 | (0.4%) | 1 | (0.4%) | 2 | (0.4%) |
|  | SCLERODERMA | Total | 0 | (0.0%) | 1 | (0.4%) | 1 | (0.2%) |
|  |  | G1 | 0 | (0.0%) | 1 | (0.4%) | 1 | (0.2%) |
|  | SINUSITIS | Total | 2 | (0.8%) | 7 | (2.8%) | 9 | (1.8%) |
|  |  | G1 | 2 | (0.8%) | 3 | (1.2%) | 5 | (1.0%) |
|  |  | G2 | 0 | (0.0%) | 4 | (1.6%) | 4 | (0.8%) |
|  | SKIN INFECTION | Total | 17 | (6.8%) | 22 | (8.8%) | 39 | (7.8%) |
|  |  | . | 0 | (0.0%) | 1 | (0.4%) | 1 | (0.2%) |
|  |  | G1 | 12 | (4.8%) | 14 | (5.6%) | 26 | (5.2%) |
|  |  | G2 | 4 | (1.6%) | 7 | (2.8%) | 11 | (2.2%) |
|  |  | G3/4/5 | 1 | (0.4%) | 0 | (0.0%) | 1 | (0.2%) |
|  | SUPERINFECTION | Total | 0 | (0.0%) | 1 | (0.4%) | 1 | (0.2%) |
|  |  | G1 | 0 | (0.0%) | 1 | (0.4%) | 1 | (0.2%) |
|  | TOOTH INFECTION | Total | 5 | (2.0%) | 13 | (5.2%) | 18 | (3.6%) |
|  |  | G1 | 0 | (0.0%) | 1 | (0.4%) | 1 | (0.2%) |
|  |  | G2 | 5 | (2.0%) | 12 | (4.8%) | 17 | (3.4%) |
|  | TRACHEITIS | Total | 1 | (0.4%) | 0 | (0.0%) | 1 | (0.2%) |
|  |  | G2 | 1 | (0.4%) | 0 | (0.0%) | 1 | (0.2%) |
|  | UPPER RESPIRATORY INFECTION | Total | 4 | (1.6%) | 4 | (1.6%) | 8 | (1.6%) |
|  |  | G1 | 3 | (1.2%) | 3 | (1.2%) | 6 | (1.2%) |
|  |  | G2 | 1 | (0.4%) | 1 | (0.4%) | 2 | (0.4%) |
|  | URINARY TRACT INFECTION | Total | 13 | (5.2%) | 20 | (8.0%) | 33 | (6.6%) |
|  |  | G1 | 1 | (0.4%) | 8 | (3.2%) | 9 | (1.8%) |
|  |  | G2 | 11 | (4.4%) | 11 | (4.4%) | 22 | (4.4%) |
|  |  | G3/4/5 | 1 | (0.4%) | 1 | (0.4%) | 2 | (0.4%) |
|  | UTERINE INFECTION | Total | 0 | (0.0%) | 1 | (0.4%) | 1 | (0.2%) |
|  |  | G2 | 0 | (0.0%) | 1 | (0.4%) | 1 | (0.2%) |
|  | VAGINAL INFECTION | Total | 6 | (2.4%) | 11 | (4.4%) | 17 | (3.4%) |
|  |  | G1 | 3 | (1.2%) | 5 | (2.0%) | 8 | (1.6%) |
|  |  | G2 | 3 | (1.2%) | 6 | (2.4%) | 9 | (1.8%) |
|  | VULVAL INFECTION | Total | 2 | (0.8%) | 3 | (1.2%) | 5 | (1.0%) |
|  |  | G1 | 1 | (0.4%) | 2 | (0.8%) | 3 | (0.6%) |
|  |  | G2 | 1 | (0.4%) | 1 | (0.4%) | 2 | (0.4%) |
|  | WOUND INFECTION | Total | 1 | (0.4%) | 2 | (0.8%) | 3 | (0.6%) |
|  |  | G2 | 1 | (0.4%) | 2 | (0.8%) | 3 | (0.6%) |
| INJURY, POISONING AND PROCEDURAL COMPLICATIONS | Total | Total | 7 | (2.8%) | 7 | (2.8%) | 14 | (2.8%) |
|  |  | G1 | 2 | (0.8%) | 3 | (1.2%) | 5 | (1.0%) |
|  |  | G2 | 5 | (2.0%) | 4 | (1.6%) | 9 | (1.8%) |
|  | ANKLE FRACTURE | Total | 1 | (0.4%) | 0 | (0.0%) | 1 | (0.2%) |
|  |  | G2 | 1 | (0.4%) | 0 | (0.0%) | 1 | (0.2%) |
|  | BRUISING | Total | 3 | (1.2%) | 0 | (0.0%) | 3 | (0.6%) |
|  |  | G1 | 2 | (0.8%) | 0 | (0.0%) | 2 | (0.4%) |
|  |  | G2 | 1 | (0.4%) | 0 | (0.0%) | 1 | (0.2%) |
|  | FRACTURE | Total | 4 | (1.6%) | 6 | (2.4%) | 10 | (2.0%) |
|  |  | G1 | 0 | (0.0%) | 3 | (1.2%) | 3 | (0.6%) |
|  |  | G2 | 4 | (1.6%) | 3 | (1.2%) | 7 | (1.4%) |
|  | WRIST FRACTURE | Total | 0 | (0.0%) | 1 | (0.4%) | 1 | (0.2%) |
|  |  | G2 | 0 | (0.0%) | 1 | (0.4%) | 1 | (0.2%) |
| INVESTIGATIONS | Total | Total | 139 | (55.6%) | 159 | (63.3%) | 298 | (59.5%) |
|  |  | . | 3 | (1.2%) | 1 | (0.4%) | 4 | (0.8%) |
|  |  | G1 | 133 | (53.2%) | 143 | (57.0%) | 276 | (55.1%) |
|  |  | G2 | 20 | (8.0%) | 50 | (19.9%) | 70 | (14.0%) |
|  |  | G3/4/5 | 7 | (2.8%) | 16 | (6.4%) | 23 | (4.6%) |
|  | ALANINE AMINOTRANSFERASE DECREASED | Total | 1 | (0.4%) | 0 | (0.0%) | 1 | (0.2%) |
|  |  | G1 | 1 | (0.4%) | 0 | (0.0%) | 1 | (0.2%) |
|  | ALKALINE PHOSPHATASE INCREASED | Total | 5 | (2.0%) | 11 | (4.4%) | 16 | (3.2%) |
|  |  | G1 | 5 | (2.0%) | 10 | (4.0%) | 15 | (3.0%) |
|  |  | G3/4/5 | 0 | (0.0%) | 1 | (0.4%) | 1 | (0.2%) |
|  | BLOOD ALKALINE PHOSPHATASE LOW | Total | 1 | (0.4%) | 0 | (0.0%) | 1 | (0.2%) |
|  |  | G1 | 1 | (0.4%) | 0 | (0.0%) | 1 | (0.2%) |
|  | BLOOD ANTIDIURETIC HORMONE ABNORMAL | Total | 0 | (0.0%) | 1 | (0.4%) | 1 | (0.2%) |
|  |  | G1 | 0 | (0.0%) | 1 | (0.4%) | 1 | (0.2%) |
|  | BLOOD BILIRUBIN DECREASED | Total | 3 | (1.2%) | 5 | (2.0%) | 8 | (1.6%) |
|  |  | . | 1 | (0.4%) | 0 | (0.0%) | 1 | (0.2%) |
|  |  | G1 | 2 | (0.8%) | 5 | (2.0%) | 7 | (1.4%) |
|  | BLOOD BILIRUBIN INCREASED | Total | 9 | (3.6%) | 1 | (0.4%) | 10 | (2.0%) |
|  |  | G1 | 9 | (3.6%) | 1 | (0.4%) | 10 | (2.0%) |
|  | BLOOD CHLORIDE DECREASED | Total | 0 | (0.0%) | 1 | (0.4%) | 1 | (0.2%) |
|  |  | G1 | 0 | (0.0%) | 1 | (0.4%) | 1 | (0.2%) |
|  | BLOOD EOSINOPHILS INCREASED | Total | 1 | (0.4%) | 0 | (0.0%) | 1 | (0.2%) |
|  |  | G1 | 1 | (0.4%) | 0 | (0.0%) | 1 | (0.2%) |
|  | BLOOD MONOCYTES DECREASED | Total | 1 | (0.4%) | 0 | (0.0%) | 1 | (0.2%) |
|  |  | G1 | 1 | (0.4%) | 0 | (0.0%) | 1 | (0.2%) |
|  | BLOOD MONOCYTES INCREASED | Total | 1 | (0.4%) | 0 | (0.0%) | 1 | (0.2%) |
|  |  | G1 | 1 | (0.4%) | 0 | (0.0%) | 1 | (0.2%) |
|  | BLOOD PHOSPHORUS INCREASED | Total | 3 | (1.2%) | 5 | (2.0%) | 8 | (1.6%) |
|  |  | . | 1 | (0.4%) | 0 | (0.0%) | 1 | (0.2%) |
|  |  | G1 | 2 | (0.8%) | 4 | (1.6%) | 6 | (1.2%) |
|  |  | G2 | 0 | (0.0%) | 1 | (0.4%) | 1 | (0.2%) |
|  | CD4 LYMPHOCYTES DECREASED | Total | 1 | (0.4%) | 0 | (0.0%) | 1 | (0.2%) |
|  |  | G1 | 1 | (0.4%) | 0 | (0.0%) | 1 | (0.2%) |
|  | CHLORIDE DECREASED | Total | 0 | (0.0%) | 1 | (0.4%) | 1 | (0.2%) |
|  |  | G1 | 0 | (0.0%) | 1 | (0.4%) | 1 | (0.2%) |
|  | CHLORIDE INCREASED | Total | 1 | (0.4%) | 5 | (2.0%) | 6 | (1.2%) |
|  |  | . | 0 | (0.0%) | 1 | (0.4%) | 1 | (0.2%) |
|  |  | G1 | 1 | (0.4%) | 4 | (1.6%) | 5 | (1.0%) |
|  | CHOLESTEROL HIGH | Total | 58 | (23.2%) | 88 | (35.1%) | 146 | (29.1%) |
|  |  | . | 1 | (0.4%) | 1 | (0.4%) | 2 | (0.4%) |
|  |  | G1 | 56 | (22.4%) | 77 | (30.7%) | 133 | (26.5%) |
|  |  | G2 | 1 | (0.4%) | 7 | (2.8%) | 8 | (1.6%) |
|  |  | G3/4/5 | 0 | (0.0%) | 3 | (1.2%) | 3 | (0.6%) |
|  | CHOLESTEROL LOW | Total | 0 | (0.0%) | 1 | (0.4%) | 1 | (0.2%) |
|  |  | G1 | 0 | (0.0%) | 1 | (0.4%) | 1 | (0.2%) |
|  | CREATININE DECREASED | Total | 1 | (0.4%) | 4 | (1.6%) | 5 | (1.0%) |
|  |  | G1 | 1 | (0.4%) | 4 | (1.6%) | 5 | (1.0%) |
|  | CREATININE INCREASED | Total | 9 | (3.6%) | 4 | (1.6%) | 13 | (2.6%) |
|  |  | G1 | 9 | (3.6%) | 4 | (1.6%) | 13 | (2.6%) |
|  | DISCOMFORT | Total | 1 | (0.4%) | 0 | (0.0%) | 1 | (0.2%) |
|  |  | G1 | 1 | (0.4%) | 0 | (0.0%) | 1 | (0.2%) |
|  | HDL CHOLESTEROL DECREASE | Total | 4 | (1.6%) | 1 | (0.4%) | 5 | (1.0%) |
|  |  | G1 | 4 | (1.6%) | 1 | (0.4%) | 5 | (1.0%) |
|  | HEMOGLOBIN INCREASED | Total | 1 | (0.4%) | 0 | (0.0%) | 1 | (0.2%) |
|  |  | G1 | 1 | (0.4%) | 0 | (0.0%) | 1 | (0.2%) |
|  | HEPATIC ALAT/ASAT/GGT INCREASE | Total | 51 | (20.4%) | 68 | (27.1%) | 119 | (23.8%) |
|  |  | G1 | 41 | (16.4%) | 53 | (21.1%) | 94 | (18.8%) |
|  |  | G2 | 7 | (2.8%) | 7 | (2.8%) | 14 | (2.8%) |
|  |  | G3/4/5 | 3 | (1.2%) | 8 | (3.2%) | 11 | (2.2%) |
|  | HIGH UREA | Total | 3 | (1.2%) | 1 | (0.4%) | 4 | (0.8%) |
|  |  | G1 | 3 | (1.2%) | 1 | (0.4%) | 4 | (0.8%) |
|  | LDH INCREASE | Total | 1 | (0.4%) | 0 | (0.0%) | 1 | (0.2%) |
|  |  | G1 | 1 | (0.4%) | 0 | (0.0%) | 1 | (0.2%) |
|  | LYMPHANGITIS | Total | 1 | (0.4%) | 0 | (0.0%) | 1 | (0.2%) |
|  |  | G1 | 1 | (0.4%) | 0 | (0.0%) | 1 | (0.2%) |
|  | LYMPHOCYTE COUNT DECREASED | Total | 38 | (15.2%) | 50 | (19.9%) | 88 | (17.6%) |
|  |  | G1 | 32 | (12.8%) | 38 | (15.1%) | 70 | (14.0%) |
|  |  | G2 | 4 | (1.6%) | 9 | (3.6%) | 13 | (2.6%) |
|  |  | G3/4/5 | 2 | (0.8%) | 3 | (1.2%) | 5 | (1.0%) |
|  | LYMPHOCYTE COUNT INCREASED | Total | 1 | (0.4%) | 1 | (0.4%) | 2 | (0.4%) |
|  |  | G1 | 1 | (0.4%) | 1 | (0.4%) | 2 | (0.4%) |
|  | NEUTROPHIL COUNT DECREASED | Total | 22 | (8.8%) | 52 | (20.7%) | 74 | (14.8%) |
|  |  | G1 | 15 | (6.0%) | 24 | (9.6%) | 39 | (7.8%) |
|  |  | G2 | 5 | (2.0%) | 26 | (10.4%) | 31 | (6.2%) |
|  |  | G3/4/5 | 2 | (0.8%) | 2 | (0.8%) | 4 | (0.8%) |
|  | PHOSPHORE DECREASED | Total | 1 | (0.4%) | 2 | (0.8%) | 3 | (0.6%) |
|  |  | G1 | 1 | (0.4%) | 2 | (0.8%) | 3 | (0.6%) |
|  | PLATELET COUNT DECREASED | Total | 7 | (2.8%) | 26 | (10.4%) | 33 | (6.6%) |
|  |  | G1 | 7 | (2.8%) | 25 | (10.0%) | 32 | (6.4%) |
|  |  | G2 | 0 | (0.0%) | 1 | (0.4%) | 1 | (0.2%) |
|  | PLATELET COUNT INCREASED | Total | 1 | (0.4%) | 1 | (0.4%) | 2 | (0.4%) |
|  |  | G1 | 1 | (0.4%) | 1 | (0.4%) | 2 | (0.4%) |
|  | WEIGHT GAIN | Total | 9 | (3.6%) | 3 | (1.2%) | 12 | (2.4%) |
|  |  | G1 | 7 | (2.8%) | 2 | (0.8%) | 9 | (1.8%) |
|  |  | G2 | 2 | (0.8%) | 0 | (0.0%) | 2 | (0.4%) |
|  |  | G3/4/5 | 0 | (0.0%) | 1 | (0.4%) | 1 | (0.2%) |
|  | WEIGHT LOSS | Total | 8 | (3.2%) | 15 | (6.0%) | 23 | (4.6%) |
|  |  | G1 | 6 | (2.4%) | 13 | (5.2%) | 19 | (3.8%) |
|  |  | G2 | 2 | (0.8%) | 2 | (0.8%) | 4 | (0.8%) |
|  | WHITE BLOOD CELL DECREASED | Total | 14 | (5.6%) | 22 | (8.8%) | 36 | (7.2%) |
|  |  | G1 | 14 | (5.6%) | 16 | (6.4%) | 30 | (6.0%) |
|  |  | G2 | 0 | (0.0%) | 6 | (2.4%) | 6 | (1.2%) |
| METABOLISM AND NUTRITION DISORDERS | Total | Total | 96 | (38.4%) | 127 | (50.6%) | 223 | (44.5%) |
|  |  | G1 | 84 | (33.6%) | 105 | (41.8%) | 189 | (37.7%) |
|  |  | G2 | 17 | (6.8%) | 26 | (10.4%) | 43 | (8.6%) |
|  |  | G3/4/5 | 4 | (1.6%) | 19 | (7.6%) | 23 | (4.6%) |
|  | ANOREXIA | Total | 15 | (6.0%) | 17 | (6.8%) | 32 | (6.4%) |
|  |  | G1 | 9 | (3.6%) | 14 | (5.6%) | 23 | (4.6%) |
|  |  | G2 | 5 | (2.0%) | 3 | (1.2%) | 8 | (1.6%) |
|  |  | G3/4/5 | 1 | (0.4%) | 0 | (0.0%) | 1 | (0.2%) |
|  | HYPERCALCEMIA | Total | 2 | (0.8%) | 3 | (1.2%) | 5 | (1.0%) |
|  |  | G1 | 2 | (0.8%) | 2 | (0.8%) | 4 | (0.8%) |
|  |  | G3/4/5 | 0 | (0.0%) | 1 | (0.4%) | 1 | (0.2%) |
|  | HYPERGLYCEMIA | Total | 27 | (10.8%) | 39 | (15.5%) | 66 | (13.2%) |
|  |  | G1 | 25 | (10.0%) | 33 | (13.1%) | 58 | (11.6%) |
|  |  | G2 | 1 | (0.4%) | 3 | (1.2%) | 4 | (0.8%) |
|  |  | G3/4/5 | 1 | (0.4%) | 3 | (1.2%) | 4 | (0.8%) |
|  | HYPERKALEMIA | Total | 20 | (8.0%) | 8 | (3.2%) | 28 | (5.6%) |
|  |  | G1 | 20 | (8.0%) | 8 | (3.2%) | 28 | (5.6%) |
|  | HYPERNATREMIA | Total | 1 | (0.4%) | 2 | (0.8%) | 3 | (0.6%) |
|  |  | G1 | 1 | (0.4%) | 2 | (0.8%) | 3 | (0.6%) |
|  | HYPERPHOSPHATEMIA | Total | 0 | (0.0%) | 1 | (0.4%) | 1 | (0.2%) |
|  |  | G1 | 0 | (0.0%) | 1 | (0.4%) | 1 | (0.2%) |
|  | HYPERTRIGLYCERIDEMIA | Total | 45 | (18.0%) | 87 | (34.7%) | 132 | (26.3%) |
|  |  | G1 | 35 | (14.0%) | 55 | (21.9%) | 90 | (18.0%) |
|  |  | G2 | 9 | (3.6%) | 18 | (7.2%) | 27 | (5.4%) |
|  |  | G3/4/5 | 1 | (0.4%) | 14 | (5.6%) | 15 | (3.0%) |
|  | HYPERURICEMIA | Total | 2 | (0.8%) | 3 | (1.2%) | 5 | (1.0%) |
|  |  | G1 | 2 | (0.8%) | 3 | (1.2%) | 5 | (1.0%) |
|  | HYPOALBUMINEMIA | Total | 2 | (0.8%) | 1 | (0.4%) | 3 | (0.6%) |
|  |  | G1 | 1 | (0.4%) | 1 | (0.4%) | 2 | (0.4%) |
|  |  | G2 | 1 | (0.4%) | 0 | (0.0%) | 1 | (0.2%) |
|  | HYPOCALCEMIA | Total | 3 | (1.2%) | 10 | (4.0%) | 13 | (2.6%) |
|  |  | G1 | 3 | (1.2%) | 9 | (3.6%) | 12 | (2.4%) |
|  |  | G3/4/5 | 0 | (0.0%) | 1 | (0.4%) | 1 | (0.2%) |
|  | HYPOGLYCEMIA | Total | 3 | (1.2%) | 2 | (0.8%) | 5 | (1.0%) |
|  |  | G1 | 3 | (1.2%) | 2 | (0.8%) | 5 | (1.0%) |
|  | HYPOKALEMIA | Total | 3 | (1.2%) | 2 | (0.8%) | 5 | (1.0%) |
|  |  | G1 | 3 | (1.2%) | 2 | (0.8%) | 5 | (1.0%) |
|  | HYPONATREMIA | Total | 2 | (0.8%) | 1 | (0.4%) | 3 | (0.6%) |
|  |  | G1 | 2 | (0.8%) | 1 | (0.4%) | 3 | (0.6%) |
|  | HYPOPHOSPHATEMIA | Total | 1 | (0.4%) | 9 | (3.6%) | 10 | (2.0%) |
|  |  | G1 | 0 | (0.0%) | 7 | (2.8%) | 7 | (1.4%) |
|  |  | G2 | 1 | (0.4%) | 2 | (0.8%) | 3 | (0.6%) |
|  | IRON DEFICIENCY | Total | 0 | (0.0%) | 1 | (0.4%) | 1 | (0.2%) |
|  |  | G2 | 0 | (0.0%) | 1 | (0.4%) | 1 | (0.2%) |
|  | OBESITY | Total | 1 | (0.4%) | 0 | (0.0%) | 1 | (0.2%) |
|  |  | G3/4/5 | 1 | (0.4%) | 0 | (0.0%) | 1 | (0.2%) |
|  | PHOSPHORE DECREASED | Total | 3 | (1.2%) | 1 | (0.4%) | 4 | (0.8%) |
|  |  | G1 | 3 | (1.2%) | 1 | (0.4%) | 4 | (0.8%) |
|  | TRIGLYCERIDES DECREASED | Total | 2 | (0.8%) | 0 | (0.0%) | 2 | (0.4%) |
|  |  | G1 | 2 | (0.8%) | 0 | (0.0%) | 2 | (0.4%) |
|  | VITAMIN DEFICIENCY | Total | 1 | (0.4%) | 1 | (0.4%) | 2 | (0.4%) |
|  |  | G1 | 1 | (0.4%) | 1 | (0.4%) | 2 | (0.4%) |
| MUSCULOSKELETAL AND CONNECTIVE TISSUE DISORDERS | Total | Total | 120 | (48.0%) | 74 | (29.5%) | 194 | (38.7%) |
|  |  | . | 2 | (0.8%) | 2 | (0.8%) | 4 | (0.8%) |
|  |  | G1 | 93 | (37.2%) | 51 | (20.3%) | 144 | (28.7%) |
|  |  | G2 | 35 | (14.0%) | 22 | (8.8%) | 57 | (11.4%) |
|  |  | G3/4/5 | 0 | (0.0%) | 2 | (0.8%) | 2 | (0.4%) |
|  | ANKLE SPRAIN | Total | 0 | (0.0%) | 1 | (0.4%) | 1 | (0.2%) |
|  |  | G1 | 0 | (0.0%) | 1 | (0.4%) | 1 | (0.2%) |
|  | ARTHRALGIA | Total | 64 | (25.6%) | 37 | (14.7%) | 101 | (20.2%) |
|  |  | . | 1 | (0.4%) | 0 | (0.0%) | 1 | (0.2%) |
|  |  | G1 | 48 | (19.2%) | 29 | (11.6%) | 77 | (15.4%) |
|  |  | G2 | 15 | (6.0%) | 8 | (3.2%) | 23 | (4.6%) |
|  | ARTHRITIS | Total | 5 | (2.0%) | 0 | (0.0%) | 5 | (1.0%) |
|  |  | G1 | 3 | (1.2%) | 0 | (0.0%) | 3 | (0.6%) |
|  |  | G2 | 2 | (0.8%) | 0 | (0.0%) | 2 | (0.4%) |
|  | BACK PAIN | Total | 20 | (8.0%) | 10 | (4.0%) | 30 | (6.0%) |
|  |  | G1 | 12 | (4.8%) | 4 | (1.6%) | 16 | (3.2%) |
|  |  | G2 | 8 | (3.2%) | 5 | (2.0%) | 13 | (2.6%) |
|  |  | G3/4/5 | 0 | (0.0%) | 1 | (0.4%) | 1 | (0.2%) |
|  | BONE PAIN | Total | 10 | (4.0%) | 5 | (2.0%) | 15 | (3.0%) |
|  |  | G1 | 7 | (2.8%) | 3 | (1.2%) | 10 | (2.0%) |
|  |  | G2 | 3 | (1.2%) | 2 | (0.8%) | 5 | (1.0%) |
|  | CHEST WALL PAIN | Total | 2 | (0.8%) | 7 | (2.8%) | 9 | (1.8%) |
|  |  | . | 0 | (0.0%) | 1 | (0.4%) | 1 | (0.2%) |
|  |  | G1 | 1 | (0.4%) | 2 | (0.8%) | 3 | (0.6%) |
|  |  | G2 | 1 | (0.4%) | 4 | (1.6%) | 5 | (1.0%) |
|  | FIBROSIS DEEP CONNECTIVE TISSUE | Total | 0 | (0.0%) | 1 | (0.4%) | 1 | (0.2%) |
|  |  | G2 | 0 | (0.0%) | 1 | (0.4%) | 1 | (0.2%) |
|  | FLANK PAIN | Total | 1 | (0.4%) | 0 | (0.0%) | 1 | (0.2%) |
|  |  | G1 | 1 | (0.4%) | 0 | (0.0%) | 1 | (0.2%) |
|  | JOINT EFFUSION | Total | 1 | (0.4%) | 0 | (0.0%) | 1 | (0.2%) |
|  |  | G1 | 1 | (0.4%) | 0 | (0.0%) | 1 | (0.2%) |
|  | JOINT RANGE OF MOTION DECREASED | Total | 2 | (0.8%) | 1 | (0.4%) | 3 | (0.6%) |
|  |  | G1 | 1 | (0.4%) | 1 | (0.4%) | 2 | (0.4%) |
|  |  | G2 | 1 | (0.4%) | 0 | (0.0%) | 1 | (0.2%) |
|  | LIGAMENT TEAR | Total | 1 | (0.4%) | 0 | (0.0%) | 1 | (0.2%) |
|  |  | G2 | 1 | (0.4%) | 0 | (0.0%) | 1 | (0.2%) |
|  | LIMB DISCOMFORT | Total | 1 | (0.4%) | 0 | (0.0%) | 1 | (0.2%) |
|  |  | G1 | 1 | (0.4%) | 0 | (0.0%) | 1 | (0.2%) |
|  | MUSCLE WEAKNESS LEFT-SIDED | Total | 1 | (0.4%) | 0 | (0.0%) | 1 | (0.2%) |
|  |  | G1 | 1 | (0.4%) | 0 | (0.0%) | 1 | (0.2%) |
|  | MUSCLE WEAKNESS RIGHT-SIDED | Total | 1 | (0.4%) | 0 | (0.0%) | 1 | (0.2%) |
|  |  | G1 | 1 | (0.4%) | 0 | (0.0%) | 1 | (0.2%) |
|  | MUSCLE WEAKNESS UPPER LIMB | Total | 1 | (0.4%) | 0 | (0.0%) | 1 | (0.2%) |
|  |  | G2 | 1 | (0.4%) | 0 | (0.0%) | 1 | (0.2%) |
|  | MUSCULOSKELETAL DEFORMITY | Total | 3 | (1.2%) | 0 | (0.0%) | 3 | (0.6%) |
|  |  | G1 | 2 | (0.8%) | 0 | (0.0%) | 2 | (0.4%) |
|  |  | G2 | 1 | (0.4%) | 0 | (0.0%) | 1 | (0.2%) |
|  | MYALGIA | Total | 32 | (12.8%) | 19 | (7.6%) | 51 | (10.2%) |
|  |  | . | 1 | (0.4%) | 0 | (0.0%) | 1 | (0.2%) |
|  |  | G1 | 25 | (10.0%) | 16 | (6.4%) | 41 | (8.2%) |
|  |  | G2 | 6 | (2.4%) | 3 | (1.2%) | 9 | (1.8%) |
|  | MYOSITIS | Total | 1 | (0.4%) | 0 | (0.0%) | 1 | (0.2%) |
|  |  | G2 | 1 | (0.4%) | 0 | (0.0%) | 1 | (0.2%) |
|  | NECK PAIN | Total | 1 | (0.4%) | 1 | (0.4%) | 2 | (0.4%) |
|  |  | G1 | 1 | (0.4%) | 1 | (0.4%) | 2 | (0.4%) |
|  | OSTEOPOROSIS | Total | 2 | (0.8%) | 0 | (0.0%) | 2 | (0.4%) |
|  |  | G1 | 2 | (0.8%) | 0 | (0.0%) | 2 | (0.4%) |
|  | PAIN IN EXTREMITY | Total | 7 | (2.8%) | 4 | (1.6%) | 11 | (2.2%) |
|  |  | . | 0 | (0.0%) | 1 | (0.4%) | 1 | (0.2%) |
|  |  | G1 | 7 | (2.8%) | 2 | (0.8%) | 9 | (1.8%) |
|  |  | G2 | 0 | (0.0%) | 1 | (0.4%) | 1 | (0.2%) |
|  | POSTERIOR TIBIAL TENDONITIS | Total | 1 | (0.4%) | 0 | (0.0%) | 1 | (0.2%) |
|  |  | G2 | 1 | (0.4%) | 0 | (0.0%) | 1 | (0.2%) |
|  | TENDINITIS | Total | 2 | (0.8%) | 1 | (0.4%) | 3 | (0.6%) |
|  |  | G1 | 2 | (0.8%) | 0 | (0.0%) | 2 | (0.4%) |
|  |  | G3/4/5 | 0 | (0.0%) | 1 | (0.4%) | 1 | (0.2%) |
| NEOPLASMS BENIGN, MALIGNANT AND UNSPECIFIED (INCL CYSTS AND POLYPS) | Total | Total | 7 | (2.8%) | 8 | (3.2%) | 15 | (3.0%) |
|  |  | . | 1 | (0.4%) | 0 | (0.0%) | 1 | (0.2%) |
|  |  | G1 | 2 | (0.8%) | 5 | (2.0%) | 7 | (1.4%) |
|  |  | G2 | 3 | (1.2%) | 3 | (1.2%) | 6 | (1.2%) |
|  |  | G3/4/5 | 2 | (0.8%) | 0 | (0.0%) | 2 | (0.4%) |
|  | BENIGN NEOPLASM OF BREAST | Total | 1 | (0.4%) | 0 | (0.0%) | 1 | (0.2%) |
|  |  | G2 | 1 | (0.4%) | 0 | (0.0%) | 1 | (0.2%) |
|  | BOWEN'S DISEASE | Total | 1 | (0.4%) | 0 | (0.0%) | 1 | (0.2%) |
|  |  | G2 | 1 | (0.4%) | 0 | (0.0%) | 1 | (0.2%) |
|  | BREAST CANCER | Total | 1 | (0.4%) | 0 | (0.0%) | 1 | (0.2%) |
|  |  | G3/4/5 | 1 | (0.4%) | 0 | (0.0%) | 1 | (0.2%) |
|  | BREAST CYST | Total | 1 | (0.4%) | 1 | (0.4%) | 2 | (0.4%) |
|  |  | G1 | 1 | (0.4%) | 1 | (0.4%) | 2 | (0.4%) |
|  | CYST | Total | 1 | (0.4%) | 3 | (1.2%) | 4 | (0.8%) |
|  |  | . | 1 | (0.4%) | 0 | (0.0%) | 1 | (0.2%) |
|  |  | G1 | 0 | (0.0%) | 2 | (0.8%) | 2 | (0.4%) |
|  |  | G2 | 0 | (0.0%) | 1 | (0.4%) | 1 | (0.2%) |
|  | ENDOMETRIAL HYPERPLASIA | Total | 0 | (0.0%) | 1 | (0.4%) | 1 | (0.2%) |
|  |  | G1 | 0 | (0.0%) | 1 | (0.4%) | 1 | (0.2%) |
|  | HAEMANGIOMA OF LIVER | Total | 1 | (0.4%) | 0 | (0.0%) | 1 | (0.2%) |
|  |  | G1 | 1 | (0.4%) | 0 | (0.0%) | 1 | (0.2%) |
|  | LIPOMA | Total | 1 | (0.4%) | 0 | (0.0%) | 1 | (0.2%) |
|  |  | G1 | 1 | (0.4%) | 0 | (0.0%) | 1 | (0.2%) |
|  | MYOMA | Total | 1 | (0.4%) | 0 | (0.0%) | 1 | (0.2%) |
|  |  | G2 | 1 | (0.4%) | 0 | (0.0%) | 1 | (0.2%) |
|  | NEOPLASM | Total | 0 | (0.0%) | 1 | (0.4%) | 1 | (0.2%) |
|  |  | G2 | 0 | (0.0%) | 1 | (0.4%) | 1 | (0.2%) |
|  | POLYP | Total | 0 | (0.0%) | 1 | (0.4%) | 1 | (0.2%) |
|  |  | G2 | 0 | (0.0%) | 1 | (0.4%) | 1 | (0.2%) |
|  | SKIN CYST | Total | 0 | (0.0%) | 1 | (0.4%) | 1 | (0.2%) |
|  |  | G1 | 0 | (0.0%) | 1 | (0.4%) | 1 | (0.2%) |
|  | TUMOUR PROGRESSION | Total | 1 | (0.4%) | 0 | (0.0%) | 1 | (0.2%) |
|  |  | G3/4/5 | 1 | (0.4%) | 0 | (0.0%) | 1 | (0.2%) |
|  | UTERINE POLYP | Total | 1 | (0.4%) | 0 | (0.0%) | 1 | (0.2%) |
|  |  | G2 | 1 | (0.4%) | 0 | (0.0%) | 1 | (0.2%) |
| NERVOUS SYSTEM DISORDERS | Total | Total | 74 | (29.6%) | 104 | (41.4%) | 178 | (35.5%) |
|  |  | . | 1 | (0.4%) | 3 | (1.2%) | 4 | (0.8%) |
|  |  | G1 | 63 | (25.2%) | 85 | (33.9%) | 148 | (29.5%) |
|  |  | G2 | 18 | (7.2%) | 21 | (8.4%) | 39 | (7.8%) |
|  |  | G3/4/5 | 0 | (0.0%) | 3 | (1.2%) | 3 | (0.6%) |
|  | AMNESIA | Total | 0 | (0.0%) | 2 | (0.8%) | 2 | (0.4%) |
|  |  | G1 | 0 | (0.0%) | 2 | (0.8%) | 2 | (0.4%) |
|  | APHONIA | Total | 2 | (0.8%) | 2 | (0.8%) | 4 | (0.8%) |
|  |  | G1 | 2 | (0.8%) | 1 | (0.4%) | 3 | (0.6%) |
|  |  | G3/4/5 | 0 | (0.0%) | 1 | (0.4%) | 1 | (0.2%) |
|  | BURNING MOUTH | Total | 1 | (0.4%) | 1 | (0.4%) | 2 | (0.4%) |
|  |  | G1 | 1 | (0.4%) | 1 | (0.4%) | 2 | (0.4%) |
|  | COGNITIVE DISTURBANCE | Total | 0 | (0.0%) | 3 | (1.2%) | 3 | (0.6%) |
|  |  | G1 | 0 | (0.0%) | 3 | (1.2%) | 3 | (0.6%) |
|  | CONCENTRATION IMPAIRMENT | Total | 2 | (0.8%) | 1 | (0.4%) | 3 | (0.6%) |
|  |  | G1 | 1 | (0.4%) | 1 | (0.4%) | 2 | (0.4%) |
|  |  | G2 | 1 | (0.4%) | 0 | (0.0%) | 1 | (0.2%) |
|  | DIZZINESS | Total | 10 | (4.0%) | 5 | (2.0%) | 15 | (3.0%) |
|  |  | G1 | 10 | (4.0%) | 5 | (2.0%) | 15 | (3.0%) |
|  | DYSESTHESIA | Total | 2 | (0.8%) | 1 | (0.4%) | 3 | (0.6%) |
|  |  | G1 | 1 | (0.4%) | 1 | (0.4%) | 2 | (0.4%) |
|  |  | G2 | 1 | (0.4%) | 0 | (0.0%) | 1 | (0.2%) |
|  | DYSGEUSIA | Total | 11 | (4.4%) | 28 | (11.2%) | 39 | (7.8%) |
|  |  | G1 | 11 | (4.4%) | 26 | (10.4%) | 37 | (7.4%) |
|  |  | G2 | 0 | (0.0%) | 1 | (0.4%) | 1 | (0.2%) |
|  |  | G3/4/5 | 0 | (0.0%) | 1 | (0.4%) | 1 | (0.2%) |
|  | DYSPHASIA | Total | 0 | (0.0%) | 1 | (0.4%) | 1 | (0.2%) |
|  |  | G1 | 0 | (0.0%) | 1 | (0.4%) | 1 | (0.2%) |
|  | EPILEPTIC ATTACK | Total | 0 | (0.0%) | 1 | (0.4%) | 1 | (0.2%) |
|  |  | . | 0 | (0.0%) | 1 | (0.4%) | 1 | (0.2%) |
|  | HEADACHE | Total | 45 | (18.0%) | 62 | (24.7%) | 107 | (21.4%) |
|  |  | . | 0 | (0.0%) | 2 | (0.8%) | 2 | (0.4%) |
|  |  | G1 | 33 | (13.2%) | 44 | (17.5%) | 77 | (15.4%) |
|  |  | G2 | 12 | (4.8%) | 15 | (6.0%) | 27 | (5.4%) |
|  |  | G3/4/5 | 0 | (0.0%) | 1 | (0.4%) | 1 | (0.2%) |
|  | HYPOESTHESIA | Total | 1 | (0.4%) | 0 | (0.0%) | 1 | (0.2%) |
|  |  | G1 | 1 | (0.4%) | 0 | (0.0%) | 1 | (0.2%) |
|  | ISCHEMIA CEREBROVASCULAR | Total | 1 | (0.4%) | 0 | (0.0%) | 1 | (0.2%) |
|  |  | . | 1 | (0.4%) | 0 | (0.0%) | 1 | (0.2%) |
|  | LETHARGY | Total | 0 | (0.0%) | 2 | (0.8%) | 2 | (0.4%) |
|  |  | G1 | 0 | (0.0%) | 1 | (0.4%) | 1 | (0.2%) |
|  |  | G2 | 0 | (0.0%) | 1 | (0.4%) | 1 | (0.2%) |
|  | MEMORY IMPAIRMENT | Total | 5 | (2.0%) | 4 | (1.6%) | 9 | (1.8%) |
|  |  | G1 | 5 | (2.0%) | 4 | (1.6%) | 9 | (1.8%) |
|  | NEURALGIA | Total | 0 | (0.0%) | 1 | (0.4%) | 1 | (0.2%) |
|  |  | G1 | 0 | (0.0%) | 1 | (0.4%) | 1 | (0.2%) |
|  | PARESTHESIA | Total | 9 | (3.6%) | 5 | (2.0%) | 14 | (2.8%) |
|  |  | G1 | 7 | (2.8%) | 4 | (1.6%) | 11 | (2.2%) |
|  |  | G2 | 2 | (0.8%) | 1 | (0.4%) | 3 | (0.6%) |
|  | PERIPHERAL MOTOR NEUROPATHY | Total | 1 | (0.4%) | 1 | (0.4%) | 2 | (0.4%) |
|  |  | G2 | 1 | (0.4%) | 1 | (0.4%) | 2 | (0.4%) |
|  | PERIPHERAL SENSORY NEUROPATHY | Total | 5 | (2.0%) | 6 | (2.4%) | 11 | (2.2%) |
|  |  | G1 | 4 | (1.6%) | 5 | (2.0%) | 9 | (1.8%) |
|  |  | G2 | 1 | (0.4%) | 1 | (0.4%) | 2 | (0.4%) |
|  | SINUS PAIN | Total | 0 | (0.0%) | 1 | (0.4%) | 1 | (0.2%) |
|  |  | G2 | 0 | (0.0%) | 1 | (0.4%) | 1 | (0.2%) |
|  | SOMNOLENCE | Total | 0 | (0.0%) | 1 | (0.4%) | 1 | (0.2%) |
|  |  | G2 | 0 | (0.0%) | 1 | (0.4%) | 1 | (0.2%) |
|  | TREMOR | Total | 2 | (0.8%) | 1 | (0.4%) | 3 | (0.6%) |
|  |  | G1 | 2 | (0.8%) | 1 | (0.4%) | 3 | (0.6%) |
|  | TRISMUS | Total | 0 | (0.0%) | 1 | (0.4%) | 1 | (0.2%) |
|  |  | G1 | 0 | (0.0%) | 1 | (0.4%) | 1 | (0.2%) |
|  | VASOVAGAL REACTION | Total | 1 | (0.4%) | 0 | (0.0%) | 1 | (0.2%) |
|  |  | G2 | 1 | (0.4%) | 0 | (0.0%) | 1 | (0.2%) |
| PSYCHIATRIC DISORDERS | Total | Total | 55 | (22.0%) | 31 | (12.4%) | 86 | (17.2%) |
|  |  | G1 | 44 | (17.6%) | 23 | (9.2%) | 67 | (13.4%) |
|  |  | G2 | 14 | (5.6%) | 8 | (3.2%) | 22 | (4.4%) |
|  |  | G3/4/5 | 2 | (0.8%) | 0 | (0.0%) | 2 | (0.4%) |
|  | AGITATION | Total | 1 | (0.4%) | 0 | (0.0%) | 1 | (0.2%) |
|  |  | G1 | 1 | (0.4%) | 0 | (0.0%) | 1 | (0.2%) |
|  | ANXIETY | Total | 14 | (5.6%) | 8 | (3.2%) | 22 | (4.4%) |
|  |  | G1 | 10 | (4.0%) | 4 | (1.6%) | 14 | (2.8%) |
|  |  | G2 | 4 | (1.6%) | 4 | (1.6%) | 8 | (1.6%) |
|  | DEPRESSION | Total | 6 | (2.4%) | 4 | (1.6%) | 10 | (2.0%) |
|  |  | G1 | 3 | (1.2%) | 3 | (1.2%) | 6 | (1.2%) |
|  |  | G2 | 3 | (1.2%) | 1 | (0.4%) | 4 | (0.8%) |
|  | INSOMNIA | Total | 37 | (14.8%) | 20 | (8.0%) | 57 | (11.4%) |
|  |  | G1 | 28 | (11.2%) | 16 | (6.4%) | 44 | (8.8%) |
|  |  | G2 | 9 | (3.6%) | 4 | (1.6%) | 13 | (2.6%) |
|  | LIBIDO DECREASED | Total | 4 | (1.6%) | 1 | (0.4%) | 5 | (1.0%) |
|  |  | G1 | 3 | (1.2%) | 1 | (0.4%) | 4 | (0.8%) |
|  |  | G2 | 1 | (0.4%) | 0 | (0.0%) | 1 | (0.2%) |
|  | MOOD DISORDER | Total | 2 | (0.8%) | 2 | (0.8%) | 4 | (0.8%) |
|  |  | G1 | 2 | (0.8%) | 1 | (0.4%) | 3 | (0.6%) |
|  |  | G2 | 0 | (0.0%) | 1 | (0.4%) | 1 | (0.2%) |
|  | PSYCHIATRIC DISORDERS - OTHER, SPECIFY | Total | 1 | (0.4%) | 0 | (0.0%) | 1 | (0.2%) |
|  |  | G3/4/5 | 1 | (0.4%) | 0 | (0.0%) | 1 | (0.2%) |
|  | SUICIDE ATTEMPT | Total | 1 | (0.4%) | 0 | (0.0%) | 1 | (0.2%) |
|  |  | G3/4/5 | 1 | (0.4%) | 0 | (0.0%) | 1 | (0.2%) |
| RENAL AND URINARY DISORDERS | Total | Total | 13 | (5.2%) | 20 | (8.0%) | 33 | (6.6%) |
|  |  | G1 | 12 | (4.8%) | 14 | (5.6%) | 26 | (5.2%) |
|  |  | G2 | 1 | (0.4%) | 6 | (2.4%) | 7 | (1.4%) |
|  | CYSTITIS NONINFECTIVE | Total | 3 | (1.2%) | 5 | (2.0%) | 8 | (1.6%) |
|  |  | G1 | 2 | (0.8%) | 2 | (0.8%) | 4 | (0.8%) |
|  |  | G2 | 1 | (0.4%) | 3 | (1.2%) | 4 | (0.8%) |
|  | UREA DECREASED | Total | 1 | (0.4%) | 2 | (0.8%) | 3 | (0.6%) |
|  |  | G1 | 1 | (0.4%) | 2 | (0.8%) | 3 | (0.6%) |
|  | UREA INCREASED | Total | 5 | (2.0%) | 2 | (0.8%) | 7 | (1.4%) |
|  |  | G1 | 5 | (2.0%) | 2 | (0.8%) | 7 | (1.4%) |
|  | URINARY FREQUENCY | Total | 2 | (0.8%) | 5 | (2.0%) | 7 | (1.4%) |
|  |  | G1 | 2 | (0.8%) | 4 | (1.6%) | 6 | (1.2%) |
|  |  | G2 | 0 | (0.0%) | 1 | (0.4%) | 1 | (0.2%) |
|  | URINARY INCONTINENCE | Total | 2 | (0.8%) | 1 | (0.4%) | 3 | (0.6%) |
|  |  | G1 | 2 | (0.8%) | 1 | (0.4%) | 3 | (0.6%) |
|  | URINARY RETENTION | Total | 0 | (0.0%) | 1 | (0.4%) | 1 | (0.2%) |
|  |  | G2 | 0 | (0.0%) | 1 | (0.4%) | 1 | (0.2%) |
|  | URINARY TRACT PAIN | Total | 0 | (0.0%) | 3 | (1.2%) | 3 | (0.6%) |
|  |  | G1 | 0 | (0.0%) | 2 | (0.8%) | 2 | (0.4%) |
|  |  | G2 | 0 | (0.0%) | 1 | (0.4%) | 1 | (0.2%) |
|  | URINE DISCOLORATION | Total | 0 | (0.0%) | 1 | (0.4%) | 1 | (0.2%) |
|  |  | G1 | 0 | (0.0%) | 1 | (0.4%) | 1 | (0.2%) |
| REPRODUCTIVE SYSTEM AND BREAST DISORDERS | Total | Total | 41 | (16.4%) | 23 | (9.2%) | 64 | (12.8%) |
|  |  | G1 | 31 | (12.4%) | 13 | (5.2%) | 44 | (8.8%) |
|  |  | G2 | 11 | (4.4%) | 12 | (4.8%) | 23 | (4.6%) |
|  | BREAST PAIN | Total | 4 | (1.6%) | 6 | (2.4%) | 10 | (2.0%) |
|  |  | G1 | 4 | (1.6%) | 5 | (2.0%) | 9 | (1.8%) |
|  |  | G2 | 0 | (0.0%) | 1 | (0.4%) | 1 | (0.2%) |
|  | DYSPAREUNIA | Total | 1 | (0.4%) | 2 | (0.8%) | 3 | (0.6%) |
|  |  | G1 | 1 | (0.4%) | 1 | (0.4%) | 2 | (0.4%) |
|  |  | G2 | 0 | (0.0%) | 1 | (0.4%) | 1 | (0.2%) |
|  | IRREGULAR MENSTRUATION | Total | 4 | (1.6%) | 1 | (0.4%) | 5 | (1.0%) |
|  |  | G1 | 3 | (1.2%) | 1 | (0.4%) | 4 | (0.8%) |
|  |  | G2 | 1 | (0.4%) | 0 | (0.0%) | 1 | (0.2%) |
|  | MENORRHAGIA | Total | 8 | (3.2%) | 1 | (0.4%) | 9 | (1.8%) |
|  |  | G1 | 6 | (2.4%) | 1 | (0.4%) | 7 | (1.4%) |
|  |  | G2 | 2 | (0.8%) | 0 | (0.0%) | 2 | (0.4%) |
|  | PELVIC PAIN | Total | 3 | (1.2%) | 4 | (1.6%) | 7 | (1.4%) |
|  |  | G1 | 3 | (1.2%) | 2 | (0.8%) | 5 | (1.0%) |
|  |  | G2 | 0 | (0.0%) | 2 | (0.8%) | 2 | (0.4%) |
|  | THICKENING ENDOMETRIUM | Total | 0 | (0.0%) | 1 | (0.4%) | 1 | (0.2%) |
|  |  | G1 | 0 | (0.0%) | 1 | (0.4%) | 1 | (0.2%) |
|  | VAGINAL DISCHARGE | Total | 5 | (2.0%) | 1 | (0.4%) | 6 | (1.2%) |
|  |  | G1 | 5 | (2.0%) | 0 | (0.0%) | 5 | (1.0%) |
|  |  | G2 | 0 | (0.0%) | 1 | (0.4%) | 1 | (0.2%) |
|  | VAGINAL DRYNESS | Total | 15 | (6.0%) | 7 | (2.8%) | 22 | (4.4%) |
|  |  | G1 | 10 | (4.0%) | 2 | (0.8%) | 12 | (2.4%) |
|  |  | G2 | 5 | (2.0%) | 5 | (2.0%) | 10 | (2.0%) |
|  | VAGINAL FISTULA | Total | 0 | (0.0%) | 1 | (0.4%) | 1 | (0.2%) |
|  |  | G2 | 0 | (0.0%) | 1 | (0.4%) | 1 | (0.2%) |
|  | VAGINAL HEMORRHAGE | Total | 3 | (1.2%) | 2 | (0.8%) | 5 | (1.0%) |
|  |  | G1 | 2 | (0.8%) | 2 | (0.8%) | 4 | (0.8%) |
|  |  | G2 | 1 | (0.4%) | 0 | (0.0%) | 1 | (0.2%) |
|  | VAGINAL INFLAMMATION | Total | 3 | (1.2%) | 1 | (0.4%) | 4 | (0.8%) |
|  |  | G1 | 1 | (0.4%) | 0 | (0.0%) | 1 | (0.2%) |
|  |  | G2 | 2 | (0.8%) | 1 | (0.4%) | 3 | (0.6%) |
|  | VAGINAL PAIN | Total | 1 | (0.4%) | 1 | (0.4%) | 2 | (0.4%) |
|  |  | G1 | 1 | (0.4%) | 0 | (0.0%) | 1 | (0.2%) |
|  |  | G2 | 0 | (0.0%) | 1 | (0.4%) | 1 | (0.2%) |
| RESPIRATORY, THORACIC AND MEDIASTINAL DISORDERS | Total | Total | 85 | (34.0%) | 122 | (48.6%) | 207 | (41.3%) |
|  |  | . | 1 | (0.4%) | 0 | (0.0%) | 1 | (0.2%) |
|  |  | G1 | 77 | (30.8%) | 98 | (39.0%) | 175 | (34.9%) |
|  |  | G2 | 15 | (6.0%) | 37 | (14.7%) | 52 | (10.4%) |
|  |  | G3/4/5 | 2 | (0.8%) | 5 | (2.0%) | 7 | (1.4%) |
|  | ALLERGIC RHINITIS | Total | 3 | (1.2%) | 0 | (0.0%) | 3 | (0.6%) |
|  |  | G1 | 3 | (1.2%) | 0 | (0.0%) | 3 | (0.6%) |
|  | BRONCHIAL OBSTRUCTION | Total | 0 | (0.0%) | 1 | (0.4%) | 1 | (0.2%) |
|  |  | G1 | 0 | (0.0%) | 1 | (0.4%) | 1 | (0.2%) |
|  | COUGH | Total | 55 | (22.0%) | 88 | (35.1%) | 143 | (28.5%) |
|  |  | . | 1 | (0.4%) | 0 | (0.0%) | 1 | (0.2%) |
|  |  | G1 | 44 | (17.6%) | 61 | (24.3%) | 105 | (21.0%) |
|  |  | G2 | 9 | (3.6%) | 27 | (10.8%) | 36 | (7.2%) |
|  |  | G3/4/5 | 1 | (0.4%) | 0 | (0.0%) | 1 | (0.2%) |
|  | DYSPNEA | Total | 27 | (10.8%) | 47 | (18.7%) | 74 | (14.8%) |
|  |  | G1 | 24 | (9.6%) | 37 | (14.7%) | 61 | (12.2%) |
|  |  | G2 | 3 | (1.2%) | 9 | (3.6%) | 12 | (2.4%) |
|  |  | G3/4/5 | 0 | (0.0%) | 1 | (0.4%) | 1 | (0.2%) |
|  | EPISTAXIS | Total | 5 | (2.0%) | 13 | (5.2%) | 18 | (3.6%) |
|  |  | G1 | 4 | (1.6%) | 13 | (5.2%) | 17 | (3.4%) |
|  |  | G2 | 1 | (0.4%) | 0 | (0.0%) | 1 | (0.2%) |
|  | HOARSENESS | Total | 1 | (0.4%) | 0 | (0.0%) | 1 | (0.2%) |
|  |  | G1 | 1 | (0.4%) | 0 | (0.0%) | 1 | (0.2%) |
|  | LARYNGEAL INFLAMMATION | Total | 1 | (0.4%) | 1 | (0.4%) | 2 | (0.4%) |
|  |  | G1 | 1 | (0.4%) | 1 | (0.4%) | 2 | (0.4%) |
|  | NASAL CONGESTION | Total | 0 | (0.0%) | 1 | (0.4%) | 1 | (0.2%) |
|  |  | G2 | 0 | (0.0%) | 1 | (0.4%) | 1 | (0.2%) |
|  | PHARYNGEAL MUCOSITIS | Total | 0 | (0.0%) | 1 | (0.4%) | 1 | (0.2%) |
|  |  | G3/4/5 | 0 | (0.0%) | 1 | (0.4%) | 1 | (0.2%) |
|  | PHARYNGOLARYNGEAL PAIN | Total | 1 | (0.4%) | 1 | (0.4%) | 2 | (0.4%) |
|  |  | G1 | 1 | (0.4%) | 1 | (0.4%) | 2 | (0.4%) |
|  | PLEURAL EFFUSION | Total | 0 | (0.0%) | 1 | (0.4%) | 1 | (0.2%) |
|  |  | G2 | 0 | (0.0%) | 1 | (0.4%) | 1 | (0.2%) |
|  | PNEUMONITIS | Total | 2 | (0.8%) | 8 | (3.2%) | 10 | (2.0%) |
|  |  | G1 | 0 | (0.0%) | 1 | (0.4%) | 1 | (0.2%) |
|  |  | G2 | 2 | (0.8%) | 3 | (1.2%) | 5 | (1.0%) |
|  |  | G3/4/5 | 0 | (0.0%) | 4 | (1.6%) | 4 | (0.8%) |
|  | PNEUMOTHORAX | Total | 1 | (0.4%) | 0 | (0.0%) | 1 | (0.2%) |
|  |  | G3/4/5 | 1 | (0.4%) | 0 | (0.0%) | 1 | (0.2%) |
|  | PRODUCTIVE COUGH | Total | 0 | (0.0%) | 1 | (0.4%) | 1 | (0.2%) |
|  |  | G1 | 0 | (0.0%) | 1 | (0.4%) | 1 | (0.2%) |
|  | RESPIRATORY FAILURE | Total | 12 | (4.8%) | 4 | (1.6%) | 16 | (3.2%) |
|  |  | G1 | 12 | (4.8%) | 3 | (1.2%) | 15 | (3.0%) |
|  |  | G2 | 0 | (0.0%) | 1 | (0.4%) | 1 | (0.2%) |
|  | SINUS DISORDER | Total | 1 | (0.4%) | 1 | (0.4%) | 2 | (0.4%) |
|  |  | G1 | 1 | (0.4%) | 1 | (0.4%) | 2 | (0.4%) |
|  | SNEEZING | Total | 1 | (0.4%) | 0 | (0.0%) | 1 | (0.2%) |
|  |  | G1 | 1 | (0.4%) | 0 | (0.0%) | 1 | (0.2%) |
|  | SORE THROAT | Total | 10 | (4.0%) | 14 | (5.6%) | 24 | (4.8%) |
|  |  | G1 | 10 | (4.0%) | 13 | (5.2%) | 23 | (4.6%) |
|  |  | G2 | 0 | (0.0%) | 1 | (0.4%) | 1 | (0.2%) |
|  | VOICE ALTERATION | Total | 1 | (0.4%) | 0 | (0.0%) | 1 | (0.2%) |
|  |  | G1 | 1 | (0.4%) | 0 | (0.0%) | 1 | (0.2%) |
|  | WHEEZING | Total | 1 | (0.4%) | 1 | (0.4%) | 2 | (0.4%) |
|  |  | G1 | 0 | (0.0%) | 1 | (0.4%) | 1 | (0.2%) |
|  |  | G2 | 1 | (0.4%) | 0 | (0.0%) | 1 | (0.2%) |
| SKIN AND SUBCUTANEOUS TISSUE DISORDERS | Total | Total | 88 | (35.2%) | 150 | (59.8%) | 238 | (47.5%) |
|  |  | G1 | 80 | (32.0%) | 129 | (51.4%) | 209 | (41.7%) |
|  |  | G2 | 14 | (5.6%) | 41 | (16.3%) | 55 | (11.0%) |
|  |  | G3/4/5 | 2 | (0.8%) | 4 | (1.6%) | 6 | (1.2%) |
|  | ALOPECIA | Total | 8 | (3.2%) | 8 | (3.2%) | 16 | (3.2%) |
|  |  | G1 | 5 | (2.0%) | 7 | (2.8%) | 12 | (2.4%) |
|  |  | G2 | 3 | (1.2%) | 1 | (0.4%) | 4 | (0.8%) |
|  | BRITTLE NAILS | Total | 3 | (1.2%) | 1 | (0.4%) | 4 | (0.8%) |
|  |  | G1 | 3 | (1.2%) | 1 | (0.4%) | 4 | (0.8%) |
|  | CUTANEOUS LESION | Total | 0 | (0.0%) | 1 | (0.4%) | 1 | (0.2%) |
|  |  | G1 | 0 | (0.0%) | 1 | (0.4%) | 1 | (0.2%) |
|  | DERMATOMYCOSIS, UNSPECIED | Total | 1 | (0.4%) | 0 | (0.0%) | 1 | (0.2%) |
|  |  | G1 | 1 | (0.4%) | 0 | (0.0%) | 1 | (0.2%) |
|  | DRY SKIN | Total | 22 | (8.8%) | 52 | (20.7%) | 74 | (14.8%) |
|  |  | G1 | 21 | (8.4%) | 46 | (18.3%) | 67 | (13.4%) |
|  |  | G2 | 1 | (0.4%) | 6 | (2.4%) | 7 | (1.4%) |
|  | ECZEMA | Total | 1 | (0.4%) | 3 | (1.2%) | 4 | (0.8%) |
|  |  | G1 | 1 | (0.4%) | 1 | (0.4%) | 2 | (0.4%) |
|  |  | G2 | 0 | (0.0%) | 2 | (0.8%) | 2 | (0.4%) |
|  | ERYTHEMA MULTIFORME | Total | 4 | (1.6%) | 15 | (6.0%) | 19 | (3.8%) |
|  |  | G1 | 4 | (1.6%) | 8 | (3.2%) | 12 | (2.4%) |
|  |  | G2 | 0 | (0.0%) | 7 | (2.8%) | 7 | (1.4%) |
|  | ERYTHRODERMA | Total | 4 | (1.6%) | 8 | (3.2%) | 12 | (2.4%) |
|  |  | G1 | 2 | (0.8%) | 5 | (2.0%) | 7 | (1.4%) |
|  |  | G2 | 1 | (0.4%) | 1 | (0.4%) | 2 | (0.4%) |
|  |  | G3/4/5 | 1 | (0.4%) | 2 | (0.8%) | 3 | (0.6%) |
|  | FOLLICULITIS | Total | 1 | (0.4%) | 1 | (0.4%) | 2 | (0.4%) |
|  |  | G1 | 1 | (0.4%) | 1 | (0.4%) | 2 | (0.4%) |
|  | HAIR THINNING | Total | 0 | (0.0%) | 1 | (0.4%) | 1 | (0.2%) |
|  |  | G1 | 0 | (0.0%) | 1 | (0.4%) | 1 | (0.2%) |
|  | HAND SWELLING | Total | 1 | (0.4%) | 0 | (0.0%) | 1 | (0.2%) |
|  |  | G1 | 1 | (0.4%) | 0 | (0.0%) | 1 | (0.2%) |
|  | HYPERKERATOSIS | Total | 0 | (0.0%) | 1 | (0.4%) | 1 | (0.2%) |
|  |  | G1 | 0 | (0.0%) | 1 | (0.4%) | 1 | (0.2%) |
|  | IRRITATION | Total | 1 | (0.4%) | 0 | (0.0%) | 1 | (0.2%) |
|  |  | G2 | 1 | (0.4%) | 0 | (0.0%) | 1 | (0.2%) |
|  | ITCH | Total | 4 | (1.6%) | 3 | (1.2%) | 7 | (1.4%) |
|  |  | G1 | 4 | (1.6%) | 3 | (1.2%) | 7 | (1.4%) |
|  | LEFT BREAST SKIN NECROSIS FOLLOWING RECONSTRUCTION SURGERY | Total | 1 | (0.4%) | 0 | (0.0%) | 1 | (0.2%) |
|  |  | G1 | 1 | (0.4%) | 0 | (0.0%) | 1 | (0.2%) |
|  | NAIL DISCOLORATION | Total | 0 | (0.0%) | 4 | (1.6%) | 4 | (0.8%) |
|  |  | G1 | 0 | (0.0%) | 2 | (0.8%) | 2 | (0.4%) |
|  |  | G2 | 0 | (0.0%) | 2 | (0.8%) | 2 | (0.4%) |
|  | NAIL LOSS | Total | 2 | (0.8%) | 9 | (3.6%) | 11 | (2.2%) |
|  |  | G1 | 2 | (0.8%) | 8 | (3.2%) | 10 | (2.0%) |
|  |  | G2 | 0 | (0.0%) | 1 | (0.4%) | 1 | (0.2%) |
|  | NAIL RIDGING | Total | 1 | (0.4%) | 13 | (5.2%) | 14 | (2.8%) |
|  |  | G1 | 1 | (0.4%) | 13 | (5.2%) | 14 | (2.8%) |
|  | PAIN OF SKIN | Total | 1 | (0.4%) | 0 | (0.0%) | 1 | (0.2%) |
|  |  | G1 | 1 | (0.4%) | 0 | (0.0%) | 1 | (0.2%) |
|  | PALMAR-PLANTAR ERYTHRODYSESTHESIA SYNDROME | Total | 2 | (0.8%) | 3 | (1.2%) | 5 | (1.0%) |
|  |  | G1 | 2 | (0.8%) | 2 | (0.8%) | 4 | (0.8%) |
|  |  | G2 | 0 | (0.0%) | 1 | (0.4%) | 1 | (0.2%) |
|  | PHOTOSENSITIVITY | Total | 2 | (0.8%) | 2 | (0.8%) | 4 | (0.8%) |
|  |  | G1 | 2 | (0.8%) | 2 | (0.8%) | 4 | (0.8%) |
|  | PRURITUS | Total | 18 | (7.2%) | 21 | (8.4%) | 39 | (7.8%) |
|  |  | G1 | 16 | (6.4%) | 17 | (6.8%) | 33 | (6.6%) |
|  |  | G2 | 2 | (0.8%) | 4 | (1.6%) | 6 | (1.2%) |
|  | PSORIASIS | Total | 0 | (0.0%) | 1 | (0.4%) | 1 | (0.2%) |
|  |  | G1 | 0 | (0.0%) | 1 | (0.4%) | 1 | (0.2%) |
|  | RASH | Total | 31 | (12.4%) | 80 | (31.9%) | 111 | (22.2%) |
|  |  | G1 | 28 | (11.2%) | 62 | (24.7%) | 90 | (18.0%) |
|  |  | G2 | 3 | (1.2%) | 16 | (6.4%) | 19 | (3.8%) |
|  |  | G3/4/5 | 0 | (0.0%) | 2 | (0.8%) | 2 | (0.4%) |
|  | ROSACEA | Total | 0 | (0.0%) | 1 | (0.4%) | 1 | (0.2%) |
|  |  | G2 | 0 | (0.0%) | 1 | (0.4%) | 1 | (0.2%) |
|  | SEBORRHEA | Total | 0 | (0.0%) | 1 | (0.4%) | 1 | (0.2%) |
|  |  | G1 | 0 | (0.0%) | 1 | (0.4%) | 1 | (0.2%) |
|  | SKIN DISORDERS | Total | 0 | (0.0%) | 2 | (0.8%) | 2 | (0.4%) |
|  |  | G1 | 0 | (0.0%) | 2 | (0.8%) | 2 | (0.4%) |
|  | SKIN HYPERPIGMENTATION | Total | 0 | (0.0%) | 1 | (0.4%) | 1 | (0.2%) |
|  |  | G1 | 0 | (0.0%) | 1 | (0.4%) | 1 | (0.2%) |
|  | SKIN INDURATION | Total | 1 | (0.4%) | 1 | (0.4%) | 2 | (0.4%) |
|  |  | G1 | 1 | (0.4%) | 0 | (0.0%) | 1 | (0.2%) |
|  |  | G2 | 0 | (0.0%) | 1 | (0.4%) | 1 | (0.2%) |
|  | SKIN ULCERATION | Total | 1 | (0.4%) | 2 | (0.8%) | 3 | (0.6%) |
|  |  | G1 | 0 | (0.0%) | 1 | (0.4%) | 1 | (0.2%) |
|  |  | G2 | 1 | (0.4%) | 1 | (0.4%) | 2 | (0.4%) |
|  | SPOT | Total | 0 | (0.0%) | 1 | (0.4%) | 1 | (0.2%) |
|  |  | G2 | 0 | (0.0%) | 1 | (0.4%) | 1 | (0.2%) |
|  | TINGLING | Total | 2 | (0.8%) | 2 | (0.8%) | 4 | (0.8%) |
|  |  | G1 | 1 | (0.4%) | 2 | (0.8%) | 3 | (0.6%) |
|  |  | G2 | 1 | (0.4%) | 0 | (0.0%) | 1 | (0.2%) |
|  | TOXIC EPIDERMAL NECROLYSIS | Total | 1 | (0.4%) | 0 | (0.0%) | 1 | (0.2%) |
|  |  | G1 | 1 | (0.4%) | 0 | (0.0%) | 1 | (0.2%) |
|  | TOXICODERMIA | Total | 2 | (0.8%) | 2 | (0.8%) | 4 | (0.8%) |
|  |  | G1 | 2 | (0.8%) | 2 | (0.8%) | 4 | (0.8%) |
|  | URTICARIA | Total | 2 | (0.8%) | 1 | (0.4%) | 3 | (0.6%) |
|  |  | G2 | 1 | (0.4%) | 1 | (0.4%) | 2 | (0.4%) |
|  |  | G3/4/5 | 1 | (0.4%) | 0 | (0.0%) | 1 | (0.2%) |
| SOCIAL CIRCUMSTANCES | Total | Total | 1 | (0.4%) | 0 | (0.0%) | 1 | (0.2%) |
|  |  | G1 | 1 | (0.4%) | 0 | (0.0%) | 1 | (0.2%) |
|  | MENOPAUSE | Total | 1 | (0.4%) | 0 | (0.0%) | 1 | (0.2%) |
|  |  | G1 | 1 | (0.4%) | 0 | (0.0%) | 1 | (0.2%) |
| SURGICAL AND MEDICAL PROCEDURES | Total | Total | 11 | (4.4%) | 6 | (2.4%) | 17 | (3.4%) |
|  |  | . | 3 | (1.2%) | 0 | (0.0%) | 3 | (0.6%) |
|  |  | G1 | 2 | (0.8%) | 2 | (0.8%) | 4 | (0.8%) |
|  |  | G2 | 5 | (2.0%) | 2 | (0.8%) | 7 | (1.4%) |
|  |  | G3/4/5 | 1 | (0.4%) | 2 | (0.8%) | 3 | (0.6%) |
|  | AERO-NIPPLE GRAFT | Total | 1 | (0.4%) | 0 | (0.0%) | 1 | (0.2%) |
|  |  | G1 | 1 | (0.4%) | 0 | (0.0%) | 1 | (0.2%) |
|  | BIOPSY | Total | 1 | (0.4%) | 0 | (0.0%) | 1 | (0.2%) |
|  |  | G2 | 1 | (0.4%) | 0 | (0.0%) | 1 | (0.2%) |
|  | BREAST | Total | 0 | (0.0%) | 1 | (0.4%) | 1 | (0.2%) |
|  |  | G2 | 0 | (0.0%) | 1 | (0.4%) | 1 | (0.2%) |
|  | BREAST PROSTHESIS RUPTURE | Total | 1 | (0.4%) | 0 | (0.0%) | 1 | (0.2%) |
|  |  | . | 1 | (0.4%) | 0 | (0.0%) | 1 | (0.2%) |
|  | BREAST RECONSTRUCTION | Total | 6 | (2.4%) | 3 | (1.2%) | 9 | (1.8%) |
|  |  | . | 1 | (0.4%) | 0 | (0.0%) | 1 | (0.2%) |
|  |  | G1 | 1 | (0.4%) | 2 | (0.8%) | 3 | (0.6%) |
|  |  | G2 | 3 | (1.2%) | 0 | (0.0%) | 3 | (0.6%) |
|  |  | G3/4/5 | 1 | (0.4%) | 1 | (0.4%) | 2 | (0.4%) |
|  | DENTAL FILLINGS | Total | 1 | (0.4%) | 0 | (0.0%) | 1 | (0.2%) |
|  |  | . | 1 | (0.4%) | 0 | (0.0%) | 1 | (0.2%) |
|  | DENTAL OPERATION | Total | 1 | (0.4%) | 0 | (0.0%) | 1 | (0.2%) |
|  |  | G2 | 1 | (0.4%) | 0 | (0.0%) | 1 | (0.2%) |
|  | HYSTERECTOMY | Total | 1 | (0.4%) | 0 | (0.0%) | 1 | (0.2%) |
|  |  | G2 | 1 | (0.4%) | 0 | (0.0%) | 1 | (0.2%) |
|  | RELAPSE - CEREBRAL LESION - HOSPITALIZATION | Total | 0 | (0.0%) | 1 | (0.4%) | 1 | (0.2%) |
|  |  | G3/4/5 | 0 | (0.0%) | 1 | (0.4%) | 1 | (0.2%) |
|  | STENT | Total | 0 | (0.0%) | 1 | (0.4%) | 1 | (0.2%) |
|  |  | G2 | 0 | (0.0%) | 1 | (0.4%) | 1 | (0.2%) |
| VASCULAR DISORDERS | Total | Total | 121 | (48.4%) | 94 | (37.5%) | 215 | (42.9%) |
|  |  | . | 1 | (0.4%) | 0 | (0.0%) | 1 | (0.2%) |
|  |  | G1 | 90 | (36.0%) | 61 | (24.3%) | 151 | (30.1%) |
|  |  | G2 | 35 | (14.0%) | 35 | (13.9%) | 70 | (14.0%) |
|  |  | G3/4/5 | 6 | (2.4%) | 9 | (3.6%) | 15 | (3.0%) |
|  | FLUSHING | Total | 4 | (1.6%) | 3 | (1.2%) | 7 | (1.4%) |
|  |  | G1 | 4 | (1.6%) | 2 | (0.8%) | 6 | (1.2%) |
|  |  | G2 | 0 | (0.0%) | 1 | (0.4%) | 1 | (0.2%) |
|  | HEAVY FEELING IN ARMS & LEGS | Total | 0 | (0.0%) | 1 | (0.4%) | 1 | (0.2%) |
|  |  | G1 | 0 | (0.0%) | 1 | (0.4%) | 1 | (0.2%) |
|  | HEMATOMA | Total | 2 | (0.8%) | 2 | (0.8%) | 4 | (0.8%) |
|  |  | G1 | 2 | (0.8%) | 2 | (0.8%) | 4 | (0.8%) |
|  | HOT FLASHES | Total | 91 | (36.4%) | 56 | (22.3%) | 147 | (29.3%) |
|  |  | G1 | 69 | (27.6%) | 39 | (15.5%) | 108 | (21.6%) |
|  |  | G2 | 20 | (8.0%) | 16 | (6.4%) | 36 | (7.2%) |
|  |  | G3/4/5 | 2 | (0.8%) | 1 | (0.4%) | 3 | (0.6%) |
|  | HYPERTENSION | Total | 21 | (8.4%) | 13 | (5.2%) | 34 | (6.8%) |
|  |  | G1 | 4 | (1.6%) | 2 | (0.8%) | 6 | (1.2%) |
|  |  | G2 | 13 | (5.2%) | 6 | (2.4%) | 19 | (3.8%) |
|  |  | G3/4/5 | 4 | (1.6%) | 5 | (2.0%) | 9 | (1.8%) |
|  | HYPOTENSION | Total | 1 | (0.4%) | 1 | (0.4%) | 2 | (0.4%) |
|  |  | . | 1 | (0.4%) | 0 | (0.0%) | 1 | (0.2%) |
|  |  | G1 | 0 | (0.0%) | 1 | (0.4%) | 1 | (0.2%) |
|  | LYMPHEDEMA | Total | 19 | (7.6%) | 30 | (12.0%) | 49 | (9.8%) |
|  |  | G1 | 15 | (6.0%) | 19 | (7.6%) | 34 | (6.8%) |
|  |  | G2 | 3 | (1.2%) | 11 | (4.4%) | 14 | (2.8%) |
|  |  | G3/4/5 | 1 | (0.4%) | 0 | (0.0%) | 1 | (0.2%) |
|  | SWELLING | Total | 1 | (0.4%) | 3 | (1.2%) | 4 | (0.8%) |
|  |  | G1 | 0 | (0.0%) | 2 | (0.8%) | 2 | (0.4%) |
|  |  | G2 | 1 | (0.4%) | 1 | (0.4%) | 2 | (0.4%) |
|  | VARICOSE | Total | 0 | (0.0%) | 1 | (0.4%) | 1 | (0.2%) |
|  |  | G1 | 0 | (0.0%) | 1 | (0.4%) | 1 | (0.2%) |
|  | VENOUS THROMBOTIC EVENT | Total | 1 | (0.4%) | 7 | (2.8%) | 8 | (1.6%) |
|  |  | G1 | 0 | (0.0%) | 1 | (0.4%) | 1 | (0.2%) |
|  |  | G2 | 1 | (0.4%) | 3 | (1.2%) | 4 | (0.8%) |
|  |  | G3/4/5 | 0 | (0.0%) | 3 | (1.2%) | 3 | (0.6%) |
|  |  |  |  |  |  |  |  |  |

| **Supplementary Table 2. Adverse events in the Aromatase Inhibitors subgroup (cont’d)** | | | | | | | | |
| --- | --- | --- | --- | --- | --- | --- | --- | --- |
|  |  | Maximum grade | Placebo | | Everolimus | | Safety analysis set | |
|  |  |  | N=384 | | N=374 | | N=758 | |
| Not coded | Total | Total | 3 | (0.8%) | 4 | (1.1%) | 7 | (0.9%) |
|  |  | G1 | 2 | (0.5%) | 1 | (0.3%) | 3 | (0.4%) |
|  |  | G2 | 1 | (0.3%) | 2 | (0.5%) | 3 | (0.4%) |
|  |  | G3/4/5 | 0 | (0.0%) | 1 | (0.3%) | 1 | (0.1%) |
|  | Not coded | Total | 3 | (0.8%) | 4 | (1.1%) | 7 | (0.9%) |
|  |  | G1 | 2 | (0.5%) | 1 | (0.3%) | 3 | (0.4%) |
|  |  | G2 | 1 | (0.3%) | 2 | (0.5%) | 3 | (0.4%) |
|  |  | G3/4/5 | 0 | (0.0%) | 1 | (0.3%) | 1 | (0.1%) |
| BLOOD AND LYMPHATIC SYSTEM DISORDERS | Total | Total | 17 | (4.4%) | 65 | (17.4%) | 82 | (10.8%) |
|  |  | G1 | 14 | (3.6%) | 58 | (15.5%) | 72 | (9.5%) |
|  |  | G2 | 3 | (0.8%) | 7 | (1.9%) | 10 | (1.3%) |
|  | ANEMIA | Total | 15 | (3.9%) | 63 | (16.8%) | 78 | (10.3%) |
|  |  | G1 | 13 | (3.4%) | 57 | (15.2%) | 70 | (9.2%) |
|  |  | G2 | 2 | (0.5%) | 6 | (1.6%) | 8 | (1.1%) |
|  | THROMBOSIS OF VENOUS SINUSES | Total | 1 | (0.3%) | 0 | (0.0%) | 1 | (0.1%) |
|  |  | G2 | 1 | (0.3%) | 0 | (0.0%) | 1 | (0.1%) |
|  | THROMBOTIC THROMBOCYTOPENIC PURPURA | Total | 1 | (0.3%) | 3 | (0.8%) | 4 | (0.5%) |
|  |  | G1 | 1 | (0.3%) | 2 | (0.5%) | 3 | (0.4%) |
|  |  | G2 | 0 | (0.0%) | 1 | (0.3%) | 1 | (0.1%) |
| CARDIAC DISORDERS | Total | Total | 22 | (5.7%) | 10 | (2.7%) | 32 | (4.2%) |
|  |  | G1 | 13 | (3.4%) | 6 | (1.6%) | 19 | (2.5%) |
|  |  | G2 | 5 | (1.3%) | 5 | (1.3%) | 10 | (1.3%) |
|  |  | G3/4/5 | 4 | (1.0%) | 1 | (0.3%) | 5 | (0.7%) |
|  | ATRIAL FIBRILLATION | Total | 0 | (0.0%) | 1 | (0.3%) | 1 | (0.1%) |
|  |  | G3/4/5 | 0 | (0.0%) | 1 | (0.3%) | 1 | (0.1%) |
|  | CHEST PAIN - CARDIAC | Total | 3 | (0.8%) | 1 | (0.3%) | 4 | (0.5%) |
|  |  | G1 | 2 | (0.5%) | 1 | (0.3%) | 3 | (0.4%) |
|  |  | G2 | 1 | (0.3%) | 0 | (0.0%) | 1 | (0.1%) |
|  | HEART FAILURE | Total | 1 | (0.3%) | 0 | (0.0%) | 1 | (0.1%) |
|  |  | G3/4/5 | 1 | (0.3%) | 0 | (0.0%) | 1 | (0.1%) |
|  | HEART MURMUR | Total | 1 | (0.3%) | 0 | (0.0%) | 1 | (0.1%) |
|  |  | G1 | 1 | (0.3%) | 0 | (0.0%) | 1 | (0.1%) |
|  | ISCHEMIC HEART DISEASE | Total | 0 | (0.0%) | 1 | (0.3%) | 1 | (0.1%) |
|  |  | G2 | 0 | (0.0%) | 1 | (0.3%) | 1 | (0.1%) |
|  | LEFT VENTRICULAR SYSTOLIC DYSFUNCTION | Total | 0 | (0.0%) | 1 | (0.3%) | 1 | (0.1%) |
|  |  | G1 | 0 | (0.0%) | 1 | (0.3%) | 1 | (0.1%) |
|  | MYOCARDIAL INFARCTION | Total | 2 | (0.5%) | 1 | (0.3%) | 3 | (0.4%) |
|  |  | G2 | 1 | (0.3%) | 1 | (0.3%) | 2 | (0.3%) |
|  |  | G3/4/5 | 1 | (0.3%) | 0 | (0.0%) | 1 | (0.1%) |
|  | MYOCARDITIS | Total | 1 | (0.3%) | 0 | (0.0%) | 1 | (0.1%) |
|  |  | G3/4/5 | 1 | (0.3%) | 0 | (0.0%) | 1 | (0.1%) |
|  | PALPITATIONS | Total | 10 | (2.6%) | 3 | (0.8%) | 13 | (1.7%) |
|  |  | G1 | 9 | (2.3%) | 3 | (0.8%) | 12 | (1.6%) |
|  |  | G2 | 1 | (0.3%) | 0 | (0.0%) | 1 | (0.1%) |
|  | PERICARDIAL EFFUSION | Total | 0 | (0.0%) | 1 | (0.3%) | 1 | (0.1%) |
|  |  | G2 | 0 | (0.0%) | 1 | (0.3%) | 1 | (0.1%) |
|  | PERICARDITIS | Total | 1 | (0.3%) | 0 | (0.0%) | 1 | (0.1%) |
|  |  | G3/4/5 | 1 | (0.3%) | 0 | (0.0%) | 1 | (0.1%) |
|  | SINUS TACHYCARDIA | Total | 1 | (0.3%) | 1 | (0.3%) | 2 | (0.3%) |
|  |  | G1 | 1 | (0.3%) | 0 | (0.0%) | 1 | (0.1%) |
|  |  | G2 | 0 | (0.0%) | 1 | (0.3%) | 1 | (0.1%) |
|  | TACHYCARDIA | Total | 2 | (0.5%) | 2 | (0.5%) | 4 | (0.5%) |
|  |  | G1 | 0 | (0.0%) | 1 | (0.3%) | 1 | (0.1%) |
|  |  | G2 | 2 | (0.5%) | 1 | (0.3%) | 3 | (0.4%) |
|  | VENTRICULAR TACHYCARDIA | Total | 0 | (0.0%) | 1 | (0.3%) | 1 | (0.1%) |
|  |  | G1 | 0 | (0.0%) | 1 | (0.3%) | 1 | (0.1%) |
| EAR AND LABYRINTH DISORDERS | Total | Total | 23 | (6.0%) | 14 | (3.7%) | 37 | (4.9%) |
|  |  | G1 | 19 | (4.9%) | 10 | (2.7%) | 29 | (3.8%) |
|  |  | G2 | 4 | (1.0%) | 4 | (1.1%) | 8 | (1.1%) |
|  | EAR PAIN | Total | 2 | (0.5%) | 1 | (0.3%) | 3 | (0.4%) |
|  |  | G1 | 1 | (0.3%) | 0 | (0.0%) | 1 | (0.1%) |
|  |  | G2 | 1 | (0.3%) | 1 | (0.3%) | 2 | (0.3%) |
|  | HEARING IMPAIRED | Total | 0 | (0.0%) | 1 | (0.3%) | 1 | (0.1%) |
|  |  | G1 | 0 | (0.0%) | 1 | (0.3%) | 1 | (0.1%) |
|  | MIDDLE EAR INFLAMMATION | Total | 2 | (0.5%) | 1 | (0.3%) | 3 | (0.4%) |
|  |  | G1 | 2 | (0.5%) | 0 | (0.0%) | 2 | (0.3%) |
|  |  | G2 | 0 | (0.0%) | 1 | (0.3%) | 1 | (0.1%) |
|  | TINNITUS | Total | 0 | (0.0%) | 2 | (0.5%) | 2 | (0.3%) |
|  |  | G1 | 0 | (0.0%) | 2 | (0.5%) | 2 | (0.3%) |
|  | VERTIGO | Total | 18 | (4.7%) | 10 | (2.7%) | 28 | (3.7%) |
|  |  | G1 | 16 | (4.2%) | 8 | (2.1%) | 24 | (3.2%) |
|  |  | G2 | 2 | (0.5%) | 2 | (0.5%) | 4 | (0.5%) |
|  | VESTIBULAR DISORDER | Total | 1 | (0.3%) | 0 | (0.0%) | 1 | (0.1%) |
|  |  | G2 | 1 | (0.3%) | 0 | (0.0%) | 1 | (0.1%) |
| ENDOCRINE DISORDERS | Total | Total | 3 | (0.8%) | 2 | (0.5%) | 5 | (0.7%) |
|  |  | . | 0 | (0.0%) | 1 | (0.3%) | 1 | (0.1%) |
|  |  | G1 | 2 | (0.5%) | 0 | (0.0%) | 2 | (0.3%) |
|  |  | G2 | 1 | (0.3%) | 1 | (0.3%) | 2 | (0.3%) |
|  | HYPOTHYROIDISM | Total | 3 | (0.8%) | 2 | (0.5%) | 5 | (0.7%) |
|  |  | . | 0 | (0.0%) | 1 | (0.3%) | 1 | (0.1%) |
|  |  | G1 | 2 | (0.5%) | 0 | (0.0%) | 2 | (0.3%) |
|  |  | G2 | 1 | (0.3%) | 1 | (0.3%) | 2 | (0.3%) |
| EYE DISORDERS | Total | Total | 19 | (4.9%) | 22 | (5.9%) | 41 | (5.4%) |
|  |  | G1 | 17 | (4.4%) | 15 | (4.0%) | 32 | (4.2%) |
|  |  | G2 | 2 | (0.5%) | 5 | (1.3%) | 7 | (0.9%) |
|  |  | G3/4/5 | 1 | (0.3%) | 2 | (0.5%) | 3 | (0.4%) |
|  | ACUITY REDUCED | Total | 2 | (0.5%) | 1 | (0.3%) | 3 | (0.4%) |
|  |  | G1 | 2 | (0.5%) | 0 | (0.0%) | 2 | (0.3%) |
|  |  | G2 | 0 | (0.0%) | 1 | (0.3%) | 1 | (0.1%) |
|  | BLURRED VISION | Total | 3 | (0.8%) | 4 | (1.1%) | 7 | (0.9%) |
|  |  | G1 | 3 | (0.8%) | 3 | (0.8%) | 6 | (0.8%) |
|  |  | G3/4/5 | 0 | (0.0%) | 1 | (0.3%) | 1 | (0.1%) |
|  | CATARACT | Total | 2 | (0.5%) | 0 | (0.0%) | 2 | (0.3%) |
|  |  | G1 | 1 | (0.3%) | 0 | (0.0%) | 1 | (0.1%) |
|  |  | G3/4/5 | 1 | (0.3%) | 0 | (0.0%) | 1 | (0.1%) |
|  | CHALAZION | Total | 1 | (0.3%) | 0 | (0.0%) | 1 | (0.1%) |
|  |  | G2 | 1 | (0.3%) | 0 | (0.0%) | 1 | (0.1%) |
|  | CONJUNCTIVITIS | Total | 3 | (0.8%) | 5 | (1.3%) | 8 | (1.1%) |
|  |  | G1 | 3 | (0.8%) | 2 | (0.5%) | 5 | (0.7%) |
|  |  | G2 | 0 | (0.0%) | 3 | (0.8%) | 3 | (0.4%) |
|  | DIPLOPIA | Total | 1 | (0.3%) | 0 | (0.0%) | 1 | (0.1%) |
|  |  | G1 | 1 | (0.3%) | 0 | (0.0%) | 1 | (0.1%) |
|  | DRY EYE | Total | 6 | (1.6%) | 8 | (2.1%) | 14 | (1.8%) |
|  |  | G1 | 5 | (1.3%) | 7 | (1.9%) | 12 | (1.6%) |
|  |  | G2 | 1 | (0.3%) | 1 | (0.3%) | 2 | (0.3%) |
|  | EYE BLEEDING | Total | 1 | (0.3%) | 0 | (0.0%) | 1 | (0.1%) |
|  |  | G1 | 1 | (0.3%) | 0 | (0.0%) | 1 | (0.1%) |
|  | EYE PAIN | Total | 0 | (0.0%) | 3 | (0.8%) | 3 | (0.4%) |
|  |  | G1 | 0 | (0.0%) | 3 | (0.8%) | 3 | (0.4%) |
|  | EYELID FUNCTION DISORDER | Total | 1 | (0.3%) | 1 | (0.3%) | 2 | (0.3%) |
|  |  | G1 | 1 | (0.3%) | 1 | (0.3%) | 2 | (0.3%) |
|  | RETINAL VASCULAR DISORDER | Total | 0 | (0.0%) | 1 | (0.3%) | 1 | (0.1%) |
|  |  | G3/4/5 | 0 | (0.0%) | 1 | (0.3%) | 1 | (0.1%) |
|  | WATERING EYES | Total | 2 | (0.5%) | 0 | (0.0%) | 2 | (0.3%) |
|  |  | G1 | 2 | (0.5%) | 0 | (0.0%) | 2 | (0.3%) |
| GASTROINTESTINAL DISORDERS | Total | Total | 223 | (58.1%) | 314 | (84.0%) | 537 | (70.8%) |
|  |  | . | 0 | (0.0%) | 3 | (0.8%) | 3 | (0.4%) |
|  |  | G1 | 200 | (52.1%) | 234 | (62.6%) | 434 | (57.3%) |
|  |  | G2 | 55 | (14.3%) | 140 | (37.4%) | 195 | (25.7%) |
|  |  | G3/4/5 | 6 | (1.6%) | 38 | (10.2%) | 44 | (5.8%) |
|  | ABDOMINAL DISTENSION | Total | 1 | (0.3%) | 0 | (0.0%) | 1 | (0.1%) |
|  |  | G2 | 1 | (0.3%) | 0 | (0.0%) | 1 | (0.1%) |
|  | ABDOMINAL PAIN | Total | 19 | (4.9%) | 25 | (6.7%) | 44 | (5.8%) |
|  |  | G1 | 15 | (3.9%) | 17 | (4.5%) | 32 | (4.2%) |
|  |  | G2 | 2 | (0.5%) | 7 | (1.9%) | 9 | (1.2%) |
|  |  | G3/4/5 | 2 | (0.5%) | 1 | (0.3%) | 3 | (0.4%) |
|  | ANAL PAIN | Total | 0 | (0.0%) | 1 | (0.3%) | 1 | (0.1%) |
|  |  | G1 | 0 | (0.0%) | 1 | (0.3%) | 1 | (0.1%) |
|  | ANAL ULCER | Total | 0 | (0.0%) | 1 | (0.3%) | 1 | (0.1%) |
|  |  | G1 | 0 | (0.0%) | 1 | (0.3%) | 1 | (0.1%) |
|  | ASCITES | Total | 0 | (0.0%) | 1 | (0.3%) | 1 | (0.1%) |
|  |  | G2 | 0 | (0.0%) | 1 | (0.3%) | 1 | (0.1%) |
|  | BLOATING | Total | 7 | (1.8%) | 4 | (1.1%) | 11 | (1.5%) |
|  |  | G1 | 6 | (1.6%) | 3 | (0.8%) | 9 | (1.2%) |
|  |  | G2 | 1 | (0.3%) | 1 | (0.3%) | 2 | (0.3%) |
|  | CHEILITIS | Total | 0 | (0.0%) | 1 | (0.3%) | 1 | (0.1%) |
|  |  | G1 | 0 | (0.0%) | 1 | (0.3%) | 1 | (0.1%) |
|  | COLITIS | Total | 1 | (0.3%) | 0 | (0.0%) | 1 | (0.1%) |
|  |  | G1 | 1 | (0.3%) | 0 | (0.0%) | 1 | (0.1%) |
|  | CONSTIPATION | Total | 22 | (5.7%) | 31 | (8.3%) | 53 | (7.0%) |
|  |  | G1 | 19 | (4.9%) | 27 | (7.2%) | 46 | (6.1%) |
|  |  | G2 | 3 | (0.8%) | 4 | (1.1%) | 7 | (0.9%) |
|  | DENTAL CARIES | Total | 0 | (0.0%) | 3 | (0.8%) | 3 | (0.4%) |
|  |  | G2 | 0 | (0.0%) | 2 | (0.5%) | 2 | (0.3%) |
|  |  | G3/4/5 | 0 | (0.0%) | 1 | (0.3%) | 1 | (0.1%) |
|  | DIARRHEA | Total | 77 | (20.1%) | 90 | (24.1%) | 167 | (22.0%) |
|  |  | G1 | 64 | (16.7%) | 69 | (18.4%) | 133 | (17.5%) |
|  |  | G2 | 13 | (3.4%) | 18 | (4.8%) | 31 | (4.1%) |
|  |  | G3/4/5 | 0 | (0.0%) | 3 | (0.8%) | 3 | (0.4%) |
|  | DISCOLORED STOOLS | Total | 0 | (0.0%) | 1 | (0.3%) | 1 | (0.1%) |
|  |  | G1 | 0 | (0.0%) | 1 | (0.3%) | 1 | (0.1%) |
|  | DRY MOUTH | Total | 22 | (5.7%) | 27 | (7.2%) | 49 | (6.5%) |
|  |  | G1 | 21 | (5.5%) | 24 | (6.4%) | 45 | (5.9%) |
|  |  | G2 | 1 | (0.3%) | 3 | (0.8%) | 4 | (0.5%) |
|  | DYSPEPSIA | Total | 7 | (1.8%) | 7 | (1.9%) | 14 | (1.8%) |
|  |  | G1 | 7 | (1.8%) | 5 | (1.3%) | 12 | (1.6%) |
|  |  | G2 | 0 | (0.0%) | 2 | (0.5%) | 2 | (0.3%) |
|  | DYSPHAGIA | Total | 1 | (0.3%) | 4 | (1.1%) | 5 | (0.7%) |
|  |  | G1 | 1 | (0.3%) | 3 | (0.8%) | 4 | (0.5%) |
|  |  | G3/4/5 | 0 | (0.0%) | 1 | (0.3%) | 1 | (0.1%) |
|  | ESOPHAGITIS | Total | 1 | (0.3%) | 0 | (0.0%) | 1 | (0.1%) |
|  |  | G2 | 1 | (0.3%) | 0 | (0.0%) | 1 | (0.1%) |
|  | FLATULENCE | Total | 0 | (0.0%) | 2 | (0.5%) | 2 | (0.3%) |
|  |  | G1 | 0 | (0.0%) | 2 | (0.5%) | 2 | (0.3%) |
|  | GASTRITIS | Total | 7 | (1.8%) | 5 | (1.3%) | 12 | (1.6%) |
|  |  | G1 | 5 | (1.3%) | 3 | (0.8%) | 8 | (1.1%) |
|  |  | G2 | 2 | (0.5%) | 2 | (0.5%) | 4 | (0.5%) |
|  | GASTROESOPHAGEAL REFLUX DISEASE | Total | 11 | (2.9%) | 7 | (1.9%) | 18 | (2.4%) |
|  |  | G1 | 6 | (1.6%) | 6 | (1.6%) | 12 | (1.6%) |
|  |  | G2 | 5 | (1.3%) | 1 | (0.3%) | 6 | (0.8%) |
|  | GASTROINTESTINAL DISORDERS - OTHER, SPECIFY | Total | 0 | (0.0%) | 1 | (0.3%) | 1 | (0.1%) |
|  |  | G3/4/5 | 0 | (0.0%) | 1 | (0.3%) | 1 | (0.1%) |
|  | GASTROINTESTINAL PAIN | Total | 9 | (2.3%) | 8 | (2.1%) | 17 | (2.2%) |
|  |  | G1 | 6 | (1.6%) | 7 | (1.9%) | 13 | (1.7%) |
|  |  | G2 | 2 | (0.5%) | 1 | (0.3%) | 3 | (0.4%) |
|  |  | G3/4/5 | 1 | (0.3%) | 0 | (0.0%) | 1 | (0.1%) |
|  | GINGIVAL PAIN | Total | 5 | (1.3%) | 7 | (1.9%) | 12 | (1.6%) |
|  |  | G1 | 2 | (0.5%) | 5 | (1.3%) | 7 | (0.9%) |
|  |  | G2 | 2 | (0.5%) | 1 | (0.3%) | 3 | (0.4%) |
|  |  | G3/4/5 | 1 | (0.3%) | 1 | (0.3%) | 2 | (0.3%) |
|  | HEMORRHOIDS | Total | 3 | (0.8%) | 5 | (1.3%) | 8 | (1.1%) |
|  |  | G1 | 0 | (0.0%) | 3 | (0.8%) | 3 | (0.4%) |
|  |  | G2 | 3 | (0.8%) | 1 | (0.3%) | 4 | (0.5%) |
|  |  | G3/4/5 | 0 | (0.0%) | 1 | (0.3%) | 1 | (0.1%) |
|  | INTERMITTENT FEELING OF HUNGER | Total | 0 | (0.0%) | 1 | (0.3%) | 1 | (0.1%) |
|  |  | G1 | 0 | (0.0%) | 1 | (0.3%) | 1 | (0.1%) |
|  | LIP PAIN | Total | 0 | (0.0%) | 2 | (0.5%) | 2 | (0.3%) |
|  |  | G1 | 0 | (0.0%) | 1 | (0.3%) | 1 | (0.1%) |
|  |  | G2 | 0 | (0.0%) | 1 | (0.3%) | 1 | (0.1%) |
|  | MUCOSITIS ORAL | Total | 103 | (26.8%) | 241 | (64.4%) | 344 | (45.4%) |
|  |  | . | 0 | (0.0%) | 1 | (0.3%) | 1 | (0.1%) |
|  |  | G1 | 89 | (23.2%) | 112 | (29.9%) | 201 | (26.5%) |
|  |  | G2 | 13 | (3.4%) | 101 | (27.0%) | 114 | (15.0%) |
|  |  | G3/4/5 | 1 | (0.3%) | 27 | (7.2%) | 28 | (3.7%) |
|  | NAUSEA | Total | 77 | (20.1%) | 83 | (22.2%) | 160 | (21.1%) |
|  |  | . | 0 | (0.0%) | 1 | (0.3%) | 1 | (0.1%) |
|  |  | G1 | 67 | (17.4%) | 68 | (18.2%) | 135 | (17.8%) |
|  |  | G2 | 9 | (2.3%) | 13 | (3.5%) | 22 | (2.9%) |
|  |  | G3/4/5 | 1 | (0.3%) | 1 | (0.3%) | 2 | (0.3%) |
|  | OBSTRUCTION GASTRIC | Total | 0 | (0.0%) | 1 | (0.3%) | 1 | (0.1%) |
|  |  | G3/4/5 | 0 | (0.0%) | 1 | (0.3%) | 1 | (0.1%) |
|  | ORAL DYSESTHESIA | Total | 1 | (0.3%) | 0 | (0.0%) | 1 | (0.1%) |
|  |  | G1 | 1 | (0.3%) | 0 | (0.0%) | 1 | (0.1%) |
|  | ORAL PAIN | Total | 5 | (1.3%) | 5 | (1.3%) | 10 | (1.3%) |
|  |  | G1 | 5 | (1.3%) | 3 | (0.8%) | 8 | (1.1%) |
|  |  | G2 | 0 | (0.0%) | 1 | (0.3%) | 1 | (0.1%) |
|  |  | G3/4/5 | 0 | (0.0%) | 1 | (0.3%) | 1 | (0.1%) |
|  | PROCTITIS | Total | 1 | (0.3%) | 0 | (0.0%) | 1 | (0.1%) |
|  |  | G2 | 1 | (0.3%) | 0 | (0.0%) | 1 | (0.1%) |
|  | RECTAL HEMORRHAGE | Total | 0 | (0.0%) | 1 | (0.3%) | 1 | (0.1%) |
|  |  | G1 | 0 | (0.0%) | 1 | (0.3%) | 1 | (0.1%) |
|  | RUMBLING | Total | 1 | (0.3%) | 0 | (0.0%) | 1 | (0.1%) |
|  |  | G1 | 1 | (0.3%) | 0 | (0.0%) | 1 | (0.1%) |
|  | SALIVARY DUCT INFLAMMATION | Total | 1 | (0.3%) | 0 | (0.0%) | 1 | (0.1%) |
|  |  | G1 | 1 | (0.3%) | 0 | (0.0%) | 1 | (0.1%) |
|  | STOMACH PAIN | Total | 5 | (1.3%) | 7 | (1.9%) | 12 | (1.6%) |
|  |  | G1 | 5 | (1.3%) | 6 | (1.6%) | 11 | (1.5%) |
|  |  | G2 | 0 | (0.0%) | 1 | (0.3%) | 1 | (0.1%) |
|  | TOOTH DEVELOPMENT DISORDER | Total | 0 | (0.0%) | 1 | (0.3%) | 1 | (0.1%) |
|  |  | G1 | 0 | (0.0%) | 1 | (0.3%) | 1 | (0.1%) |
|  | TOOTH DISCOLORATION | Total | 1 | (0.3%) | 0 | (0.0%) | 1 | (0.1%) |
|  |  | G1 | 1 | (0.3%) | 0 | (0.0%) | 1 | (0.1%) |
|  | TOOTHACHE | Total | 2 | (0.5%) | 2 | (0.5%) | 4 | (0.5%) |
|  |  | G1 | 2 | (0.5%) | 0 | (0.0%) | 2 | (0.3%) |
|  |  | G2 | 0 | (0.0%) | 2 | (0.5%) | 2 | (0.3%) |
|  | VOMITING | Total | 21 | (5.5%) | 23 | (6.1%) | 44 | (5.8%) |
|  |  | . | 0 | (0.0%) | 1 | (0.3%) | 1 | (0.1%) |
|  |  | G1 | 18 | (4.7%) | 15 | (4.0%) | 33 | (4.4%) |
|  |  | G2 | 3 | (0.8%) | 6 | (1.6%) | 9 | (1.2%) |
|  |  | G3/4/5 | 0 | (0.0%) | 1 | (0.3%) | 1 | (0.1%) |
| GENERAL DISORDERS AND ADMINISTRATION SITE CONDITIONS | Total | Total | 230 | (59.9%) | 264 | (70.6%) | 494 | (65.2%) |
|  |  | . | 4 | (1.0%) | 2 | (0.5%) | 6 | (0.8%) |
|  |  | G1 | 186 | (48.4%) | 194 | (51.9%) | 380 | (50.1%) |
|  |  | G2 | 65 | (16.9%) | 102 | (27.3%) | 167 | (22.0%) |
|  |  | G3/4/5 | 4 | (1.0%) | 12 | (3.2%) | 16 | (2.1%) |
|  | CHILLS | Total | 1 | (0.3%) | 10 | (2.7%) | 11 | (1.5%) |
|  |  | G1 | 1 | (0.3%) | 10 | (2.7%) | 11 | (1.5%) |
|  | EDEMA FACE | Total | 0 | (0.0%) | 9 | (2.4%) | 9 | (1.2%) |
|  |  | G1 | 0 | (0.0%) | 8 | (2.1%) | 8 | (1.1%) |
|  |  | G2 | 0 | (0.0%) | 1 | (0.3%) | 1 | (0.1%) |
|  | EDEMA LIMBS | Total | 12 | (3.1%) | 47 | (12.6%) | 59 | (7.8%) |
|  |  | . | 1 | (0.3%) | 0 | (0.0%) | 1 | (0.1%) |
|  |  | G1 | 10 | (2.6%) | 35 | (9.4%) | 45 | (5.9%) |
|  |  | G2 | 1 | (0.3%) | 12 | (3.2%) | 13 | (1.7%) |
|  | FATIGUE | Total | 190 | (49.5%) | 201 | (53.7%) | 391 | (51.6%) |
|  |  | . | 1 | (0.3%) | 0 | (0.0%) | 1 | (0.1%) |
|  |  | G1 | 134 | (34.9%) | 117 | (31.3%) | 251 | (33.1%) |
|  |  | G2 | 51 | (13.3%) | 75 | (20.1%) | 126 | (16.6%) |
|  |  | G3/4/5 | 4 | (1.0%) | 9 | (2.4%) | 13 | (1.7%) |
|  | FEVER | Total | 20 | (5.2%) | 36 | (9.6%) | 56 | (7.4%) |
|  |  | G1 | 15 | (3.9%) | 28 | (7.5%) | 43 | (5.7%) |
|  |  | G2 | 5 | (1.3%) | 7 | (1.9%) | 12 | (1.6%) |
|  |  | G3/4/5 | 0 | (0.0%) | 1 | (0.3%) | 1 | (0.1%) |
|  | FLU LIKE SYMPTOMS | Total | 30 | (7.8%) | 23 | (6.1%) | 53 | (7.0%) |
|  |  | . | 1 | (0.3%) | 0 | (0.0%) | 1 | (0.1%) |
|  |  | G1 | 21 | (5.5%) | 15 | (4.0%) | 36 | (4.7%) |
|  |  | G2 | 8 | (2.1%) | 8 | (2.1%) | 16 | (2.1%) |
|  | GAIT DISTURBANCE | Total | 0 | (0.0%) | 1 | (0.3%) | 1 | (0.1%) |
|  |  | G1 | 0 | (0.0%) | 1 | (0.3%) | 1 | (0.1%) |
|  | IMPAIRED HEALING | Total | 0 | (0.0%) | 1 | (0.3%) | 1 | (0.1%) |
|  |  | G1 | 0 | (0.0%) | 1 | (0.3%) | 1 | (0.1%) |
|  | IRRITABILITY | Total | 3 | (0.8%) | 1 | (0.3%) | 4 | (0.5%) |
|  |  | G1 | 2 | (0.5%) | 1 | (0.3%) | 3 | (0.4%) |
|  |  | G2 | 1 | (0.3%) | 0 | (0.0%) | 1 | (0.1%) |
|  | LEFT UNDER MANDIBULAR SWELLING | Total | 0 | (0.0%) | 1 | (0.3%) | 1 | (0.1%) |
|  |  | G1 | 0 | (0.0%) | 1 | (0.3%) | 1 | (0.1%) |
|  | LOCALIZED EDEMA | Total | 7 | (1.8%) | 23 | (6.1%) | 30 | (4.0%) |
|  |  | . | 0 | (0.0%) | 1 | (0.3%) | 1 | (0.1%) |
|  |  | G1 | 6 | (1.6%) | 19 | (5.1%) | 25 | (3.3%) |
|  |  | G2 | 1 | (0.3%) | 2 | (0.5%) | 3 | (0.4%) |
|  |  | G3/4/5 | 0 | (0.0%) | 1 | (0.3%) | 1 | (0.1%) |
|  | MALAISE | Total | 6 | (1.6%) | 3 | (0.8%) | 9 | (1.2%) |
|  |  | G1 | 5 | (1.3%) | 3 | (0.8%) | 8 | (1.1%) |
|  |  | G2 | 1 | (0.3%) | 0 | (0.0%) | 1 | (0.1%) |
|  | MOBILITY DECREASED | Total | 1 | (0.3%) | 0 | (0.0%) | 1 | (0.1%) |
|  |  | G2 | 1 | (0.3%) | 0 | (0.0%) | 1 | (0.1%) |
|  | NON-CARDIAC CHEST PAIN | Total | 1 | (0.3%) | 0 | (0.0%) | 1 | (0.1%) |
|  |  | G2 | 1 | (0.3%) | 0 | (0.0%) | 1 | (0.1%) |
|  | PAIN | Total | 45 | (11.7%) | 47 | (12.6%) | 92 | (12.1%) |
|  |  | . | 0 | (0.0%) | 1 | (0.3%) | 1 | (0.1%) |
|  |  | G1 | 38 | (9.9%) | 38 | (10.2%) | 76 | (10.0%) |
|  |  | G2 | 7 | (1.8%) | 7 | (1.9%) | 14 | (1.8%) |
|  |  | G3/4/5 | 0 | (0.0%) | 1 | (0.3%) | 1 | (0.1%) |
|  | POLYDIPSIA | Total | 1 | (0.3%) | 0 | (0.0%) | 1 | (0.1%) |
|  |  | G1 | 1 | (0.3%) | 0 | (0.0%) | 1 | (0.1%) |
|  | ROTATOR CUFF INJURY | Total | 1 | (0.3%) | 0 | (0.0%) | 1 | (0.1%) |
|  |  | G1 | 1 | (0.3%) | 0 | (0.0%) | 1 | (0.1%) |
|  | SENSATION OF HEAVINESS | Total | 1 | (0.3%) | 0 | (0.0%) | 1 | (0.1%) |
|  |  | . | 1 | (0.3%) | 0 | (0.0%) | 1 | (0.1%) |
|  | TINGLING RIGHT SIDE FACE AND LEG | Total | 0 | (0.0%) | 1 | (0.3%) | 1 | (0.1%) |
|  |  | G1 | 0 | (0.0%) | 1 | (0.3%) | 1 | (0.1%) |
|  | TRAPPED WIND | Total | 1 | (0.3%) | 0 | (0.0%) | 1 | (0.1%) |
|  |  | G1 | 1 | (0.3%) | 0 | (0.0%) | 1 | (0.1%) |
| HEPATOBILIARY DISORDERS | Total | Total | 2 | (0.5%) | 8 | (2.1%) | 10 | (1.3%) |
|  |  | G1 | 2 | (0.5%) | 5 | (1.3%) | 7 | (0.9%) |
|  |  | G2 | 0 | (0.0%) | 2 | (0.5%) | 2 | (0.3%) |
|  |  | G3/4/5 | 0 | (0.0%) | 1 | (0.3%) | 1 | (0.1%) |
|  | BILE DUCT STENOSIS | Total | 0 | (0.0%) | 1 | (0.3%) | 1 | (0.1%) |
|  |  | G2 | 0 | (0.0%) | 1 | (0.3%) | 1 | (0.1%) |
|  | BLOOD ALKALINE PHOSPHATASE DECREASED | Total | 1 | (0.3%) | 1 | (0.3%) | 2 | (0.3%) |
|  |  | G1 | 1 | (0.3%) | 1 | (0.3%) | 2 | (0.3%) |
|  | CHOLECYSTITIS | Total | 0 | (0.0%) | 1 | (0.3%) | 1 | (0.1%) |
|  |  | G1 | 0 | (0.0%) | 1 | (0.3%) | 1 | (0.1%) |
|  | CHOLELITHIASIS | Total | 0 | (0.0%) | 1 | (0.3%) | 1 | (0.1%) |
|  |  | G2 | 0 | (0.0%) | 1 | (0.3%) | 1 | (0.1%) |
|  | CHOLESTASIS | Total | 0 | (0.0%) | 1 | (0.3%) | 1 | (0.1%) |
|  |  | G1 | 0 | (0.0%) | 1 | (0.3%) | 1 | (0.1%) |
|  | HEPATIC FAILURE | Total | 1 | (0.3%) | 3 | (0.8%) | 4 | (0.5%) |
|  |  | G1 | 1 | (0.3%) | 3 | (0.8%) | 4 | (0.5%) |
|  | HEPATIC PAIN | Total | 0 | (0.0%) | 2 | (0.5%) | 2 | (0.3%) |
|  |  | G1 | 0 | (0.0%) | 1 | (0.3%) | 1 | (0.1%) |
|  |  | G3/4/5 | 0 | (0.0%) | 1 | (0.3%) | 1 | (0.1%) |
| IMMUNE SYSTEM DISORDERS | Total | Total | 1 | (0.3%) | 3 | (0.8%) | 4 | (0.5%) |
|  |  | G1 | 1 | (0.3%) | 2 | (0.5%) | 3 | (0.4%) |
|  |  | G2 | 0 | (0.0%) | 1 | (0.3%) | 1 | (0.1%) |
|  | ALLERGIC REACTION | Total | 1 | (0.3%) | 3 | (0.8%) | 4 | (0.5%) |
|  |  | G1 | 1 | (0.3%) | 2 | (0.5%) | 3 | (0.4%) |
|  |  | G2 | 0 | (0.0%) | 1 | (0.3%) | 1 | (0.1%) |
| INFECTIONS AND INFESTATIONS | Total | Total | 123 | (32.0%) | 172 | (46.0%) | 295 | (38.9%) |
|  |  | . | 3 | (0.8%) | 3 | (0.8%) | 6 | (0.8%) |
|  |  | G1 | 71 | (18.5%) | 113 | (30.2%) | 184 | (24.3%) |
|  |  | G2 | 60 | (15.6%) | 73 | (19.5%) | 133 | (17.5%) |
|  |  | G3/4/5 | 7 | (1.8%) | 11 | (2.9%) | 18 | (2.4%) |
|  | ABDOMINAL INFECTION | Total | 1 | (0.3%) | 0 | (0.0%) | 1 | (0.1%) |
|  |  | G1 | 1 | (0.3%) | 0 | (0.0%) | 1 | (0.1%) |
|  | BILIARY TRACT INFECTION | Total | 1 | (0.3%) | 0 | (0.0%) | 1 | (0.1%) |
|  |  | G3/4/5 | 1 | (0.3%) | 0 | (0.0%) | 1 | (0.1%) |
|  | BONE INFECTION | Total | 0 | (0.0%) | 1 | (0.3%) | 1 | (0.1%) |
|  |  | G3/4/5 | 0 | (0.0%) | 1 | (0.3%) | 1 | (0.1%) |
|  | BREAST INFECTION | Total | 1 | (0.3%) | 6 | (1.6%) | 7 | (0.9%) |
|  |  | . | 0 | (0.0%) | 1 | (0.3%) | 1 | (0.1%) |
|  |  | G1 | 0 | (0.0%) | 3 | (0.8%) | 3 | (0.4%) |
|  |  | G2 | 1 | (0.3%) | 1 | (0.3%) | 2 | (0.3%) |
|  |  | G3/4/5 | 0 | (0.0%) | 1 | (0.3%) | 1 | (0.1%) |
|  | BRONCHIAL INFECTION | Total | 31 | (8.1%) | 16 | (4.3%) | 47 | (6.2%) |
|  |  | G1 | 15 | (3.9%) | 11 | (2.9%) | 26 | (3.4%) |
|  |  | G2 | 16 | (4.2%) | 5 | (1.3%) | 21 | (2.8%) |
|  | CECAL INFECTION | Total | 0 | (0.0%) | 2 | (0.5%) | 2 | (0.3%) |
|  |  | G2 | 0 | (0.0%) | 2 | (0.5%) | 2 | (0.3%) |
|  | CORNEAL INFECTION | Total | 0 | (0.0%) | 1 | (0.3%) | 1 | (0.1%) |
|  |  | G2 | 0 | (0.0%) | 1 | (0.3%) | 1 | (0.1%) |
|  | DUODENAL INFECTION | Total | 1 | (0.3%) | 0 | (0.0%) | 1 | (0.1%) |
|  |  | G1 | 1 | (0.3%) | 0 | (0.0%) | 1 | (0.1%) |
|  | ENTEROCOLITIS INFECTIOUS | Total | 5 | (1.3%) | 5 | (1.3%) | 10 | (1.3%) |
|  |  | G1 | 2 | (0.5%) | 3 | (0.8%) | 5 | (0.7%) |
|  |  | G2 | 3 | (0.8%) | 1 | (0.3%) | 4 | (0.5%) |
|  |  | G3/4/5 | 0 | (0.0%) | 1 | (0.3%) | 1 | (0.1%) |
|  | EYE INFECTION | Total | 1 | (0.3%) | 1 | (0.3%) | 2 | (0.3%) |
|  |  | G1 | 1 | (0.3%) | 0 | (0.0%) | 1 | (0.1%) |
|  |  | G2 | 0 | (0.0%) | 1 | (0.3%) | 1 | (0.1%) |
|  | FINGER INFECTION | Total | 0 | (0.0%) | 1 | (0.3%) | 1 | (0.1%) |
|  |  | G1 | 0 | (0.0%) | 1 | (0.3%) | 1 | (0.1%) |
|  | FURUNCLE | Total | 0 | (0.0%) | 1 | (0.3%) | 1 | (0.1%) |
|  |  | G1 | 0 | (0.0%) | 1 | (0.3%) | 1 | (0.1%) |
|  | HEPATIC INFECTION | Total | 0 | (0.0%) | 3 | (0.8%) | 3 | (0.4%) |
|  |  | G1 | 0 | (0.0%) | 1 | (0.3%) | 1 | (0.1%) |
|  |  | G3/4/5 | 0 | (0.0%) | 2 | (0.5%) | 2 | (0.3%) |
|  | HERPES | Total | 1 | (0.3%) | 1 | (0.3%) | 2 | (0.3%) |
|  |  | G1 | 0 | (0.0%) | 1 | (0.3%) | 1 | (0.1%) |
|  |  | G2 | 1 | (0.3%) | 0 | (0.0%) | 1 | (0.1%) |
|  | INFLAMMED MIDDLE FINGER | Total | 0 | (0.0%) | 1 | (0.3%) | 1 | (0.1%) |
|  |  | G2 | 0 | (0.0%) | 1 | (0.3%) | 1 | (0.1%) |
|  | JOINT INFECTION | Total | 0 | (0.0%) | 2 | (0.5%) | 2 | (0.3%) |
|  |  | G2 | 0 | (0.0%) | 2 | (0.5%) | 2 | (0.3%) |
|  | LARYNGITIS | Total | 3 | (0.8%) | 0 | (0.0%) | 3 | (0.4%) |
|  |  | G2 | 2 | (0.5%) | 0 | (0.0%) | 2 | (0.3%) |
|  |  | G3/4/5 | 1 | (0.3%) | 0 | (0.0%) | 1 | (0.1%) |
|  | LIP INFECTION | Total | 11 | (2.9%) | 11 | (2.9%) | 22 | (2.9%) |
|  |  | G1 | 7 | (1.8%) | 10 | (2.7%) | 17 | (2.2%) |
|  |  | G2 | 4 | (1.0%) | 1 | (0.3%) | 5 | (0.7%) |
|  | LOCALIZED | Total | 3 | (0.8%) | 3 | (0.8%) | 6 | (0.8%) |
|  |  | G1 | 2 | (0.5%) | 2 | (0.5%) | 4 | (0.5%) |
|  |  | G2 | 0 | (0.0%) | 1 | (0.3%) | 1 | (0.1%) |
|  |  | G3/4/5 | 1 | (0.3%) | 0 | (0.0%) | 1 | (0.1%) |
|  | LUNG INFECTION | Total | 1 | (0.3%) | 5 | (1.3%) | 6 | (0.8%) |
|  |  | G1 | 0 | (0.0%) | 2 | (0.5%) | 2 | (0.3%) |
|  |  | G2 | 1 | (0.3%) | 1 | (0.3%) | 2 | (0.3%) |
|  |  | G3/4/5 | 0 | (0.0%) | 2 | (0.5%) | 2 | (0.3%) |
|  | LYMPH GLAND INFECTION | Total | 2 | (0.5%) | 2 | (0.5%) | 4 | (0.5%) |
|  |  | G1 | 0 | (0.0%) | 1 | (0.3%) | 1 | (0.1%) |
|  |  | G2 | 1 | (0.3%) | 1 | (0.3%) | 2 | (0.3%) |
|  |  | G3/4/5 | 1 | (0.3%) | 0 | (0.0%) | 1 | (0.1%) |
|  | MUCOSAL INFECTION | Total | 7 | (1.8%) | 13 | (3.5%) | 20 | (2.6%) |
|  |  | . | 0 | (0.0%) | 1 | (0.3%) | 1 | (0.1%) |
|  |  | G1 | 5 | (1.3%) | 6 | (1.6%) | 11 | (1.5%) |
|  |  | G2 | 1 | (0.3%) | 6 | (1.6%) | 7 | (0.9%) |
|  |  | G3/4/5 | 1 | (0.3%) | 0 | (0.0%) | 1 | (0.1%) |
|  | MYCOSIS | Total | 2 | (0.5%) | 3 | (0.8%) | 5 | (0.7%) |
|  |  | G1 | 0 | (0.0%) | 2 | (0.5%) | 2 | (0.3%) |
|  |  | G2 | 2 | (0.5%) | 1 | (0.3%) | 3 | (0.4%) |
|  | NAIL INFECTION | Total | 1 | (0.3%) | 7 | (1.9%) | 8 | (1.1%) |
|  |  | G1 | 0 | (0.0%) | 6 | (1.6%) | 6 | (0.8%) |
|  |  | G2 | 1 | (0.3%) | 1 | (0.3%) | 2 | (0.3%) |
|  | NASAL HERPES | Total | 0 | (0.0%) | 1 | (0.3%) | 1 | (0.1%) |
|  |  | G1 | 0 | (0.0%) | 1 | (0.3%) | 1 | (0.1%) |
|  | OTITIS EXTERNA | Total | 3 | (0.8%) | 3 | (0.8%) | 6 | (0.8%) |
|  |  | . | 1 | (0.3%) | 0 | (0.0%) | 1 | (0.1%) |
|  |  | G1 | 1 | (0.3%) | 1 | (0.3%) | 2 | (0.3%) |
|  |  | G2 | 1 | (0.3%) | 2 | (0.5%) | 3 | (0.4%) |
|  | PANCREAS INFECTION | Total | 0 | (0.0%) | 1 | (0.3%) | 1 | (0.1%) |
|  |  | G3/4/5 | 0 | (0.0%) | 1 | (0.3%) | 1 | (0.1%) |
|  | PAPULOPUSTULAR RASH | Total | 1 | (0.3%) | 3 | (0.8%) | 4 | (0.5%) |
|  |  | G1 | 0 | (0.0%) | 2 | (0.5%) | 2 | (0.3%) |
|  |  | G2 | 1 | (0.3%) | 0 | (0.0%) | 1 | (0.1%) |
|  |  | G3/4/5 | 0 | (0.0%) | 1 | (0.3%) | 1 | (0.1%) |
|  | PHARYNGITIS | Total | 8 | (2.1%) | 10 | (2.7%) | 18 | (2.4%) |
|  |  | . | 0 | (0.0%) | 1 | (0.3%) | 1 | (0.1%) |
|  |  | G1 | 4 | (1.0%) | 4 | (1.1%) | 8 | (1.1%) |
|  |  | G2 | 4 | (1.0%) | 5 | (1.3%) | 9 | (1.2%) |
|  | PLEURAL INFECTION | Total | 0 | (0.0%) | 1 | (0.3%) | 1 | (0.1%) |
|  |  | G1 | 0 | (0.0%) | 1 | (0.3%) | 1 | (0.1%) |
|  | RASH PUSTULAR | Total | 1 | (0.3%) | 1 | (0.3%) | 2 | (0.3%) |
|  |  | G1 | 1 | (0.3%) | 1 | (0.3%) | 2 | (0.3%) |
|  | RHINITIS | Total | 2 | (0.5%) | 8 | (2.1%) | 10 | (1.3%) |
|  |  | G1 | 2 | (0.5%) | 7 | (1.9%) | 9 | (1.2%) |
|  |  | G2 | 0 | (0.0%) | 1 | (0.3%) | 1 | (0.1%) |
|  | RHINITIS INFECTIVE | Total | 14 | (3.6%) | 8 | (2.1%) | 22 | (2.9%) |
|  |  | G1 | 9 | (2.3%) | 7 | (1.9%) | 16 | (2.1%) |
|  |  | G2 | 5 | (1.3%) | 1 | (0.3%) | 6 | (0.8%) |
|  | RHINOPHARYNGITIS | Total | 4 | (1.0%) | 5 | (1.3%) | 9 | (1.2%) |
|  |  | G1 | 1 | (0.3%) | 3 | (0.8%) | 4 | (0.5%) |
|  |  | G2 | 3 | (0.8%) | 2 | (0.5%) | 5 | (0.7%) |
|  | SCLERODERMA | Total | 1 | (0.3%) | 0 | (0.0%) | 1 | (0.1%) |
|  |  | G3/4/5 | 1 | (0.3%) | 0 | (0.0%) | 1 | (0.1%) |
|  | SEPSIS | Total | 1 | (0.3%) | 1 | (0.3%) | 2 | (0.3%) |
|  |  | G2 | 1 | (0.3%) | 0 | (0.0%) | 1 | (0.1%) |
|  |  | G3/4/5 | 0 | (0.0%) | 1 | (0.3%) | 1 | (0.1%) |
|  | SINUSITIS | Total | 9 | (2.3%) | 5 | (1.3%) | 14 | (1.8%) |
|  |  | G1 | 6 | (1.6%) | 2 | (0.5%) | 8 | (1.1%) |
|  |  | G2 | 3 | (0.8%) | 3 | (0.8%) | 6 | (0.8%) |
|  | SKIN INFECTION | Total | 18 | (4.7%) | 44 | (11.8%) | 62 | (8.2%) |
|  |  | . | 1 | (0.3%) | 0 | (0.0%) | 1 | (0.1%) |
|  |  | G1 | 15 | (3.9%) | 32 | (8.6%) | 47 | (6.2%) |
|  |  | G2 | 2 | (0.5%) | 11 | (2.9%) | 13 | (1.7%) |
|  |  | G3/4/5 | 0 | (0.0%) | 1 | (0.3%) | 1 | (0.1%) |
|  | STAPHYLOCOCCUS AUREUS INFECTION | Total | 0 | (0.0%) | 2 | (0.5%) | 2 | (0.3%) |
|  |  | G2 | 0 | (0.0%) | 2 | (0.5%) | 2 | (0.3%) |
|  | STAPHYLOCOCCUS EIDERMIDIS SEPTICEMIA | Total | 1 | (0.3%) | 0 | (0.0%) | 1 | (0.1%) |
|  |  | G2 | 1 | (0.3%) | 0 | (0.0%) | 1 | (0.1%) |
|  | STOMA SITE INFECTION | Total | 1 | (0.3%) | 0 | (0.0%) | 1 | (0.1%) |
|  |  | G1 | 1 | (0.3%) | 0 | (0.0%) | 1 | (0.1%) |
|  | TOOTH INFECTION | Total | 5 | (1.3%) | 13 | (3.5%) | 18 | (2.4%) |
|  |  | G1 | 3 | (0.8%) | 4 | (1.1%) | 7 | (0.9%) |
|  |  | G2 | 1 | (0.3%) | 9 | (2.4%) | 10 | (1.3%) |
|  |  | G3/4/5 | 1 | (0.3%) | 0 | (0.0%) | 1 | (0.1%) |
|  | TRACHEITIS | Total | 3 | (0.8%) | 2 | (0.5%) | 5 | (0.7%) |
|  |  | G1 | 1 | (0.3%) | 1 | (0.3%) | 2 | (0.3%) |
|  |  | G2 | 2 | (0.5%) | 1 | (0.3%) | 3 | (0.4%) |
|  | UPPER RESPIRATORY INFECTION | Total | 4 | (1.0%) | 8 | (2.1%) | 12 | (1.6%) |
|  |  | G1 | 4 | (1.0%) | 3 | (0.8%) | 7 | (0.9%) |
|  |  | G2 | 0 | (0.0%) | 5 | (1.3%) | 5 | (0.7%) |
|  | URINARY TRACT INFECTION | Total | 15 | (3.9%) | 19 | (5.1%) | 34 | (4.5%) |
|  |  | . | 1 | (0.3%) | 0 | (0.0%) | 1 | (0.1%) |
|  |  | G1 | 6 | (1.6%) | 9 | (2.4%) | 15 | (2.0%) |
|  |  | G2 | 8 | (2.1%) | 10 | (2.7%) | 18 | (2.4%) |
|  | VAGINAL INFECTION | Total | 2 | (0.5%) | 7 | (1.9%) | 9 | (1.2%) |
|  |  | G1 | 2 | (0.5%) | 5 | (1.3%) | 7 | (0.9%) |
|  |  | G2 | 0 | (0.0%) | 2 | (0.5%) | 2 | (0.3%) |
|  | VULVAL INFECTION | Total | 3 | (0.8%) | 0 | (0.0%) | 3 | (0.4%) |
|  |  | G1 | 1 | (0.3%) | 0 | (0.0%) | 1 | (0.1%) |
|  |  | G2 | 2 | (0.5%) | 0 | (0.0%) | 2 | (0.3%) |
|  | WOUND INFECTION | Total | 2 | (0.5%) | 4 | (1.1%) | 6 | (0.8%) |
|  |  | G2 | 2 | (0.5%) | 4 | (1.1%) | 6 | (0.8%) |
| INJURY, POISONING AND PROCEDURAL COMPLICATIONS | Total | Total | 23 | (6.0%) | 14 | (3.7%) | 37 | (4.9%) |
|  |  | G1 | 13 | (3.4%) | 5 | (1.3%) | 18 | (2.4%) |
|  |  | G2 | 8 | (2.1%) | 5 | (1.3%) | 13 | (1.7%) |
|  |  | G3/4/5 | 2 | (0.5%) | 5 | (1.3%) | 7 | (0.9%) |
|  | BRUISING | Total | 1 | (0.3%) | 2 | (0.5%) | 3 | (0.4%) |
|  |  | G1 | 1 | (0.3%) | 2 | (0.5%) | 3 | (0.4%) |
|  | BURN | Total | 1 | (0.3%) | 0 | (0.0%) | 1 | (0.1%) |
|  |  | G1 | 1 | (0.3%) | 0 | (0.0%) | 1 | (0.1%) |
|  | DERMATITIS RADIATION | Total | 1 | (0.3%) | 2 | (0.5%) | 3 | (0.4%) |
|  |  | G1 | 1 | (0.3%) | 0 | (0.0%) | 1 | (0.1%) |
|  |  | G3/4/5 | 0 | (0.0%) | 2 | (0.5%) | 2 | (0.3%) |
|  | FALL | Total | 3 | (0.8%) | 0 | (0.0%) | 3 | (0.4%) |
|  |  | G1 | 1 | (0.3%) | 0 | (0.0%) | 1 | (0.1%) |
|  |  | G2 | 1 | (0.3%) | 0 | (0.0%) | 1 | (0.1%) |
|  |  | G3/4/5 | 1 | (0.3%) | 0 | (0.0%) | 1 | (0.1%) |
|  | FRACTURE | Total | 15 | (3.9%) | 7 | (1.9%) | 22 | (2.9%) |
|  |  | G1 | 8 | (2.1%) | 2 | (0.5%) | 10 | (1.3%) |
|  |  | G2 | 6 | (1.6%) | 2 | (0.5%) | 8 | (1.1%) |
|  |  | G3/4/5 | 1 | (0.3%) | 3 | (0.8%) | 4 | (0.5%) |
|  | RADIATION RECALL REACTION (DERMATOLOGIC) | Total | 1 | (0.3%) | 1 | (0.3%) | 2 | (0.3%) |
|  |  | G2 | 1 | (0.3%) | 1 | (0.3%) | 2 | (0.3%) |
|  | RIGHT SCARF MASTECTOMY FLOW | Total | 0 | (0.0%) | 1 | (0.3%) | 1 | (0.1%) |
|  |  | G2 | 0 | (0.0%) | 1 | (0.3%) | 1 | (0.1%) |
|  | SPRAIN ANKLE | Total | 1 | (0.3%) | 0 | (0.0%) | 1 | (0.1%) |
|  |  | G1 | 1 | (0.3%) | 0 | (0.0%) | 1 | (0.1%) |
|  | SPRAIN KNEE | Total | 1 | (0.3%) | 0 | (0.0%) | 1 | (0.1%) |
|  |  | G1 | 1 | (0.3%) | 0 | (0.0%) | 1 | (0.1%) |
|  | WOUND COMPLICATION | Total | 0 | (0.0%) | 1 | (0.3%) | 1 | (0.1%) |
|  |  | G2 | 0 | (0.0%) | 1 | (0.3%) | 1 | (0.1%) |
|  | WOUND DEHISCENCE | Total | 0 | (0.0%) | 1 | (0.3%) | 1 | (0.1%) |
|  |  | G1 | 0 | (0.0%) | 1 | (0.3%) | 1 | (0.1%) |
| INVESTIGATIONS | Total | Total | 204 | (53.1%) | 244 | (65.2%) | 448 | (59.1%) |
|  |  | G1 | 193 | (50.3%) | 213 | (57.0%) | 406 | (53.6%) |
|  |  | G2 | 26 | (6.8%) | 84 | (22.5%) | 110 | (14.5%) |
|  |  | G3/4/5 | 13 | (3.4%) | 18 | (4.8%) | 31 | (4.1%) |
|  | ALKALINE PHOSPHATASE INCREASED | Total | 31 | (8.1%) | 27 | (7.2%) | 58 | (7.7%) |
|  |  | G1 | 31 | (8.1%) | 26 | (7.0%) | 57 | (7.5%) |
|  |  | G2 | 0 | (0.0%) | 1 | (0.3%) | 1 | (0.1%) |
|  | BLOOD BILIRUBIN DECREASED | Total | 2 | (0.5%) | 1 | (0.3%) | 3 | (0.4%) |
|  |  | G1 | 2 | (0.5%) | 1 | (0.3%) | 3 | (0.4%) |
|  | BLOOD BILIRUBIN INCREASED | Total | 3 | (0.8%) | 0 | (0.0%) | 3 | (0.4%) |
|  |  | G1 | 3 | (0.8%) | 0 | (0.0%) | 3 | (0.4%) |
|  | BLOOD PHOSPHORUS DECREASED | Total | 0 | (0.0%) | 1 | (0.3%) | 1 | (0.1%) |
|  |  | G1 | 0 | (0.0%) | 1 | (0.3%) | 1 | (0.1%) |
|  | BLOOD PHOSPHORUS INCREASED | Total | 3 | (0.8%) | 0 | (0.0%) | 3 | (0.4%) |
|  |  | G1 | 3 | (0.8%) | 0 | (0.0%) | 3 | (0.4%) |
|  | BLOOD UREA DECREASED | Total | 1 | (0.3%) | 0 | (0.0%) | 1 | (0.1%) |
|  |  | G1 | 1 | (0.3%) | 0 | (0.0%) | 1 | (0.1%) |
|  | BLOOD UREA INCREASED | Total | 2 | (0.5%) | 0 | (0.0%) | 2 | (0.3%) |
|  |  | G1 | 2 | (0.5%) | 0 | (0.0%) | 2 | (0.3%) |
|  | CHLORIDE DECREASED | Total | 0 | (0.0%) | 1 | (0.3%) | 1 | (0.1%) |
|  |  | G1 | 0 | (0.0%) | 1 | (0.3%) | 1 | (0.1%) |
|  | CHLORIDE INCREASED | Total | 2 | (0.5%) | 5 | (1.3%) | 7 | (0.9%) |
|  |  | G1 | 2 | (0.5%) | 5 | (1.3%) | 7 | (0.9%) |
|  | CHOLESTEROL HIGH | Total | 105 | (27.3%) | 131 | (35.0%) | 236 | (31.1%) |
|  |  | G1 | 97 | (25.3%) | 98 | (26.2%) | 195 | (25.7%) |
|  |  | G2 | 8 | (2.1%) | 31 | (8.3%) | 39 | (5.1%) |
|  |  | G3/4/5 | 0 | (0.0%) | 2 | (0.5%) | 2 | (0.3%) |
|  | CPK INCREASED | Total | 1 | (0.3%) | 0 | (0.0%) | 1 | (0.1%) |
|  |  | G2 | 1 | (0.3%) | 0 | (0.0%) | 1 | (0.1%) |
|  | CREATININE DECREASED | Total | 6 | (1.6%) | 4 | (1.1%) | 10 | (1.3%) |
|  |  | G1 | 6 | (1.6%) | 4 | (1.1%) | 10 | (1.3%) |
|  | CREATININE INCREASED | Total | 13 | (3.4%) | 7 | (1.9%) | 20 | (2.6%) |
|  |  | G1 | 12 | (3.1%) | 7 | (1.9%) | 19 | (2.5%) |
|  |  | G2 | 1 | (0.3%) | 0 | (0.0%) | 1 | (0.1%) |
|  | D DIMER ELEVATED | Total | 1 | (0.3%) | 0 | (0.0%) | 1 | (0.1%) |
|  |  | G2 | 1 | (0.3%) | 0 | (0.0%) | 1 | (0.1%) |
|  | FOOD INTOLERANCE | Total | 0 | (0.0%) | 1 | (0.3%) | 1 | (0.1%) |
|  |  | G1 | 0 | (0.0%) | 1 | (0.3%) | 1 | (0.1%) |
|  | HDL CHOLESTEROL DECREASE | Total | 5 | (1.3%) | 1 | (0.3%) | 6 | (0.8%) |
|  |  | G1 | 5 | (1.3%) | 1 | (0.3%) | 6 | (0.8%) |
|  | HEMOGLOBIN INCREASED | Total | 0 | (0.0%) | 4 | (1.1%) | 4 | (0.5%) |
|  |  | G1 | 0 | (0.0%) | 4 | (1.1%) | 4 | (0.5%) |
|  | HEPATIC ALAT/ASAT/GGT INCREASE | Total | 71 | (18.5%) | 111 | (29.7%) | 182 | (24.0%) |
|  |  | G1 | 57 | (14.8%) | 83 | (22.2%) | 140 | (18.5%) |
|  |  | G2 | 6 | (1.6%) | 22 | (5.9%) | 28 | (3.7%) |
|  |  | G3/4/5 | 8 | (2.1%) | 6 | (1.6%) | 14 | (1.8%) |
|  | HIGH UREA | Total | 5 | (1.3%) | 3 | (0.8%) | 8 | (1.1%) |
|  |  | G1 | 5 | (1.3%) | 3 | (0.8%) | 8 | (1.1%) |
|  | LDH INCREASE | Total | 0 | (0.0%) | 1 | (0.3%) | 1 | (0.1%) |
|  |  | G1 | 0 | (0.0%) | 1 | (0.3%) | 1 | (0.1%) |
|  | LIPASE INCREASED | Total | 1 | (0.3%) | 0 | (0.0%) | 1 | (0.1%) |
|  |  | G3/4/5 | 1 | (0.3%) | 0 | (0.0%) | 1 | (0.1%) |
|  | LYMPHOCYTE COUNT DECREASED | Total | 48 | (12.5%) | 80 | (21.4%) | 128 | (16.9%) |
|  |  | G1 | 41 | (10.7%) | 62 | (16.6%) | 103 | (13.6%) |
|  |  | G2 | 5 | (1.3%) | 15 | (4.0%) | 20 | (2.6%) |
|  |  | G3/4/5 | 2 | (0.5%) | 3 | (0.8%) | 5 | (0.7%) |
|  | LYMPHOCYTE COUNT INCREASED | Total | 1 | (0.3%) | 4 | (1.1%) | 5 | (0.7%) |
|  |  | G1 | 1 | (0.3%) | 2 | (0.5%) | 3 | (0.4%) |
|  |  | G2 | 0 | (0.0%) | 2 | (0.5%) | 2 | (0.3%) |
|  | MUCOUS DRYNESS | Total | 0 | (0.0%) | 1 | (0.3%) | 1 | (0.1%) |
|  |  | G1 | 0 | (0.0%) | 1 | (0.3%) | 1 | (0.1%) |
|  | NEUTROPHIL COUNT DECREASED | Total | 14 | (3.6%) | 62 | (16.6%) | 76 | (10.0%) |
|  |  | G1 | 11 | (2.9%) | 35 | (9.4%) | 46 | (6.1%) |
|  |  | G2 | 2 | (0.5%) | 24 | (6.4%) | 26 | (3.4%) |
|  |  | G3/4/5 | 1 | (0.3%) | 3 | (0.8%) | 4 | (0.5%) |
|  | PLATELET COUNT DECREASED | Total | 8 | (2.1%) | 33 | (8.8%) | 41 | (5.4%) |
|  |  | G1 | 8 | (2.1%) | 32 | (8.6%) | 40 | (5.3%) |
|  |  | G2 | 0 | (0.0%) | 1 | (0.3%) | 1 | (0.1%) |
|  | PLATELET COUNT INCREASED | Total | 2 | (0.5%) | 0 | (0.0%) | 2 | (0.3%) |
|  |  | G1 | 2 | (0.5%) | 0 | (0.0%) | 2 | (0.3%) |
|  | SERUM AMYLASE INCREASED | Total | 1 | (0.3%) | 0 | (0.0%) | 1 | (0.1%) |
|  |  | G3/4/5 | 1 | (0.3%) | 0 | (0.0%) | 1 | (0.1%) |
|  | WEIGHT GAIN | Total | 3 | (0.8%) | 2 | (0.5%) | 5 | (0.7%) |
|  |  | G1 | 0 | (0.0%) | 2 | (0.5%) | 2 | (0.3%) |
|  |  | G2 | 2 | (0.5%) | 0 | (0.0%) | 2 | (0.3%) |
|  |  | G3/4/5 | 1 | (0.3%) | 0 | (0.0%) | 1 | (0.1%) |
|  | WEIGHT LOSS | Total | 4 | (1.0%) | 32 | (8.6%) | 36 | (4.7%) |
|  |  | G1 | 3 | (0.8%) | 20 | (5.3%) | 23 | (3.0%) |
|  |  | G2 | 1 | (0.3%) | 8 | (2.1%) | 9 | (1.2%) |
|  |  | G3/4/5 | 0 | (0.0%) | 4 | (1.1%) | 4 | (0.5%) |
|  | WHITE BLOOD CELL DECREASED | Total | 14 | (3.6%) | 32 | (8.6%) | 46 | (6.1%) |
|  |  | G1 | 12 | (3.1%) | 25 | (6.7%) | 37 | (4.9%) |
|  |  | G2 | 1 | (0.3%) | 6 | (1.6%) | 7 | (0.9%) |
|  |  | G3/4/5 | 1 | (0.3%) | 1 | (0.3%) | 2 | (0.3%) |
| METABOLISM AND NUTRITION DISORDERS | Total | Total | 144 | (37.5%) | 190 | (50.8%) | 334 | (44.1%) |
|  |  | . | 1 | (0.3%) | 1 | (0.3%) | 2 | (0.3%) |
|  |  | G1 | 137 | (35.7%) | 164 | (43.9%) | 301 | (39.7%) |
|  |  | G2 | 12 | (3.1%) | 44 | (11.8%) | 56 | (7.4%) |
|  |  | G3/4/5 | 1 | (0.3%) | 15 | (4.0%) | 16 | (2.1%) |
|  | ANOREXIA | Total | 17 | (4.4%) | 50 | (13.4%) | 67 | (8.8%) |
|  |  | G1 | 14 | (3.6%) | 41 | (11.0%) | 55 | (7.3%) |
|  |  | G2 | 3 | (0.8%) | 8 | (2.1%) | 11 | (1.5%) |
|  |  | G3/4/5 | 0 | (0.0%) | 1 | (0.3%) | 1 | (0.1%) |
|  | ATHEROMATOUS OVERLOAD | Total | 1 | (0.3%) | 0 | (0.0%) | 1 | (0.1%) |
|  |  | . | 1 | (0.3%) | 0 | (0.0%) | 1 | (0.1%) |
|  | HYPERCALCEMIA | Total | 29 | (7.6%) | 8 | (2.1%) | 37 | (4.9%) |
|  |  | G1 | 27 | (7.0%) | 8 | (2.1%) | 35 | (4.6%) |
|  |  | G2 | 1 | (0.3%) | 0 | (0.0%) | 1 | (0.1%) |
|  |  | G3/4/5 | 1 | (0.3%) | 0 | (0.0%) | 1 | (0.1%) |
|  | HYPERGLYCEMIA | Total | 41 | (10.7%) | 73 | (19.5%) | 114 | (15.0%) |
|  |  | G1 | 39 | (10.2%) | 55 | (14.7%) | 94 | (12.4%) |
|  |  | G2 | 2 | (0.5%) | 12 | (3.2%) | 14 | (1.8%) |
|  |  | G3/4/5 | 0 | (0.0%) | 6 | (1.6%) | 6 | (0.8%) |
|  | HYPERKALEMIA | Total | 22 | (5.7%) | 14 | (3.7%) | 36 | (4.7%) |
|  |  | G1 | 22 | (5.7%) | 14 | (3.7%) | 36 | (4.7%) |
|  | HYPERNATREMIA | Total | 6 | (1.6%) | 6 | (1.6%) | 12 | (1.6%) |
|  |  | . | 0 | (0.0%) | 1 | (0.3%) | 1 | (0.1%) |
|  |  | G1 | 6 | (1.6%) | 5 | (1.3%) | 11 | (1.5%) |
|  | HYPERTRIGLYCERIDEMIA | Total | 55 | (14.3%) | 108 | (28.9%) | 163 | (21.5%) |
|  |  | G1 | 51 | (13.3%) | 77 | (20.6%) | 128 | (16.9%) |
|  |  | G2 | 4 | (1.0%) | 26 | (7.0%) | 30 | (4.0%) |
|  |  | G3/4/5 | 0 | (0.0%) | 5 | (1.3%) | 5 | (0.7%) |
|  | HYPERURICEMIA | Total | 7 | (1.8%) | 5 | (1.3%) | 12 | (1.6%) |
|  |  | G1 | 7 | (1.8%) | 4 | (1.1%) | 11 | (1.5%) |
|  |  | G3/4/5 | 0 | (0.0%) | 1 | (0.3%) | 1 | (0.1%) |
|  | HYPOALBUMINEMIA | Total | 0 | (0.0%) | 1 | (0.3%) | 1 | (0.1%) |
|  |  | G1 | 0 | (0.0%) | 1 | (0.3%) | 1 | (0.1%) |
|  | HYPOCALCEMIA | Total | 3 | (0.8%) | 3 | (0.8%) | 6 | (0.8%) |
|  |  | G1 | 3 | (0.8%) | 2 | (0.5%) | 5 | (0.7%) |
|  |  | G2 | 0 | (0.0%) | 1 | (0.3%) | 1 | (0.1%) |
|  | HYPOGLYCEMIA | Total | 2 | (0.5%) | 0 | (0.0%) | 2 | (0.3%) |
|  |  | G1 | 2 | (0.5%) | 0 | (0.0%) | 2 | (0.3%) |
|  | HYPOKALEMIA | Total | 6 | (1.6%) | 10 | (2.7%) | 16 | (2.1%) |
|  |  | G1 | 6 | (1.6%) | 6 | (1.6%) | 12 | (1.6%) |
|  |  | G2 | 0 | (0.0%) | 2 | (0.5%) | 2 | (0.3%) |
|  |  | G3/4/5 | 0 | (0.0%) | 2 | (0.5%) | 2 | (0.3%) |
|  | HYPOMAGNESEMIA | Total | 1 | (0.3%) | 2 | (0.5%) | 3 | (0.4%) |
|  |  | G1 | 1 | (0.3%) | 1 | (0.3%) | 2 | (0.3%) |
|  |  | G2 | 0 | (0.0%) | 1 | (0.3%) | 1 | (0.1%) |
|  | HYPONATREMIA | Total | 1 | (0.3%) | 1 | (0.3%) | 2 | (0.3%) |
|  |  | G1 | 1 | (0.3%) | 1 | (0.3%) | 2 | (0.3%) |
|  | HYPOPHOSPHATEMIA | Total | 4 | (1.0%) | 3 | (0.8%) | 7 | (0.9%) |
|  |  | G1 | 3 | (0.8%) | 3 | (0.8%) | 6 | (0.8%) |
|  |  | G2 | 1 | (0.3%) | 0 | (0.0%) | 1 | (0.1%) |
|  | IRON DEFICIENCY | Total | 0 | (0.0%) | 1 | (0.3%) | 1 | (0.1%) |
|  |  | G1 | 0 | (0.0%) | 1 | (0.3%) | 1 | (0.1%) |
|  | PHOSPHORE INCREASED | Total | 1 | (0.3%) | 1 | (0.3%) | 2 | (0.3%) |
|  |  | G1 | 1 | (0.3%) | 1 | (0.3%) | 2 | (0.3%) |
|  | RAISED BLOOD GLOBULIN | Total | 1 | (0.3%) | 0 | (0.0%) | 1 | (0.1%) |
|  |  | G1 | 1 | (0.3%) | 0 | (0.0%) | 1 | (0.1%) |
|  | VITAMIN DEFICIENCY | Total | 3 | (0.8%) | 1 | (0.3%) | 4 | (0.5%) |
|  |  | G1 | 1 | (0.3%) | 0 | (0.0%) | 1 | (0.1%) |
|  |  | G2 | 2 | (0.5%) | 1 | (0.3%) | 3 | (0.4%) |
| MUSCULOSKELETAL AND CONNECTIVE TISSUE DISORDERS | Total | Total | 167 | (43.5%) | 123 | (32.9%) | 290 | (38.3%) |
|  |  | . | 3 | (0.8%) | 4 | (1.1%) | 7 | (0.9%) |
|  |  | G1 | 133 | (34.6%) | 98 | (26.2%) | 231 | (30.5%) |
|  |  | G2 | 40 | (10.4%) | 32 | (8.6%) | 72 | (9.5%) |
|  |  | G3/4/5 | 3 | (0.8%) | 3 | (0.8%) | 6 | (0.8%) |
|  | ARTHRALGIA | Total | 118 | (30.7%) | 70 | (18.7%) | 188 | (24.8%) |
|  |  | . | 2 | (0.5%) | 0 | (0.0%) | 2 | (0.3%) |
|  |  | G1 | 91 | (23.7%) | 54 | (14.4%) | 145 | (19.1%) |
|  |  | G2 | 24 | (6.3%) | 14 | (3.7%) | 38 | (5.0%) |
|  |  | G3/4/5 | 1 | (0.3%) | 2 | (0.5%) | 3 | (0.4%) |
|  | ARTHRITIS | Total | 6 | (1.6%) | 3 | (0.8%) | 9 | (1.2%) |
|  |  | . | 1 | (0.3%) | 0 | (0.0%) | 1 | (0.1%) |
|  |  | G1 | 4 | (1.0%) | 3 | (0.8%) | 7 | (0.9%) |
|  |  | G3/4/5 | 1 | (0.3%) | 0 | (0.0%) | 1 | (0.1%) |
|  | BACK PAIN | Total | 19 | (4.9%) | 21 | (5.6%) | 40 | (5.3%) |
|  |  | G1 | 14 | (3.6%) | 16 | (4.3%) | 30 | (4.0%) |
|  |  | G2 | 5 | (1.3%) | 4 | (1.1%) | 9 | (1.2%) |
|  |  | G3/4/5 | 0 | (0.0%) | 1 | (0.3%) | 1 | (0.1%) |
|  | BONE PAIN | Total | 8 | (2.1%) | 12 | (3.2%) | 20 | (2.6%) |
|  |  | G1 | 4 | (1.0%) | 9 | (2.4%) | 13 | (1.7%) |
|  |  | G2 | 3 | (0.8%) | 3 | (0.8%) | 6 | (0.8%) |
|  |  | G3/4/5 | 1 | (0.3%) | 0 | (0.0%) | 1 | (0.1%) |
|  | CHEST WALL PAIN | Total | 7 | (1.8%) | 5 | (1.3%) | 12 | (1.6%) |
|  |  | G1 | 4 | (1.0%) | 4 | (1.1%) | 8 | (1.1%) |
|  |  | G2 | 3 | (0.8%) | 1 | (0.3%) | 4 | (0.5%) |
|  | ENTHESITIS | Total | 0 | (0.0%) | 1 | (0.3%) | 1 | (0.1%) |
|  |  | G1 | 0 | (0.0%) | 1 | (0.3%) | 1 | (0.1%) |
|  | EXOSTOSIS | Total | 1 | (0.3%) | 0 | (0.0%) | 1 | (0.1%) |
|  |  | G1 | 1 | (0.3%) | 0 | (0.0%) | 1 | (0.1%) |
|  | FIBROSIS DEEP CONNECTIVE TISSUE | Total | 1 | (0.3%) | 0 | (0.0%) | 1 | (0.1%) |
|  |  | G2 | 1 | (0.3%) | 0 | (0.0%) | 1 | (0.1%) |
|  | FLANK PAIN | Total | 1 | (0.3%) | 0 | (0.0%) | 1 | (0.1%) |
|  |  | G1 | 1 | (0.3%) | 0 | (0.0%) | 1 | (0.1%) |
|  | JOINT RANGE OF MOTION DECREASED | Total | 1 | (0.3%) | 0 | (0.0%) | 1 | (0.1%) |
|  |  | G1 | 1 | (0.3%) | 0 | (0.0%) | 1 | (0.1%) |
|  | LOMBO SCIATALGIA | Total | 0 | (0.0%) | 1 | (0.3%) | 1 | (0.1%) |
|  |  | . | 0 | (0.0%) | 1 | (0.3%) | 1 | (0.1%) |
|  | MUSCLE WEAKNESS UPPER LIMB | Total | 0 | (0.0%) | 1 | (0.3%) | 1 | (0.1%) |
|  |  | G1 | 0 | (0.0%) | 1 | (0.3%) | 1 | (0.1%) |
|  | MUSCULOSKELETAL DEFORMITY | Total | 1 | (0.3%) | 0 | (0.0%) | 1 | (0.1%) |
|  |  | G1 | 1 | (0.3%) | 0 | (0.0%) | 1 | (0.1%) |
|  | MYALGIA | Total | 28 | (7.3%) | 21 | (5.6%) | 49 | (6.5%) |
|  |  | . | 0 | (0.0%) | 2 | (0.5%) | 2 | (0.3%) |
|  |  | G1 | 25 | (6.5%) | 15 | (4.0%) | 40 | (5.3%) |
|  |  | G2 | 3 | (0.8%) | 4 | (1.1%) | 7 | (0.9%) |
|  | MYOSITIS | Total | 1 | (0.3%) | 1 | (0.3%) | 2 | (0.3%) |
|  |  | G1 | 1 | (0.3%) | 1 | (0.3%) | 2 | (0.3%) |
|  | NECK PAIN | Total | 4 | (1.0%) | 0 | (0.0%) | 4 | (0.5%) |
|  |  | G1 | 2 | (0.5%) | 0 | (0.0%) | 2 | (0.3%) |
|  |  | G2 | 2 | (0.5%) | 0 | (0.0%) | 2 | (0.3%) |
|  | NUMBNESS (RIGHT FOOT) | Total | 0 | (0.0%) | 1 | (0.3%) | 1 | (0.1%) |
|  |  | G1 | 0 | (0.0%) | 1 | (0.3%) | 1 | (0.1%) |
|  | OSTEONECROSIS OF JAW | Total | 0 | (0.0%) | 2 | (0.5%) | 2 | (0.3%) |
|  |  | G2 | 0 | (0.0%) | 2 | (0.5%) | 2 | (0.3%) |
|  | OSTEOPOROSIS | Total | 5 | (1.3%) | 3 | (0.8%) | 8 | (1.1%) |
|  |  | G1 | 5 | (1.3%) | 2 | (0.5%) | 7 | (0.9%) |
|  |  | G2 | 0 | (0.0%) | 1 | (0.3%) | 1 | (0.1%) |
|  | PAIN IN EXTREMITY | Total | 7 | (1.8%) | 9 | (2.4%) | 16 | (2.1%) |
|  |  | G1 | 7 | (1.8%) | 7 | (1.9%) | 14 | (1.8%) |
|  |  | G2 | 0 | (0.0%) | 2 | (0.5%) | 2 | (0.3%) |
|  | R MASTECTOMY TENDERNESS TO SCAR NERVE | Total | 1 | (0.3%) | 0 | (0.0%) | 1 | (0.1%) |
|  |  | G1 | 1 | (0.3%) | 0 | (0.0%) | 1 | (0.1%) |
|  | STIFFNESS | Total | 2 | (0.5%) | 4 | (1.1%) | 6 | (0.8%) |
|  |  | . | 0 | (0.0%) | 1 | (0.3%) | 1 | (0.1%) |
|  |  | G1 | 2 | (0.5%) | 3 | (0.8%) | 5 | (0.7%) |
|  | TENDINITIS | Total | 6 | (1.6%) | 4 | (1.1%) | 10 | (1.3%) |
|  |  | G1 | 2 | (0.5%) | 3 | (0.8%) | 5 | (0.7%) |
|  |  | G2 | 4 | (1.0%) | 1 | (0.3%) | 5 | (0.7%) |
| NEOPLASMS BENIGN, MALIGNANT AND UNSPECIFIED (INCL CYSTS AND POLYPS) | Total | Total | 10 | (2.6%) | 8 | (2.1%) | 18 | (2.4%) |
|  |  | G1 | 1 | (0.3%) | 2 | (0.5%) | 3 | (0.4%) |
|  |  | G2 | 3 | (0.8%) | 3 | (0.8%) | 6 | (0.8%) |
|  |  | G3/4/5 | 6 | (1.6%) | 3 | (0.8%) | 9 | (1.2%) |
|  | ADENOMA THYROID | Total | 0 | (0.0%) | 2 | (0.5%) | 2 | (0.3%) |
|  |  | G1 | 0 | (0.0%) | 2 | (0.5%) | 2 | (0.3%) |
|  | BASAL CELL CARCINOMA (RIGHT TEMPLE) | Total | 1 | (0.3%) | 0 | (0.0%) | 1 | (0.1%) |
|  |  | G3/4/5 | 1 | (0.3%) | 0 | (0.0%) | 1 | (0.1%) |
|  | BENIN LEFT OVARIAN CYST | Total | 1 | (0.3%) | 0 | (0.0%) | 1 | (0.1%) |
|  |  | G2 | 1 | (0.3%) | 0 | (0.0%) | 1 | (0.1%) |
|  | BREAST CYST | Total | 2 | (0.5%) | 0 | (0.0%) | 2 | (0.3%) |
|  |  | G2 | 2 | (0.5%) | 0 | (0.0%) | 2 | (0.3%) |
|  | CANCER OF ENDOMETRIUM | Total | 1 | (0.3%) | 0 | (0.0%) | 1 | (0.1%) |
|  |  | G3/4/5 | 1 | (0.3%) | 0 | (0.0%) | 1 | (0.1%) |
|  | COLON | Total | 1 | (0.3%) | 0 | (0.0%) | 1 | (0.1%) |
|  |  | G3/4/5 | 1 | (0.3%) | 0 | (0.0%) | 1 | (0.1%) |
|  | CONTRALATERAL BREAST CANCER | Total | 1 | (0.3%) | 0 | (0.0%) | 1 | (0.1%) |
|  |  | G3/4/5 | 1 | (0.3%) | 0 | (0.0%) | 1 | (0.1%) |
|  | CYST | Total | 1 | (0.3%) | 0 | (0.0%) | 1 | (0.1%) |
|  |  | G1 | 1 | (0.3%) | 0 | (0.0%) | 1 | (0.1%) |
|  | FOLLICULAR THYROID CANCER | Total | 0 | (0.0%) | 1 | (0.3%) | 1 | (0.1%) |
|  |  | G2 | 0 | (0.0%) | 1 | (0.3%) | 1 | (0.1%) |
|  | INTESTINAL ADENOCARCINOMA | Total | 1 | (0.3%) | 0 | (0.0%) | 1 | (0.1%) |
|  |  | G3/4/5 | 1 | (0.3%) | 0 | (0.0%) | 1 | (0.1%) |
|  | MENINGIOMA | Total | 0 | (0.0%) | 1 | (0.3%) | 1 | (0.1%) |
|  |  | G2 | 0 | (0.0%) | 1 | (0.3%) | 1 | (0.1%) |
|  | PERITONEAL CARCINOMA | Total | 1 | (0.3%) | 0 | (0.0%) | 1 | (0.1%) |
|  |  | G3/4/5 | 1 | (0.3%) | 0 | (0.0%) | 1 | (0.1%) |
|  | RENAL ONCOCYTOMA | Total | 0 | (0.0%) | 1 | (0.3%) | 1 | (0.1%) |
|  |  | G3/4/5 | 0 | (0.0%) | 1 | (0.3%) | 1 | (0.1%) |
|  | SMALL CELL LUNG CANCER | Total | 0 | (0.0%) | 1 | (0.3%) | 1 | (0.1%) |
|  |  | G3/4/5 | 0 | (0.0%) | 1 | (0.3%) | 1 | (0.1%) |
|  | TRICHOBLASTIC CARCINOMA | Total | 1 | (0.3%) | 0 | (0.0%) | 1 | (0.1%) |
|  |  | G3/4/5 | 1 | (0.3%) | 0 | (0.0%) | 1 | (0.1%) |
|  | TUMOUR PROGRESSION | Total | 0 | (0.0%) | 1 | (0.3%) | 1 | (0.1%) |
|  |  | G3/4/5 | 0 | (0.0%) | 1 | (0.3%) | 1 | (0.1%) |
|  | UROTHELIAL CARCINOMA | Total | 0 | (0.0%) | 1 | (0.3%) | 1 | (0.1%) |
|  |  | G2 | 0 | (0.0%) | 1 | (0.3%) | 1 | (0.1%) |
| NERVOUS SYSTEM DISORDERS | Total | Total | 95 | (24.7%) | 154 | (41.2%) | 249 | (32.8%) |
|  |  | . | 0 | (0.0%) | 1 | (0.3%) | 1 | (0.1%) |
|  |  | G1 | 69 | (18.0%) | 132 | (35.3%) | 201 | (26.5%) |
|  |  | G2 | 26 | (6.8%) | 32 | (8.6%) | 58 | (7.7%) |
|  |  | G3/4/5 | 3 | (0.8%) | 1 | (0.3%) | 4 | (0.5%) |
|  | ACOUSTIC NERVE DISORDER NOS | Total | 0 | (0.0%) | 1 | (0.3%) | 1 | (0.1%) |
|  |  | G1 | 0 | (0.0%) | 1 | (0.3%) | 1 | (0.1%) |
|  | AMNESIA | Total | 2 | (0.5%) | 0 | (0.0%) | 2 | (0.3%) |
|  |  | G2 | 2 | (0.5%) | 0 | (0.0%) | 2 | (0.3%) |
|  | APHONIA | Total | 2 | (0.5%) | 1 | (0.3%) | 3 | (0.4%) |
|  |  | G1 | 0 | (0.0%) | 1 | (0.3%) | 1 | (0.1%) |
|  |  | G2 | 2 | (0.5%) | 0 | (0.0%) | 2 | (0.3%) |
|  | BRACHIAL PLEXOPATHY | Total | 0 | (0.0%) | 1 | (0.3%) | 1 | (0.1%) |
|  |  | G1 | 0 | (0.0%) | 1 | (0.3%) | 1 | (0.1%) |
|  | BURNING SENSATION | Total | 0 | (0.0%) | 1 | (0.3%) | 1 | (0.1%) |
|  |  | G1 | 0 | (0.0%) | 1 | (0.3%) | 1 | (0.1%) |
|  | COGNITIVE DISTURBANCE | Total | 1 | (0.3%) | 1 | (0.3%) | 2 | (0.3%) |
|  |  | G1 | 1 | (0.3%) | 1 | (0.3%) | 2 | (0.3%) |
|  | CONCENTRATION IMPAIRMENT | Total | 1 | (0.3%) | 0 | (0.0%) | 1 | (0.1%) |
|  |  | G1 | 1 | (0.3%) | 0 | (0.0%) | 1 | (0.1%) |
|  | DIZZINESS | Total | 9 | (2.3%) | 9 | (2.4%) | 18 | (2.4%) |
|  |  | G1 | 8 | (2.1%) | 9 | (2.4%) | 17 | (2.2%) |
|  |  | G2 | 1 | (0.3%) | 0 | (0.0%) | 1 | (0.1%) |
|  | DYSESTHESIA | Total | 5 | (1.3%) | 3 | (0.8%) | 8 | (1.1%) |
|  |  | G1 | 4 | (1.0%) | 2 | (0.5%) | 6 | (0.8%) |
|  |  | G2 | 1 | (0.3%) | 0 | (0.0%) | 1 | (0.1%) |
|  |  | G3/4/5 | 0 | (0.0%) | 1 | (0.3%) | 1 | (0.1%) |
|  | DYSGEUSIA | Total | 20 | (5.2%) | 62 | (16.6%) | 82 | (10.8%) |
|  |  | G1 | 17 | (4.4%) | 54 | (14.4%) | 71 | (9.4%) |
|  |  | G2 | 3 | (0.8%) | 8 | (2.1%) | 11 | (1.5%) |
|  | HEADACHE | Total | 44 | (11.5%) | 74 | (19.8%) | 118 | (15.6%) |
|  |  | G1 | 31 | (8.1%) | 57 | (15.2%) | 88 | (11.6%) |
|  |  | G2 | 12 | (3.1%) | 17 | (4.5%) | 29 | (3.8%) |
|  |  | G3/4/5 | 1 | (0.3%) | 0 | (0.0%) | 1 | (0.1%) |
|  | HYPEROESTHESIA | Total | 0 | (0.0%) | 1 | (0.3%) | 1 | (0.1%) |
|  |  | G1 | 0 | (0.0%) | 1 | (0.3%) | 1 | (0.1%) |
|  | LETHARGY | Total | 1 | (0.3%) | 0 | (0.0%) | 1 | (0.1%) |
|  |  | G1 | 1 | (0.3%) | 0 | (0.0%) | 1 | (0.1%) |
|  | MEMORY IMPAIRMENT | Total | 4 | (1.0%) | 2 | (0.5%) | 6 | (0.8%) |
|  |  | G1 | 3 | (0.8%) | 2 | (0.5%) | 5 | (0.7%) |
|  |  | G2 | 1 | (0.3%) | 0 | (0.0%) | 1 | (0.1%) |
|  | NEURALGIA | Total | 3 | (0.8%) | 5 | (1.3%) | 8 | (1.1%) |
|  |  | G1 | 1 | (0.3%) | 3 | (0.8%) | 4 | (0.5%) |
|  |  | G2 | 2 | (0.5%) | 2 | (0.5%) | 4 | (0.5%) |
|  | OCULOMOTOR NERVE DISORDER | Total | 1 | (0.3%) | 0 | (0.0%) | 1 | (0.1%) |
|  |  | G1 | 1 | (0.3%) | 0 | (0.0%) | 1 | (0.1%) |
|  | PARESTHESIA | Total | 9 | (2.3%) | 18 | (4.8%) | 27 | (3.6%) |
|  |  | G1 | 8 | (2.1%) | 16 | (4.3%) | 24 | (3.2%) |
|  |  | G2 | 1 | (0.3%) | 2 | (0.5%) | 3 | (0.4%) |
|  | PERIPHERAL MOTOR NEUROPATHY | Total | 2 | (0.5%) | 3 | (0.8%) | 5 | (0.7%) |
|  |  | G1 | 1 | (0.3%) | 2 | (0.5%) | 3 | (0.4%) |
|  |  | G2 | 1 | (0.3%) | 1 | (0.3%) | 2 | (0.3%) |
|  | PERIPHERAL SENSORY NEUROPATHY | Total | 5 | (1.3%) | 10 | (2.7%) | 15 | (2.0%) |
|  |  | . | 0 | (0.0%) | 1 | (0.3%) | 1 | (0.1%) |
|  |  | G1 | 4 | (1.0%) | 5 | (1.3%) | 9 | (1.2%) |
|  |  | G2 | 1 | (0.3%) | 4 | (1.1%) | 5 | (0.7%) |
|  | RIGHT L5 SCIATICA | Total | 1 | (0.3%) | 0 | (0.0%) | 1 | (0.1%) |
|  |  | G3/4/5 | 1 | (0.3%) | 0 | (0.0%) | 1 | (0.1%) |
|  | SINUS PAIN | Total | 1 | (0.3%) | 0 | (0.0%) | 1 | (0.1%) |
|  |  | G2 | 1 | (0.3%) | 0 | (0.0%) | 1 | (0.1%) |
|  | SPASTICITY | Total | 1 | (0.3%) | 0 | (0.0%) | 1 | (0.1%) |
|  |  | G1 | 1 | (0.3%) | 0 | (0.0%) | 1 | (0.1%) |
|  | SYNCOPE | Total | 1 | (0.3%) | 0 | (0.0%) | 1 | (0.1%) |
|  |  | G3/4/5 | 1 | (0.3%) | 0 | (0.0%) | 1 | (0.1%) |
|  | TREMOR | Total | 0 | (0.0%) | 2 | (0.5%) | 2 | (0.3%) |
|  |  | G1 | 0 | (0.0%) | 2 | (0.5%) | 2 | (0.3%) |
| PSYCHIATRIC DISORDERS | Total | Total | 61 | (15.9%) | 52 | (13.9%) | 113 | (14.9%) |
|  |  | . | 0 | (0.0%) | 1 | (0.3%) | 1 | (0.1%) |
|  |  | G1 | 48 | (12.5%) | 33 | (8.8%) | 81 | (10.7%) |
|  |  | G2 | 11 | (2.9%) | 17 | (4.5%) | 28 | (3.7%) |
|  |  | G3/4/5 | 2 | (0.5%) | 1 | (0.3%) | 3 | (0.4%) |
|  | AGITATION | Total | 0 | (0.0%) | 1 | (0.3%) | 1 | (0.1%) |
|  |  | G2 | 0 | (0.0%) | 1 | (0.3%) | 1 | (0.1%) |
|  | ANXIETY | Total | 10 | (2.6%) | 7 | (1.9%) | 17 | (2.2%) |
|  |  | G1 | 9 | (2.3%) | 2 | (0.5%) | 11 | (1.5%) |
|  |  | G2 | 1 | (0.3%) | 5 | (1.3%) | 6 | (0.8%) |
|  | DEPRESSION | Total | 13 | (3.4%) | 14 | (3.7%) | 27 | (3.6%) |
|  |  | . | 0 | (0.0%) | 1 | (0.3%) | 1 | (0.1%) |
|  |  | G1 | 10 | (2.6%) | 9 | (2.4%) | 19 | (2.5%) |
|  |  | G2 | 2 | (0.5%) | 4 | (1.1%) | 6 | (0.8%) |
|  |  | G3/4/5 | 1 | (0.3%) | 0 | (0.0%) | 1 | (0.1%) |
|  | INSOMNIA | Total | 38 | (9.9%) | 26 | (7.0%) | 64 | (8.4%) |
|  |  | G1 | 30 | (7.8%) | 19 | (5.1%) | 49 | (6.5%) |
|  |  | G2 | 8 | (2.1%) | 7 | (1.9%) | 15 | (2.0%) |
|  | LIBIDO DECREASED | Total | 0 | (0.0%) | 1 | (0.3%) | 1 | (0.1%) |
|  |  | G1 | 0 | (0.0%) | 1 | (0.3%) | 1 | (0.1%) |
|  | LIBIDO INCREASED | Total | 1 | (0.3%) | 0 | (0.0%) | 1 | (0.1%) |
|  |  | G1 | 1 | (0.3%) | 0 | (0.0%) | 1 | (0.1%) |
|  | MOOD DISORDER | Total | 0 | (0.0%) | 2 | (0.5%) | 2 | (0.3%) |
|  |  | G1 | 0 | (0.0%) | 2 | (0.5%) | 2 | (0.3%) |
|  | SUICIDE ATTEMPT | Total | 1 | (0.3%) | 1 | (0.3%) | 2 | (0.3%) |
|  |  | G3/4/5 | 1 | (0.3%) | 1 | (0.3%) | 2 | (0.3%) |
| RENAL AND URINARY DISORDERS | Total | Total | 23 | (6.0%) | 25 | (6.7%) | 48 | (6.3%) |
|  |  | G1 | 21 | (5.5%) | 18 | (4.8%) | 39 | (5.1%) |
|  |  | G2 | 3 | (0.8%) | 7 | (1.9%) | 10 | (1.3%) |
|  | CHRONIC KIDNEY DISEASE | Total | 0 | (0.0%) | 1 | (0.3%) | 1 | (0.1%) |
|  |  | G2 | 0 | (0.0%) | 1 | (0.3%) | 1 | (0.1%) |
|  | CYSTITIS NONINFECTIVE | Total | 6 | (1.6%) | 10 | (2.7%) | 16 | (2.1%) |
|  |  | G1 | 6 | (1.6%) | 7 | (1.9%) | 13 | (1.7%) |
|  |  | G2 | 0 | (0.0%) | 3 | (0.8%) | 3 | (0.4%) |
|  | HEMATURIA | Total | 2 | (0.5%) | 1 | (0.3%) | 3 | (0.4%) |
|  |  | G1 | 1 | (0.3%) | 0 | (0.0%) | 1 | (0.1%) |
|  |  | G2 | 1 | (0.3%) | 1 | (0.3%) | 2 | (0.3%) |
|  | PROTEINURIA | Total | 1 | (0.3%) | 0 | (0.0%) | 1 | (0.1%) |
|  |  | G1 | 1 | (0.3%) | 0 | (0.0%) | 1 | (0.1%) |
|  | RENAL CALCULI | Total | 1 | (0.3%) | 0 | (0.0%) | 1 | (0.1%) |
|  |  | G2 | 1 | (0.3%) | 0 | (0.0%) | 1 | (0.1%) |
|  | RENAL INSUFFICIENCY | Total | 1 | (0.3%) | 0 | (0.0%) | 1 | (0.1%) |
|  |  | G2 | 1 | (0.3%) | 0 | (0.0%) | 1 | (0.1%) |
|  | UREA DECREASED | Total | 1 | (0.3%) | 1 | (0.3%) | 2 | (0.3%) |
|  |  | G1 | 1 | (0.3%) | 1 | (0.3%) | 2 | (0.3%) |
|  | UREA INCREASED | Total | 10 | (2.6%) | 4 | (1.1%) | 14 | (1.8%) |
|  |  | G1 | 10 | (2.6%) | 4 | (1.1%) | 14 | (1.8%) |
|  | URINARY FREQUENCY | Total | 1 | (0.3%) | 1 | (0.3%) | 2 | (0.3%) |
|  |  | G1 | 0 | (0.0%) | 1 | (0.3%) | 1 | (0.1%) |
|  |  | G2 | 1 | (0.3%) | 0 | (0.0%) | 1 | (0.1%) |
|  | URINARY INCONTINENCE | Total | 2 | (0.5%) | 2 | (0.5%) | 4 | (0.5%) |
|  |  | G1 | 2 | (0.5%) | 1 | (0.3%) | 3 | (0.4%) |
|  |  | G2 | 0 | (0.0%) | 1 | (0.3%) | 1 | (0.1%) |
|  | URINARY RETENTION | Total | 0 | (0.0%) | 2 | (0.5%) | 2 | (0.3%) |
|  |  | G1 | 0 | (0.0%) | 2 | (0.5%) | 2 | (0.3%) |
|  | URINARY TRACT PAIN | Total | 2 | (0.5%) | 2 | (0.5%) | 4 | (0.5%) |
|  |  | G1 | 1 | (0.3%) | 1 | (0.3%) | 2 | (0.3%) |
|  |  | G2 | 1 | (0.3%) | 1 | (0.3%) | 2 | (0.3%) |
|  | URINARY URGENCY | Total | 1 | (0.3%) | 1 | (0.3%) | 2 | (0.3%) |
|  |  | G1 | 1 | (0.3%) | 1 | (0.3%) | 2 | (0.3%) |
|  | URINE DISCOLORATION | Total | 0 | (0.0%) | 1 | (0.3%) | 1 | (0.1%) |
|  |  | G1 | 0 | (0.0%) | 1 | (0.3%) | 1 | (0.1%) |
| REPRODUCTIVE SYSTEM AND BREAST DISORDERS | Total | Total | 24 | (6.3%) | 23 | (6.1%) | 47 | (6.2%) |
|  |  | . | 0 | (0.0%) | 1 | (0.3%) | 1 | (0.1%) |
|  |  | G1 | 21 | (5.5%) | 17 | (4.5%) | 38 | (5.0%) |
|  |  | G2 | 3 | (0.8%) | 5 | (1.3%) | 8 | (1.1%) |
|  | BREAST PAIN | Total | 9 | (2.3%) | 5 | (1.3%) | 14 | (1.8%) |
|  |  | . | 0 | (0.0%) | 1 | (0.3%) | 1 | (0.1%) |
|  |  | G1 | 8 | (2.1%) | 4 | (1.1%) | 12 | (1.6%) |
|  |  | G2 | 1 | (0.3%) | 0 | (0.0%) | 1 | (0.1%) |
|  | IRREGULAR MENSTRUATION | Total | 2 | (0.5%) | 0 | (0.0%) | 2 | (0.3%) |
|  |  | G1 | 2 | (0.5%) | 0 | (0.0%) | 2 | (0.3%) |
|  | NIPPLE DEFORMITY | Total | 1 | (0.3%) | 0 | (0.0%) | 1 | (0.1%) |
|  |  | G1 | 1 | (0.3%) | 0 | (0.0%) | 1 | (0.1%) |
|  | PELVIC PAIN | Total | 2 | (0.5%) | 1 | (0.3%) | 3 | (0.4%) |
|  |  | G1 | 2 | (0.5%) | 1 | (0.3%) | 3 | (0.4%) |
|  | PREMATURE MENOPAUSE | Total | 1 | (0.3%) | 0 | (0.0%) | 1 | (0.1%) |
|  |  | G1 | 1 | (0.3%) | 0 | (0.0%) | 1 | (0.1%) |
|  | VAGINAL DISCHARGE | Total | 1 | (0.3%) | 1 | (0.3%) | 2 | (0.3%) |
|  |  | G1 | 1 | (0.3%) | 0 | (0.0%) | 1 | (0.1%) |
|  |  | G2 | 0 | (0.0%) | 1 | (0.3%) | 1 | (0.1%) |
|  | VAGINAL DRYNESS | Total | 7 | (1.8%) | 12 | (3.2%) | 19 | (2.5%) |
|  |  | G1 | 5 | (1.3%) | 9 | (2.4%) | 14 | (1.8%) |
|  |  | G2 | 2 | (0.5%) | 3 | (0.8%) | 5 | (0.7%) |
|  | VAGINAL HEMORRHAGE | Total | 1 | (0.3%) | 3 | (0.8%) | 4 | (0.5%) |
|  |  | G1 | 1 | (0.3%) | 2 | (0.5%) | 3 | (0.4%) |
|  |  | G2 | 0 | (0.0%) | 1 | (0.3%) | 1 | (0.1%) |
|  | VAGINAL PAIN | Total | 2 | (0.5%) | 1 | (0.3%) | 3 | (0.4%) |
|  |  | G1 | 1 | (0.3%) | 1 | (0.3%) | 2 | (0.3%) |
|  |  | G2 | 1 | (0.3%) | 0 | (0.0%) | 1 | (0.1%) |
| RESPIRATORY, THORACIC AND MEDIASTINAL DISORDERS | Total | Total | 148 | (38.5%) | 179 | (47.9%) | 327 | (43.1%) |
|  |  | . | 2 | (0.5%) | 1 | (0.3%) | 3 | (0.4%) |
|  |  | G1 | 130 | (33.9%) | 148 | (39.6%) | 278 | (36.7%) |
|  |  | G2 | 26 | (6.8%) | 57 | (15.2%) | 83 | (10.9%) |
|  |  | G3/4/5 | 3 | (0.8%) | 5 | (1.3%) | 8 | (1.1%) |
|  | ALLERGIC RHINITIS | Total | 1 | (0.3%) | 2 | (0.5%) | 3 | (0.4%) |
|  |  | G1 | 1 | (0.3%) | 2 | (0.5%) | 3 | (0.4%) |
|  | BRONCHIAL FISTULA | Total | 1 | (0.3%) | 0 | (0.0%) | 1 | (0.1%) |
|  |  | G2 | 1 | (0.3%) | 0 | (0.0%) | 1 | (0.1%) |
|  | BRONCHITIS | Total | 1 | (0.3%) | 0 | (0.0%) | 1 | (0.1%) |
|  |  | G2 | 1 | (0.3%) | 0 | (0.0%) | 1 | (0.1%) |
|  | COUGH | Total | 101 | (26.3%) | 114 | (30.5%) | 215 | (28.4%) |
|  |  | . | 1 | (0.3%) | 0 | (0.0%) | 1 | (0.1%) |
|  |  | G1 | 84 | (21.9%) | 89 | (23.8%) | 173 | (22.8%) |
|  |  | G2 | 16 | (4.2%) | 24 | (6.4%) | 40 | (5.3%) |
|  |  | G3/4/5 | 0 | (0.0%) | 1 | (0.3%) | 1 | (0.1%) |
|  | DYSPNEA | Total | 51 | (13.3%) | 69 | (18.4%) | 120 | (15.8%) |
|  |  | . | 1 | (0.3%) | 0 | (0.0%) | 1 | (0.1%) |
|  |  | G1 | 45 | (11.7%) | 47 | (12.6%) | 92 | (12.1%) |
|  |  | G2 | 4 | (1.0%) | 21 | (5.6%) | 25 | (3.3%) |
|  |  | G3/4/5 | 1 | (0.3%) | 1 | (0.3%) | 2 | (0.3%) |
|  | DYSPNEA SHORTNESS OF BREATH | Total | 1 | (0.3%) | 0 | (0.0%) | 1 | (0.1%) |
|  |  | G1 | 1 | (0.3%) | 0 | (0.0%) | 1 | (0.1%) |
|  | EPISTAXIS | Total | 7 | (1.8%) | 41 | (11.0%) | 48 | (6.3%) |
|  |  | G1 | 6 | (1.6%) | 39 | (10.4%) | 45 | (5.9%) |
|  |  | G2 | 0 | (0.0%) | 2 | (0.5%) | 2 | (0.3%) |
|  |  | G3/4/5 | 1 | (0.3%) | 0 | (0.0%) | 1 | (0.1%) |
|  | HOARSENESS | Total | 0 | (0.0%) | 1 | (0.3%) | 1 | (0.1%) |
|  |  | G1 | 0 | (0.0%) | 1 | (0.3%) | 1 | (0.1%) |
|  | HYPOXIA | Total | 0 | (0.0%) | 1 | (0.3%) | 1 | (0.1%) |
|  |  | G1 | 0 | (0.0%) | 1 | (0.3%) | 1 | (0.1%) |
|  | LARYNGEAL INFLAMMATION | Total | 2 | (0.5%) | 1 | (0.3%) | 3 | (0.4%) |
|  |  | G1 | 1 | (0.3%) | 1 | (0.3%) | 2 | (0.3%) |
|  |  | G2 | 1 | (0.3%) | 0 | (0.0%) | 1 | (0.1%) |
|  | NASAL CONGESTION | Total | 3 | (0.8%) | 2 | (0.5%) | 5 | (0.7%) |
|  |  | G1 | 3 | (0.8%) | 1 | (0.3%) | 4 | (0.5%) |
|  |  | G2 | 0 | (0.0%) | 1 | (0.3%) | 1 | (0.1%) |
|  | PHARYNGEAL MUCOSITIS | Total | 1 | (0.3%) | 0 | (0.0%) | 1 | (0.1%) |
|  |  | G1 | 1 | (0.3%) | 0 | (0.0%) | 1 | (0.1%) |
|  | PLEURAL EFFUSION | Total | 0 | (0.0%) | 1 | (0.3%) | 1 | (0.1%) |
|  |  | G2 | 0 | (0.0%) | 1 | (0.3%) | 1 | (0.1%) |
|  | PLEURITIC PAIN | Total | 0 | (0.0%) | 1 | (0.3%) | 1 | (0.1%) |
|  |  | G3/4/5 | 0 | (0.0%) | 1 | (0.3%) | 1 | (0.1%) |
|  | PNEUMONITIS | Total | 5 | (1.3%) | 18 | (4.8%) | 23 | (3.0%) |
|  |  | . | 1 | (0.3%) | 1 | (0.3%) | 2 | (0.3%) |
|  |  | G1 | 0 | (0.0%) | 3 | (0.8%) | 3 | (0.4%) |
|  |  | G2 | 3 | (0.8%) | 13 | (3.5%) | 16 | (2.1%) |
|  |  | G3/4/5 | 1 | (0.3%) | 1 | (0.3%) | 2 | (0.3%) |
|  | PNEUMOTHORAX | Total | 0 | (0.0%) | 1 | (0.3%) | 1 | (0.1%) |
|  |  | G2 | 0 | (0.0%) | 1 | (0.3%) | 1 | (0.1%) |
|  | POSTNASAL DRIP | Total | 1 | (0.3%) | 0 | (0.0%) | 1 | (0.1%) |
|  |  | G2 | 1 | (0.3%) | 0 | (0.0%) | 1 | (0.1%) |
|  | PRODUCTIVE COUGH | Total | 0 | (0.0%) | 1 | (0.3%) | 1 | (0.1%) |
|  |  | G2 | 0 | (0.0%) | 1 | (0.3%) | 1 | (0.1%) |
|  | PULMONARY INFECTION | Total | 0 | (0.0%) | 1 | (0.3%) | 1 | (0.1%) |
|  |  | G2 | 0 | (0.0%) | 1 | (0.3%) | 1 | (0.1%) |
|  | RESPIRATORY FAILURE | Total | 16 | (4.2%) | 6 | (1.6%) | 22 | (2.9%) |
|  |  | G1 | 14 | (3.6%) | 3 | (0.8%) | 17 | (2.2%) |
|  |  | G2 | 2 | (0.5%) | 3 | (0.8%) | 5 | (0.7%) |
|  | SLEEP APNEA | Total | 0 | (0.0%) | 1 | (0.3%) | 1 | (0.1%) |
|  |  | G2 | 0 | (0.0%) | 1 | (0.3%) | 1 | (0.1%) |
|  | SORE THROAT | Total | 5 | (1.3%) | 20 | (5.3%) | 25 | (3.3%) |
|  |  | G1 | 4 | (1.0%) | 15 | (4.0%) | 19 | (2.5%) |
|  |  | G2 | 1 | (0.3%) | 4 | (1.1%) | 5 | (0.7%) |
|  |  | G3/4/5 | 0 | (0.0%) | 1 | (0.3%) | 1 | (0.1%) |
|  | TIGHT CHEST | Total | 0 | (0.0%) | 1 | (0.3%) | 1 | (0.1%) |
|  |  | G1 | 0 | (0.0%) | 1 | (0.3%) | 1 | (0.1%) |
|  | VOICE ALTERATION | Total | 1 | (0.3%) | 0 | (0.0%) | 1 | (0.1%) |
|  |  | G1 | 1 | (0.3%) | 0 | (0.0%) | 1 | (0.1%) |
|  | WHEEZING | Total | 0 | (0.0%) | 2 | (0.5%) | 2 | (0.3%) |
|  |  | G1 | 0 | (0.0%) | 2 | (0.5%) | 2 | (0.3%) |
| SKIN AND SUBCUTANEOUS TISSUE DISORDERS | Total | Total | 131 | (34.1%) | 216 | (57.8%) | 347 | (45.8%) |
|  |  | . | 0 | (0.0%) | 3 | (0.8%) | 3 | (0.4%) |
|  |  | G1 | 113 | (29.4%) | 177 | (47.3%) | 290 | (38.3%) |
|  |  | G2 | 23 | (6.0%) | 54 | (14.4%) | 77 | (10.2%) |
|  |  | G3/4/5 | 4 | (1.0%) | 5 | (1.3%) | 9 | (1.2%) |
|  | ALOPECIA | Total | 15 | (3.9%) | 12 | (3.2%) | 27 | (3.6%) |
|  |  | G1 | 13 | (3.4%) | 11 | (2.9%) | 24 | (3.2%) |
|  |  | G2 | 2 | (0.5%) | 1 | (0.3%) | 3 | (0.4%) |
|  | ANGULAR STOMATITIS | Total | 1 | (0.3%) | 0 | (0.0%) | 1 | (0.1%) |
|  |  | G2 | 1 | (0.3%) | 0 | (0.0%) | 1 | (0.1%) |
|  | BRITTLE NAILS | Total | 0 | (0.0%) | 6 | (1.6%) | 6 | (0.8%) |
|  |  | G1 | 0 | (0.0%) | 5 | (1.3%) | 5 | (0.7%) |
|  |  | G2 | 0 | (0.0%) | 1 | (0.3%) | 1 | (0.1%) |
|  | BULLOUS DERMATITIS | Total | 0 | (0.0%) | 2 | (0.5%) | 2 | (0.3%) |
|  |  | G1 | 0 | (0.0%) | 1 | (0.3%) | 1 | (0.1%) |
|  |  | G2 | 0 | (0.0%) | 1 | (0.3%) | 1 | (0.1%) |
|  | CUTANEOUS LESION | Total | 0 | (0.0%) | 1 | (0.3%) | 1 | (0.1%) |
|  |  | G1 | 0 | (0.0%) | 1 | (0.3%) | 1 | (0.1%) |
|  | DRY SKIN | Total | 35 | (9.1%) | 61 | (16.3%) | 96 | (12.7%) |
|  |  | . | 0 | (0.0%) | 1 | (0.3%) | 1 | (0.1%) |
|  |  | G1 | 33 | (8.6%) | 51 | (13.6%) | 84 | (11.1%) |
|  |  | G2 | 2 | (0.5%) | 9 | (2.4%) | 11 | (1.5%) |
|  | ECZEMA | Total | 7 | (1.8%) | 8 | (2.1%) | 15 | (2.0%) |
|  |  | G1 | 6 | (1.6%) | 7 | (1.9%) | 13 | (1.7%) |
|  |  | G2 | 1 | (0.3%) | 1 | (0.3%) | 2 | (0.3%) |
|  | ERYTHEMA MULTIFORME | Total | 8 | (2.1%) | 15 | (4.0%) | 23 | (3.0%) |
|  |  | G1 | 6 | (1.6%) | 11 | (2.9%) | 17 | (2.2%) |
|  |  | G2 | 2 | (0.5%) | 3 | (0.8%) | 5 | (0.7%) |
|  |  | G3/4/5 | 0 | (0.0%) | 1 | (0.3%) | 1 | (0.1%) |
|  | ERYTHRODERMA | Total | 7 | (1.8%) | 7 | (1.9%) | 14 | (1.8%) |
|  |  | G1 | 3 | (0.8%) | 3 | (0.8%) | 6 | (0.8%) |
|  |  | G2 | 3 | (0.8%) | 3 | (0.8%) | 6 | (0.8%) |
|  |  | G3/4/5 | 1 | (0.3%) | 1 | (0.3%) | 2 | (0.3%) |
|  | FIBROSIS | Total | 0 | (0.0%) | 1 | (0.3%) | 1 | (0.1%) |
|  |  | G1 | 0 | (0.0%) | 1 | (0.3%) | 1 | (0.1%) |
|  | FOLLICULITIS | Total | 2 | (0.5%) | 5 | (1.3%) | 7 | (0.9%) |
|  |  | G1 | 2 | (0.5%) | 5 | (1.3%) | 7 | (0.9%) |
|  | HAIR THINNING | Total | 0 | (0.0%) | 1 | (0.3%) | 1 | (0.1%) |
|  |  | G1 | 0 | (0.0%) | 1 | (0.3%) | 1 | (0.1%) |
|  | HYPERHIDROSIS | Total | 1 | (0.3%) | 0 | (0.0%) | 1 | (0.1%) |
|  |  | G1 | 1 | (0.3%) | 0 | (0.0%) | 1 | (0.1%) |
|  | IRRITATION | Total | 2 | (0.5%) | 0 | (0.0%) | 2 | (0.3%) |
|  |  | G1 | 2 | (0.5%) | 0 | (0.0%) | 2 | (0.3%) |
|  | ITCH | Total | 6 | (1.6%) | 11 | (2.9%) | 17 | (2.2%) |
|  |  | . | 0 | (0.0%) | 1 | (0.3%) | 1 | (0.1%) |
|  |  | G1 | 5 | (1.3%) | 8 | (2.1%) | 13 | (1.7%) |
|  |  | G2 | 1 | (0.3%) | 1 | (0.3%) | 2 | (0.3%) |
|  |  | G3/4/5 | 0 | (0.0%) | 1 | (0.3%) | 1 | (0.1%) |
|  | NAIL DISCOLORATION | Total | 3 | (0.8%) | 1 | (0.3%) | 4 | (0.5%) |
|  |  | G1 | 3 | (0.8%) | 1 | (0.3%) | 4 | (0.5%) |
|  | NAIL LOSS | Total | 3 | (0.8%) | 9 | (2.4%) | 12 | (1.6%) |
|  |  | G1 | 2 | (0.5%) | 8 | (2.1%) | 10 | (1.3%) |
|  |  | G2 | 1 | (0.3%) | 1 | (0.3%) | 2 | (0.3%) |
|  | NAIL RIDGING | Total | 9 | (2.3%) | 14 | (3.7%) | 23 | (3.0%) |
|  |  | . | 0 | (0.0%) | 1 | (0.3%) | 1 | (0.1%) |
|  |  | G1 | 9 | (2.3%) | 12 | (3.2%) | 21 | (2.8%) |
|  |  | G2 | 0 | (0.0%) | 1 | (0.3%) | 1 | (0.1%) |
|  | PAIN OF SKIN | Total | 2 | (0.5%) | 1 | (0.3%) | 3 | (0.4%) |
|  |  | G1 | 1 | (0.3%) | 1 | (0.3%) | 2 | (0.3%) |
|  |  | G2 | 1 | (0.3%) | 0 | (0.0%) | 1 | (0.1%) |
|  | PALMAR-PLANTAR ERYTHRODYSESTHESIA SYNDROME | Total | 1 | (0.3%) | 2 | (0.5%) | 3 | (0.4%) |
|  |  | G1 | 1 | (0.3%) | 0 | (0.0%) | 1 | (0.1%) |
|  |  | G2 | 0 | (0.0%) | 2 | (0.5%) | 2 | (0.3%) |
|  | PIN HEAD SPOTS | Total | 0 | (0.0%) | 1 | (0.3%) | 1 | (0.1%) |
|  |  | G1 | 0 | (0.0%) | 1 | (0.3%) | 1 | (0.1%) |
|  | PRICKLY HEAT | Total | 0 | (0.0%) | 1 | (0.3%) | 1 | (0.1%) |
|  |  | G1 | 0 | (0.0%) | 1 | (0.3%) | 1 | (0.1%) |
|  | PRURITUS | Total | 19 | (4.9%) | 44 | (11.8%) | 63 | (8.3%) |
|  |  | G1 | 14 | (3.6%) | 36 | (9.6%) | 50 | (6.6%) |
|  |  | G2 | 4 | (1.0%) | 8 | (2.1%) | 12 | (1.6%) |
|  |  | G3/4/5 | 1 | (0.3%) | 0 | (0.0%) | 1 | (0.1%) |
|  | PSORIASIS | Total | 0 | (0.0%) | 1 | (0.3%) | 1 | (0.1%) |
|  |  | G1 | 0 | (0.0%) | 1 | (0.3%) | 1 | (0.1%) |
|  | RASH | Total | 40 | (10.4%) | 103 | (27.5%) | 143 | (18.9%) |
|  |  | G1 | 37 | (9.6%) | 77 | (20.6%) | 114 | (15.0%) |
|  |  | G2 | 3 | (0.8%) | 25 | (6.7%) | 28 | (3.7%) |
|  |  | G3/4/5 | 0 | (0.0%) | 1 | (0.3%) | 1 | (0.1%) |
|  | SCALP PAIN | Total | 2 | (0.5%) | 0 | (0.0%) | 2 | (0.3%) |
|  |  | G1 | 2 | (0.5%) | 0 | (0.0%) | 2 | (0.3%) |
|  | SEBORRHEA | Total | 1 | (0.3%) | 0 | (0.0%) | 1 | (0.1%) |
|  |  | G1 | 1 | (0.3%) | 0 | (0.0%) | 1 | (0.1%) |
|  | SKIN DISORDERS | Total | 1 | (0.3%) | 3 | (0.8%) | 4 | (0.5%) |
|  |  | . | 0 | (0.0%) | 1 | (0.3%) | 1 | (0.1%) |
|  |  | G1 | 1 | (0.3%) | 2 | (0.5%) | 3 | (0.4%) |
|  | SKIN HYPERPIGMENTATION | Total | 0 | (0.0%) | 3 | (0.8%) | 3 | (0.4%) |
|  |  | G1 | 0 | (0.0%) | 2 | (0.5%) | 2 | (0.3%) |
|  |  | G3/4/5 | 0 | (0.0%) | 1 | (0.3%) | 1 | (0.1%) |
|  | SKIN HYPOPIGMENTATION | Total | 0 | (0.0%) | 3 | (0.8%) | 3 | (0.4%) |
|  |  | G1 | 0 | (0.0%) | 3 | (0.8%) | 3 | (0.4%) |
|  | SKIN INDURATION | Total | 1 | (0.3%) | 0 | (0.0%) | 1 | (0.1%) |
|  |  | G3/4/5 | 1 | (0.3%) | 0 | (0.0%) | 1 | (0.1%) |
|  | SKIN ULCERATION | Total | 2 | (0.5%) | 7 | (1.9%) | 9 | (1.2%) |
|  |  | G1 | 2 | (0.5%) | 6 | (1.6%) | 8 | (1.1%) |
|  |  | G2 | 0 | (0.0%) | 1 | (0.3%) | 1 | (0.1%) |
|  | SPOT | Total | 2 | (0.5%) | 0 | (0.0%) | 2 | (0.3%) |
|  |  | G1 | 2 | (0.5%) | 0 | (0.0%) | 2 | (0.3%) |
|  | SWEAT | Total | 1 | (0.3%) | 0 | (0.0%) | 1 | (0.1%) |
|  |  | G1 | 1 | (0.3%) | 0 | (0.0%) | 1 | (0.1%) |
|  | TELANGIECTASIA | Total | 2 | (0.5%) | 1 | (0.3%) | 3 | (0.4%) |
|  |  | G1 | 1 | (0.3%) | 1 | (0.3%) | 2 | (0.3%) |
|  |  | G2 | 1 | (0.3%) | 0 | (0.0%) | 1 | (0.1%) |
|  | TINGLING | Total | 2 | (0.5%) | 1 | (0.3%) | 3 | (0.4%) |
|  |  | G1 | 2 | (0.5%) | 1 | (0.3%) | 3 | (0.4%) |
|  | TOXIC EPIDERMAL NECROLYSIS | Total | 1 | (0.3%) | 0 | (0.0%) | 1 | (0.1%) |
|  |  | G2 | 1 | (0.3%) | 0 | (0.0%) | 1 | (0.1%) |
|  | TOXICODERMIA | Total | 0 | (0.0%) | 2 | (0.5%) | 2 | (0.3%) |
|  |  | G1 | 0 | (0.0%) | 1 | (0.3%) | 1 | (0.1%) |
|  |  | G2 | 0 | (0.0%) | 1 | (0.3%) | 1 | (0.1%) |
|  | URTICARIA | Total | 2 | (0.5%) | 2 | (0.5%) | 4 | (0.5%) |
|  |  | G1 | 1 | (0.3%) | 1 | (0.3%) | 2 | (0.3%) |
|  |  | G2 | 1 | (0.3%) | 1 | (0.3%) | 2 | (0.3%) |
|  | VAC FOR SKIN NECROSIS | Total | 1 | (0.3%) | 0 | (0.0%) | 1 | (0.1%) |
|  |  | G3/4/5 | 1 | (0.3%) | 0 | (0.0%) | 1 | (0.1%) |
| SURGICAL AND MEDICAL PROCEDURES | Total | Total | 13 | (3.4%) | 4 | (1.1%) | 17 | (2.2%) |
|  |  | . | 2 | (0.5%) | 1 | (0.3%) | 3 | (0.4%) |
|  |  | G1 | 4 | (1.0%) | 0 | (0.0%) | 4 | (0.5%) |
|  |  | G2 | 6 | (1.6%) | 2 | (0.5%) | 8 | (1.1%) |
|  |  | G3/4/5 | 1 | (0.3%) | 1 | (0.3%) | 2 | (0.3%) |
|  | ANNEXECTOMY | Total | 1 | (0.3%) | 0 | (0.0%) | 1 | (0.1%) |
|  |  | G1 | 1 | (0.3%) | 0 | (0.0%) | 1 | (0.1%) |
|  | BREAST RECONSTRUCTION | Total | 1 | (0.3%) | 1 | (0.3%) | 2 | (0.3%) |
|  |  | . | 0 | (0.0%) | 1 | (0.3%) | 1 | (0.1%) |
|  |  | G1 | 1 | (0.3%) | 0 | (0.0%) | 1 | (0.1%) |
|  | CARDIAC RADIOFREQUENCY ABLATION | Total | 1 | (0.3%) | 0 | (0.0%) | 1 | (0.1%) |
|  |  | G2 | 1 | (0.3%) | 0 | (0.0%) | 1 | (0.1%) |
|  | CATARACT OPERATION | Total | 1 | (0.3%) | 1 | (0.3%) | 2 | (0.3%) |
|  |  | G3/4/5 | 1 | (0.3%) | 1 | (0.3%) | 2 | (0.3%) |
|  | CYST EXERESE | Total | 1 | (0.3%) | 0 | (0.0%) | 1 | (0.1%) |
|  |  | G2 | 1 | (0.3%) | 0 | (0.0%) | 1 | (0.1%) |
|  | HAND SURGERY | Total | 0 | (0.0%) | 1 | (0.3%) | 1 | (0.1%) |
|  |  | G2 | 0 | (0.0%) | 1 | (0.3%) | 1 | (0.1%) |
|  | LOBECTOMY (THYROID) | Total | 1 | (0.3%) | 0 | (0.0%) | 1 | (0.1%) |
|  |  | G2 | 1 | (0.3%) | 0 | (0.0%) | 1 | (0.1%) |
|  | NODULE EXCISION | Total | 1 | (0.3%) | 0 | (0.0%) | 1 | (0.1%) |
|  |  | G2 | 1 | (0.3%) | 0 | (0.0%) | 1 | (0.1%) |
|  | PARATHYROIDECTOMY | Total | 1 | (0.3%) | 0 | (0.0%) | 1 | (0.1%) |
|  |  | G2 | 1 | (0.3%) | 0 | (0.0%) | 1 | (0.1%) |
|  | SALPINGECTOMY | Total | 1 | (0.3%) | 0 | (0.0%) | 1 | (0.1%) |
|  |  | . | 1 | (0.3%) | 0 | (0.0%) | 1 | (0.1%) |
|  | SCAR RESECTION | Total | 1 | (0.3%) | 0 | (0.0%) | 1 | (0.1%) |
|  |  | . | 1 | (0.3%) | 0 | (0.0%) | 1 | (0.1%) |
|  | SKIN LESION REMOVED | Total | 1 | (0.3%) | 0 | (0.0%) | 1 | (0.1%) |
|  |  | G1 | 1 | (0.3%) | 0 | (0.0%) | 1 | (0.1%) |
|  | THICKENING SURGICAL SCAR | Total | 1 | (0.3%) | 0 | (0.0%) | 1 | (0.1%) |
|  |  | G1 | 1 | (0.3%) | 0 | (0.0%) | 1 | (0.1%) |
|  | TOOTH EXTRACTION | Total | 1 | (0.3%) | 1 | (0.3%) | 2 | (0.3%) |
|  |  | G2 | 1 | (0.3%) | 1 | (0.3%) | 2 | (0.3%) |
| VASCULAR DISORDERS | Total | Total | 106 | (27.6%) | 99 | (26.5%) | 205 | (27.0%) |
|  |  | . | 0 | (0.0%) | 2 | (0.5%) | 2 | (0.3%) |
|  |  | G1 | 77 | (20.1%) | 54 | (14.4%) | 131 | (17.3%) |
|  |  | G2 | 30 | (7.8%) | 40 | (10.7%) | 70 | (9.2%) |
|  |  | G3/4/5 | 10 | (2.6%) | 15 | (4.0%) | 25 | (3.3%) |
|  | ANKLE ULCER | Total | 0 | (0.0%) | 1 | (0.3%) | 1 | (0.1%) |
|  |  | G1 | 0 | (0.0%) | 1 | (0.3%) | 1 | (0.1%) |
|  | FLUSHING | Total | 3 | (0.8%) | 3 | (0.8%) | 6 | (0.8%) |
|  |  | G1 | 2 | (0.5%) | 3 | (0.8%) | 5 | (0.7%) |
|  |  | G2 | 1 | (0.3%) | 0 | (0.0%) | 1 | (0.1%) |
|  | HEAVY FEELING IN ARMS & LEGS | Total | 0 | (0.0%) | 1 | (0.3%) | 1 | (0.1%) |
|  |  | G1 | 0 | (0.0%) | 1 | (0.3%) | 1 | (0.1%) |
|  | HEMATOMA | Total | 0 | (0.0%) | 1 | (0.3%) | 1 | (0.1%) |
|  |  | G1 | 0 | (0.0%) | 1 | (0.3%) | 1 | (0.1%) |
|  | HOT FLASHES | Total | 72 | (18.8%) | 30 | (8.0%) | 102 | (13.5%) |
|  |  | G1 | 60 | (15.6%) | 24 | (6.4%) | 84 | (11.1%) |
|  |  | G2 | 12 | (3.1%) | 6 | (1.6%) | 18 | (2.4%) |
|  | HYPERTENSION | Total | 29 | (7.6%) | 39 | (10.4%) | 68 | (9.0%) |
|  |  | . | 0 | (0.0%) | 1 | (0.3%) | 1 | (0.1%) |
|  |  | G1 | 7 | (1.8%) | 10 | (2.7%) | 17 | (2.2%) |
|  |  | G2 | 14 | (3.6%) | 18 | (4.8%) | 32 | (4.2%) |
|  |  | G3/4/5 | 8 | (2.1%) | 10 | (2.7%) | 18 | (2.4%) |
|  | HYPOTENSION | Total | 1 | (0.3%) | 1 | (0.3%) | 2 | (0.3%) |
|  |  | G1 | 1 | (0.3%) | 1 | (0.3%) | 2 | (0.3%) |
|  | LYMPHEDEMA | Total | 16 | (4.2%) | 33 | (8.8%) | 49 | (6.5%) |
|  |  | G1 | 11 | (2.9%) | 18 | (4.8%) | 29 | (3.8%) |
|  |  | G2 | 5 | (1.3%) | 14 | (3.7%) | 19 | (2.5%) |
|  |  | G3/4/5 | 0 | (0.0%) | 1 | (0.3%) | 1 | (0.1%) |
|  | LYMPHOCELE | Total | 1 | (0.3%) | 0 | (0.0%) | 1 | (0.1%) |
|  |  | G2 | 1 | (0.3%) | 0 | (0.0%) | 1 | (0.1%) |
|  | SWELLING | Total | 1 | (0.3%) | 1 | (0.3%) | 2 | (0.3%) |
|  |  | G1 | 1 | (0.3%) | 0 | (0.0%) | 1 | (0.1%) |
|  |  | G2 | 0 | (0.0%) | 1 | (0.3%) | 1 | (0.1%) |
|  | VARICOSE | Total | 0 | (0.0%) | 3 | (0.8%) | 3 | (0.4%) |
|  |  | . | 0 | (0.0%) | 1 | (0.3%) | 1 | (0.1%) |
|  |  | G1 | 0 | (0.0%) | 1 | (0.3%) | 1 | (0.1%) |
|  |  | G2 | 0 | (0.0%) | 1 | (0.3%) | 1 | (0.1%) |
|  | VASCULAR DISORDERS - OTHER, SPECIFY | Total | 1 | (0.3%) | 0 | (0.0%) | 1 | (0.1%) |
|  |  | G3/4/5 | 1 | (0.3%) | 0 | (0.0%) | 1 | (0.1%) |
|  | VENOUS THROMBOTIC EVENT | Total | 1 | (0.3%) | 9 | (2.4%) | 10 | (1.3%) |
|  |  | G2 | 0 | (0.0%) | 4 | (1.1%) | 4 | (0.5%) |
|  |  | G3/4/5 | 1 | (0.3%) | 5 | (1.3%) | 6 | (0.8%) |
|  |  |  |  |  |  |  |  |  |

**Supplementary Table 3. Grade 3-4 events according to endocrine therapy backbone.**

|  | **TAMOXIFEN**  N=505 | | | | | | | | **AROMATASE INHIBITOR**  N=773 | | | | | | | |
| --- | --- | --- | --- | --- | --- | --- | --- | --- | --- | --- | --- | --- | --- | --- | --- | --- |
|  | **Tamoxifen-Placebo**  N=253 (%) | | | | **Tamoxifen-Everolimus**  N=252 (%) | | | | **Aromatase Inhibitor-Placebo**  N=388 (%) | | | | **Aromatase Inhibitor-Everolimus**  N=385 (%) | | | |
| CTCAE Grade | 3 | | 4 | | 3 | | 4 | | 3 | | 4 | | 3 | | 4 | |
| **GASTROINTESTINAL DISORDERS** | | | | | | | | | | | | | | | | |
| MUCOSITIS ORAL | 1 | (0.4%) | 0 | - | 19 | (7.5%) | 0 | - | 1 | (0.3%) | 0 | - | 27 | (7.0%) | 0 | - |
| DIARRHEA | 2 | (0.8%) | 0 | - | 3 | (1.2%) | 0 | - | 0 | - | 0 | - | 3 | (0.8%) | 0 | - |
| NAUSEA | 2 | (0.8%) | 0 | - | 1 | (0.4%) | 0 | - | 1 | (0.3%) | 0 | - | 1 | (0.3%) | 0 | - |
| **GENERAL DISORDERS** | | | | | | | | | | | | | | | | |
| FATIGUE | 4 | (1.6%) | 0 | - | 3 | (1.2%) | 0 | - | 4 | (1.0%) | 0 | - | 9 | (2.3%) | 0 | - |
| I**NVESTIGATIONS** | | | | | | | | | | | | | | | | |
| CHOLESTEROL HIGH | 0 | - | 0 | - | 2 | (0.8%) | 1 | (0.4%) | 0 | - | 0 | - | 2 | (0.5%) | 0 | - |
| LIVER TESTS ELEVATION (ALT/AST) | 3 | (1.2%) | 0 | - | 7 | (2.8%) | 1 | (0.4%) | 6 | (1.5%) | 2 | (0.5%) | 6 | (1.6%) | 0 | - |
| LYMPHOCYTE COUNT DECREASED | 2 | (0.8%) | 0 | - | 3 | (1.2%) | 0 | - | 2 | (0.5%) | 0 | - | 3 | (0.8%) | 0 | - |
| NEUTROPHIL COUNT DECREASED | 1 | (0.4%) | 1 | (0.4%) | 2 | (0.8%) | 0 | - | 1 | (0.3%) | 0 | - | 3 | (0.8%) | 0 | - |
| **METABOLISM DISORDERS** | | | | | | | | | | | | | | | | |
| HYPERTRIGLYCERIDEMIA | 1 | (0.4%) | 0 | - | 9 | (3.6%) | 5 | (2.0%) | 0 | - | 0 | - | 5 | (1.3%) | 0 | - |
| HYPERGLYCEMIA | 1 | (0.4%) | 0 | - | 3 | (1.2%) | 0 | - | 0 | - | 0 | - | 6 | (1.6%) | 0 | - |
| **MUSCULOSKELETAL DISORDERS** | | | | | | | | | | | | | | | | |
| ARTHRALGIA | 0 | - | 0 | - | 0 | - | 0 | - | 1 | (0.3%) | 0 | - | 2 | (0.5%) | 0 | - |
| **RESPIRATORY DISORDERS** | | | | | | | | | | | | | | | | |
| COUGH | 1 | (0.4%) | 0 | - | 0 | - | 0 | - | 1 | (0.4%) | 0 | - | 1 | (0.3%) | 0 | - |
| DYSPNEA | 0 | - | 0 | - | 1 | (0.4%) | 0 | - | 0 | - | 0 | - | 1 | (0.3%) | 0 | - |
| RESPIRATORY FAILURE | 0 | - | 0 | - | 0 | - | 0 | - | 0 | - | 0 | - | 0 | - | 0 | - |
| PNEUMONITIS | 0 | - | 0 | - | 4 | (1.6%) | 0 | - | 1 | (0.3%) | 0 | - | 1 | (0.3%) | 0 | - |
| **VASCULAR DISORDERS** | | | | | | | | | | | | | | | | |
| HOT FLASHES | 2 | (0.8%) | 0 | - | 1 | (0.4%) | 0 | - | 0 | - | 0 | - | 0 | - | 0 | - |
| HYPERTENSION | 4 | (1.6%) | 0 | - | 5 | (2.0%) | 0 | - | 8 | (2.1%) | 0 | - | 10 | (2.6%) | 0 | - |
| VENOUS THROMBOTIC EVENT | 0 | - | 0 | - | 2 | (0.8%) | 1 | (0.4%) | 1 | (0.3%) | 0 | - | 4 | (1.0%) | 1 | (0.3%) |

**Supplementary Table 4. Populations and key results in recent adjuvant studies in high-risk HR+/HER2- early breast cancer (Percentages may not add up to 100% due to missing information).**

|  | UNIRAD | SWOG 1207 | NATALEE | MONARCH-E | SOFT/TEXT |
| --- | --- | --- | --- | --- | --- |
| N | 1278 | 1761 | 5101 | 5637 | 3066/2672 |
| Node positive (%)  ≥4N+ (%) | 100  52.7 | 91  40 | 59.7  18.6 | 98.8  59.6 | 34.5/48.2  Uk^1^ |
| Stage (%)  II  III |  |  | 40.1  59.6 | 25.8  74 |  |
| Premenopausal (%) | 32.3 | 32 | 43.9 | 43.5 | 100 |
| Endocrine therapy (%)  Tamoxifen  Tamoxifen + OFS  AI + OFS  AI | 43.6  -  0.5  56 | 25.2  8.4  65.9 | -  -  43.9  56.1 | 31.4  7.6  14.2  68.3 | 33.2^2^/-  40.1^3^  40.9^3^  - |
| Delay to inclusion^4^ | ≤ 4y ET | ≤ 42w CT | ≤12 m ET | ≤12w ET  ≤16m surgery | ≤12w surgery |
| Dose reduction^5^ (%) | T: 34.9  AI: 33.8 | 32 | 21.9 | 41.2 | - |
| Discontinuation^6^ (%) | T: 48.0  AI: 56.9 | 52 | 33.8 | 16.6 | T^7^: 22.5  T-OFS: 18.5  AI-OFS: 27.8 |
| DFS  Control arm  Experimental arm | 3y  88  89 | 5y  74.4  74.9 | 3y  87.6  90.7 | 3y  84.4  89.0 | 8y  T: 78.9  T-OFS: 82.8  AI-OFS: 86.8 |

^1^ unknown

^2^ applicable to the SOFT trial only

^3^ applicable to the combined analysis of the SOFT and TEXT trials

^4^ refers to the delay between last treatment (ET: initiation of endocrine therapy; CT: chemotherapy; m: months; w: weeks)

^5^ refers to the occurrence of at least one dose reduction of the targeted therapy, except for the SOFT/TEXT trials (T: tamoxifen; AI: aromatase inhibitor)

^6^ refers to discontinuation of the targeted therapy, except for the SOFT/TEXT trials (T: tamoxifen; AI: aromatase inhibitor)

^7^ T: tamoxifen; T-OFS: tamoxifen + ovarian function suppression; AI-OFS ; aromatase inhibitor + ovarian function suppression
