# Supplementary material for: Differential benefit of adjuvant everolimus according to endocrine therapy backbone in the randomized UNIRAD trial
Source: ESMO Open. 2025 Apr 15;10(5):105050. doi: 10.1016/j.esmoop.2025.105050 (PMC12020834; doi:10.1016/j.esmoop.2025.105050)
Supplement: Supplementary Figure 1 [file mmc2.pptx]

## Slide 1
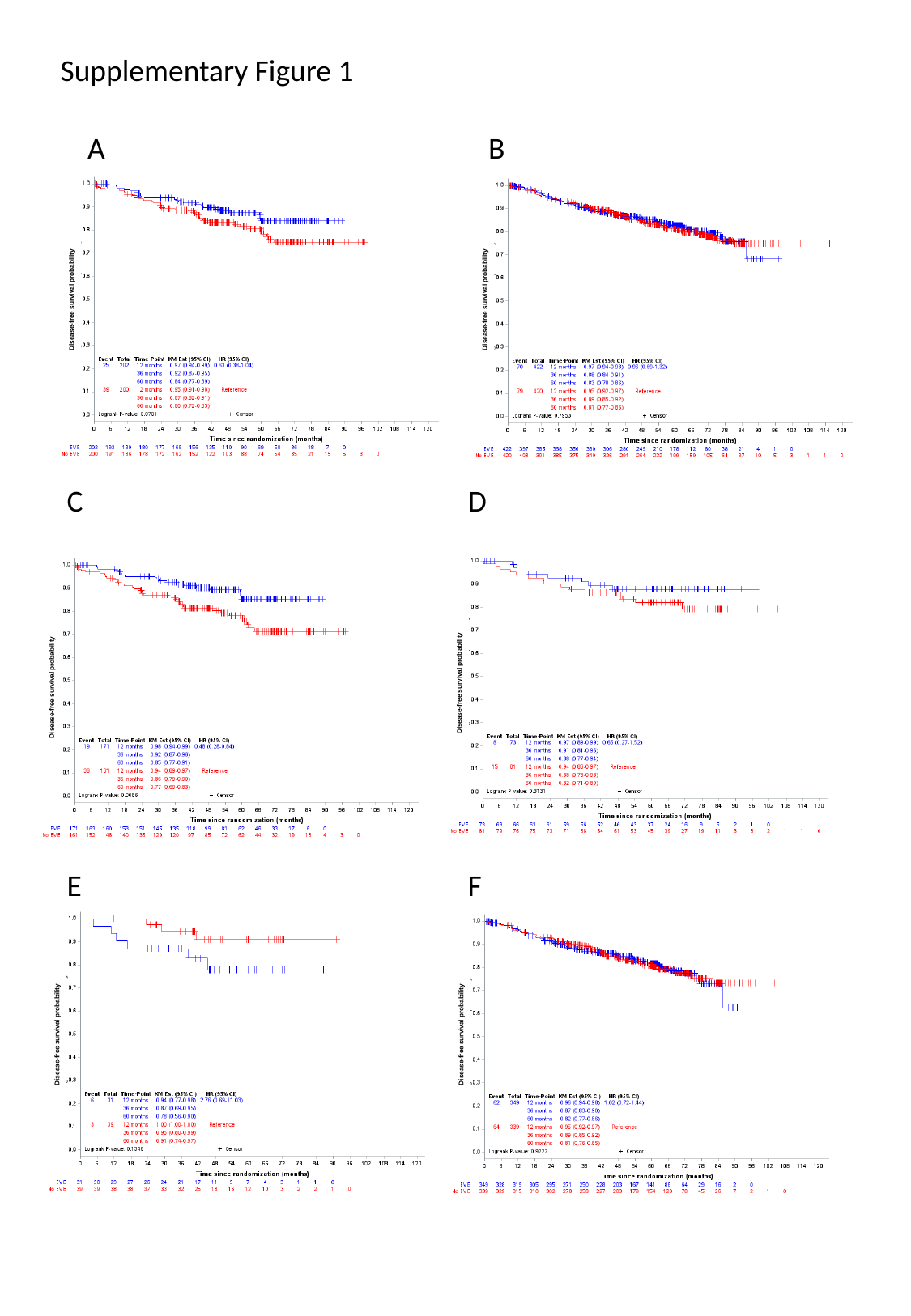

Supplementary Figure 1
A
B
Disease-free survival probability
Disease-free survival probability
C
D
Disease-free survival probability
Disease-free survival probability
E
F
Disease-free survival probability
Disease-free survival probability
Everolimus-endocrine therapy
Placebo-endocrine therapy
