# Supplementary material for: Differential benefit of adjuvant everolimus according to endocrine therapy backbone in the randomized UNIRAD trial
Source: ESMO Open. 2025 Apr 15;10(5):105050. doi: 10.1016/j.esmoop.2025.105050 (PMC12020834; doi:10.1016/j.esmoop.2025.105050)
Supplement: Supplementary Figure 2 [file mmc3.pptx]

## Slide 1
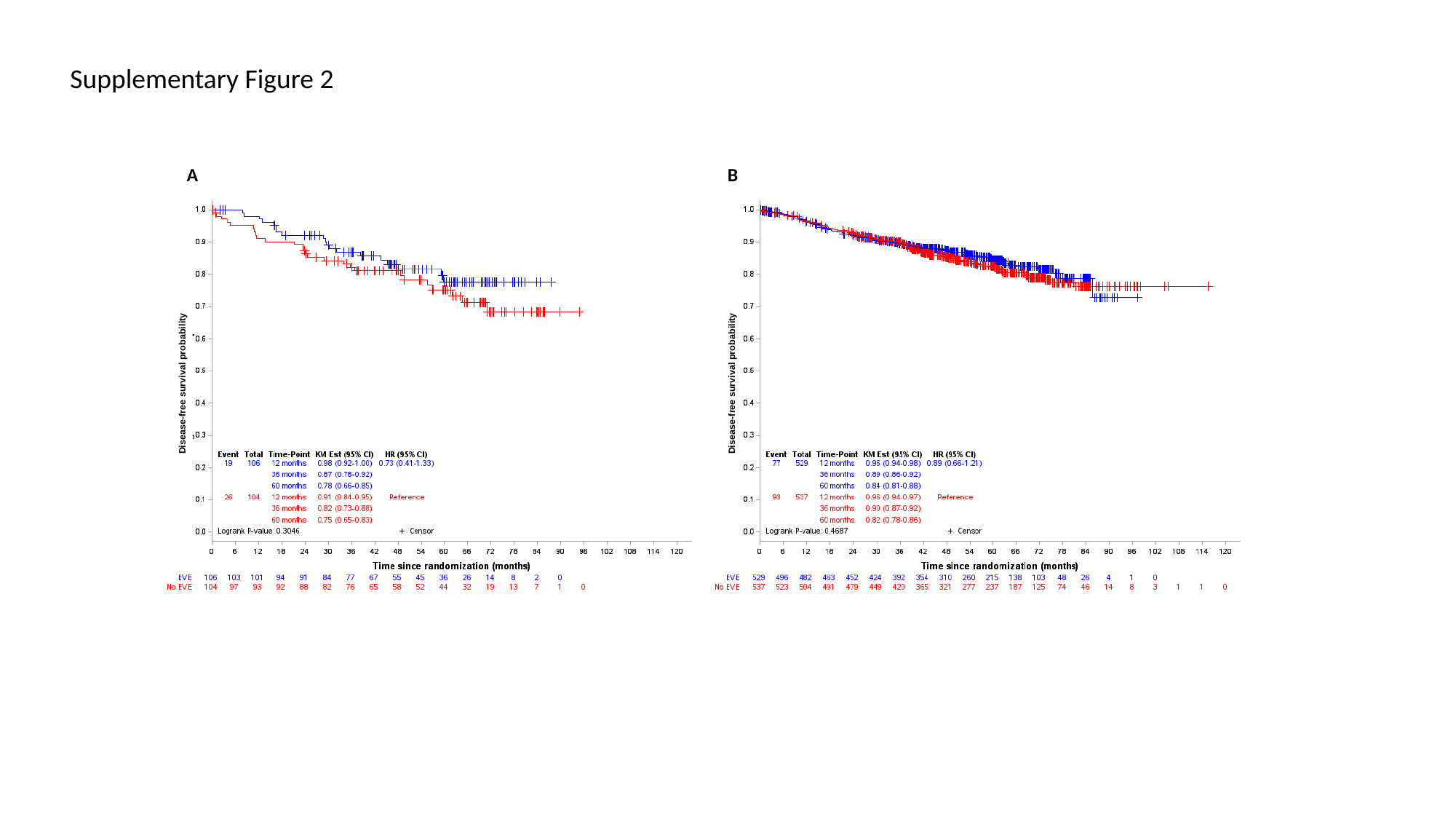

Supplementary Figure 2
A
B
Disease-free survival probability
Disease-free survival probability
